# Promiscuous Transferases Malonylate Furaneol Glucoside in *Fragaria* × *ananassa*

**DOI:** 10.1021/acs.jafc.5c09017

**Published:** 2026-02-05

**Authors:** Martha Purnami Wulanjati, Johanna Trinkl, Xiran Wang, Thomas Hoffmann, Wilfried Schwab

**Affiliations:** † Biotechnology of Natural Products, TUM School of Life Sciences, 9184 Technical University of Munich, 85354 Freising, Germany; ‡ Research Center for Food Technology and Processing (PRTPP), National Research and Innovation Agency (BRIN), 55861 Yogyakarta, Indonesia

**Keywords:** malonyltransferases, glycosides, acylation, malonylation

## Abstract

Acylation is essential in plant metabolism, protecting metabolites from enzymatic degradation, aiding xenobiotic detoxification, and regulating cellular uptake. It also enhances the stability, solubility, and bioactivity of natural products, making it valuable for drug discovery. Since HDMF (4-hydroxy-2,5-dimethyl-3­(2*H*)-furanone; Furaneol) 6′-*O*-malonyl glucoside was detected in strawberries, we hypothesized that strawberry malonyltransferases (FaMATs) acylate HDMF glucoside. Genome analysis of *Fragaria* × *ananassa* and biochemical assays identified FaMAT1C, FaMAT1S, and FaMAT4C_1_/S_1_ as enzymes catalyzing its malonylation, producing three isomers—likely due to keto–enol tautomerism. A screening revealed the broad substrate tolerance of FaMATs, with successful malonylation observed in 67 structurally different glycosides. Notably, FaMAT4C_1_/S_1_ malonylated maple furanone glucoside at the 6-OH position of the glucose moiety resulted in previously unknown metabolites. This modification stabilizes glycosides by preventing glycosidic bond cleavage by glycosidases. Understanding FaMAT function deepens insights into plant specialized metabolism and supports the development of natural product-based therapeutics.

## Graphical abstract

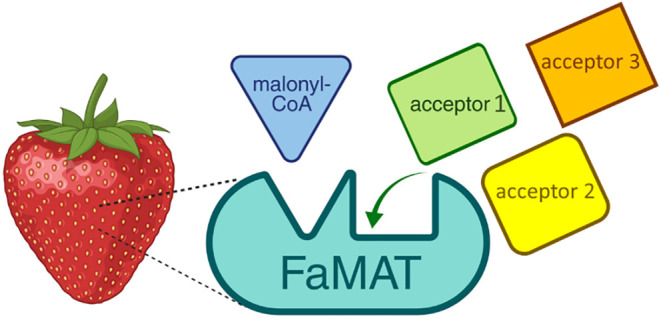

## Introduction

Plant glycosides are promising candidates for pharmaceutical and nutraceutical applications^1^ as flavonoid glycosides, for example, exhibit various bioactivities, including antioxidant, anti-inflammatory, anticancer, and antidiabetic properties.^2,3^ Furthermore, the glycosylated gallic acid, β-glucogallin, is known for antidiabetic activity by inhibiting aldose reductase,^4^ and HDMF (4-hydroxy-2,5-dimethyl-3­(2*H*)-furanone) glucoside, 5-EHMF (5-ethyl-4-hydroxy-2-methyl-3­(2*H*)-furanone) glucoside, sotolon glucoside, and maple furanone glucoside are considered profragrances and proflavors.^5,6^

Some glycosides occur in acylated form in plants^7–9^ where they play significant roles in plant growth, reproduction, and defense against biotic and abiotic stresses.^10^ Aliphatic acylation, especially malonylation, can prevent degradation by β-glucosidase, stabilize the metabolite structures, detoxify xenobiotic compounds, and promote the transport and storage of metabolites.^11–13^ In natural product-based drug development, acylation of glycosides is crucial as it increases their biological activities by improving their stability, bioavailability, and cell membrane permeability.^14^ Acylation of flavonoid glycosides enhances their solubility and stability in lipophilic media, which benefits their application in drug and nutraceutical formulation.^15^

A superfamily of enzymes known as BAHD/HXXXD acyltransferases is involved in acylation in plants and mainly contributes to the biosynthesis of secondary metabolites for the production of aromatic esters and amides, constitutive defense compounds, and phytoalexins.^10,16,17^ BAHD/HXXXD acyltransferases utilize acyl-CoAs as donors to transfer the acyl groups onto acceptor molecules.^16^ BAHD/HXXXD acyltransferases share the conserved HXXXD domain, which is responsible for catalytic activity, and DFGW/FG, which probably has a structural function rather than a catalytic role.^18,19^

Malonyltransferases (MAT), a member of the BAHD/HXXXD acyltransferases family, likely contribute to polyphenol biosynthesis in strawberry (*Fragaria* × *ananassa*), as several malonylated glycosides have been identified in strawberry fruits, including cyanidin 3-*O*-(6′-*O*-malonyl) glucoside, pelargonidin 3-*O*-(6′-*O*-malonyl) glucoside, quercetin 3-*O*-(6′-*O*-malonyl) glucoside, kaempferol 3-*O*-(6′-*O*-malonyl) glucoside, and peonidin 3-*O*-(6′-*O-*malonyl) glucoside.^20–22^ Malonylated HDMF glucoside has also been detected in strawberry fruit.^23^

Furaneol or 4-hydroxy-2,5-dimethyl-3­(2*H*)-furanone (HDMF) is a major flavor compound in strawberry fruit^24^ and is also found in other fruits such as grape,^25^ pineapple,^26^ kiwi,^27^ and tomato.^28^ Starting from d-fructose-1,6-diphosphate,^29,30^ 4-hydroxy-5-methyl-2-methylene-3­(2*H*)-furanone (HMMF) is first formed via an as yet unknown mechanism, which is then converted into HDMF by an enone oxidoreductase (FaEO).^31,32^ The methylation and glucosylation of HDMF is catalyzed by an *O*-methyltransferase^33^ and glucosyltransferases,^6^ respectively. A MAT enzyme presumably forms HDMF malonyl glucoside.

In this study, potential ripening-related *MAT* genes were identified in the genome of the woodland strawberry (*Fragaria vesca*), and their orthologs were isolated from *Fragaria* × *ananassa* var. Candonga (C) and Senga Sengana (S). The recombinant FaMAT1C, FaMAT1S, and FaMAT4C_1_/S_1_, were functionally characterized and shown to acylate kaempferol 3-*O*-glucoside, quercetin 3-*O*-glucoside, cyanidin 3-*O*-glucoside, and pelargonidin 3-*O*-glucoside. The FaMATs were screened with 67 different glycosides, which differed in the aglycone as well as glycone part, whereby the previously undescribed enzymatic formation of HDMF (6′-*O*-malonyl) glucoside and other natural and non-natural acylated glycosides could be demonstrated. The stability of malonylated glycosides against hydrolysis was tested with almond β-glucosidase. The characterized FaMATs are promiscuous enzymes that can malonylate structurally related glycosides and, thus, contribute significantly to the biodiversity of the strawberry metabolome.

## Materials and Methods

### Plants, Chemicals, and Enzymes

The *Fragaria* × *ananassa* “Candonga” and “Senga Sengana” were supplied by Hansabred GmbH & Co KG. All plant materials were stored at −80 °C until use. Restriction enzymes and T4 DNA ligase were purchased from Thermo Fisher Scientific (Vilnius, Lithuania). Malonyl Coenzyme A lithium salt was purchased from Cayman Chemical (Michigan). Acetyl-CoA, acryloyl-CoA, crotonyl-CoA, *p*-coumaroyl-CoA, feruloyl-CoA, and 4-hydroxybenzoyl-CoA were kindly provided by MicroCombichem GmbH (Frankfurt, Germany). Glycoside substrates were purchased from Sigma-Aldrich (Darmstadt, Germany), Extrasynthese (Genay, France), and Carl Roth (Karlsruhe, Germany), isolated by Prof. Geiger from various plants, and produced in-house using glucosyltransferases.^5,34^ Other reagents, including β-glucosidase from almonds, were purchased from Sigma-Aldrich (Darmstadt, Germany).

### DNA Extraction and Cloning Procedure

Genomic DNA was extracted from 50 mg of homogenized plant material, either fruit or young leaves, using the DNeasy Plant Mini Kit (Qiagen, Venlo, The Netherlands), following the manufacturer’s protocol. Elution was performed with 100 μL of elution buffer for fresh leaf samples and 50 μL for fruit samples. Candidate *MAT* genes were amplified via PCR using primers listed in Table S1. The resulting PCR products were purified using the NucleoSpin Gel and PCR Clean-up Kit (Macherey-Nagel, Schwerte, Germany). Purified amplicons were ligated into the pGEM-T Easy Vector (Promega, Walldorf, Germany) using a 1:3 molar ratio of vector to insert, as recommended by the manufacturer. Ligation reactions were incubated at 4 °C for 72 h and subsequently transformed into *Escherichia coli* XL-1 Blue (Agilent Technologies, Waldbronn, Germany).

To construct recombinant plasmids for expression, *MAT* genes from pGEM-T recombinant were amplified using specific primers introducing restriction sites *Bam*HI and *Not*I (Table S1). The amplicons were inserted into a pGEX4T-1 (GE Healthcare, Munich, Germany) and transformed into *E. coli* NEB-10 β (New England Biolabs, Frankfurt am Main, Germany). Recombinant plasmids pGEX4T-1 carrying *MAT* genes were then transformed into *E. coli* BL21 (New England Biolabs, Frankfurt am Main, Germany) according to the manufacturer’s instructions.

### Recombinant FaMAT Protein Expression

Recombinant *E. coli* BL21 harboring plasmid pGEX4T-1-FaMAT1C, pGEX4T-1-FaMAT4C_1_/S_1_, or pGEX4T-1-FaMAT1S were inoculated in LB media containing 100 μg mL^–1^ ampicillin and grown at 37 °C and 150 rpm to an optical density of 1.0 at 600 nm. Expression of GST-tagged proteins was induced by 1 mM isopropyl β-d-thiogalactopyranoside (IPTG) at 20 °C for 18 h. Cell pellets were harvested by centrifugation at 5500*g* for 15 min and 4 °C. Pellets were resuspended in GST washing buffer (4.3 mM Na_2_HPO_4_, 14.7 mM KH_2_PO_4_, 0.137 M NaCl, 2.7 mM KCl, pH 7.3) and 20 mM phenylmethylsulfonyl fluoride (PMSF) before being lysed by ultrasonication. Supernatants were separated by centrifugation at 13,000*g* for 30 min at 4 °C. Proteins were purified using GST Bind Resin (Merck, Darmstadt, Germany). Supernatants were loaded onto the resin and washed by using GST washing buffer. GST-tagged proteins were eluted using GST elution buffer (10 mM reduced glutathione, 50 mM Tris/HCl pH 8.0). The recombinant protein concentration was estimated by comparing the band intensity on SDS-PAGE gels to known amounts of bovine serum albumin. Measurements were performed using the G:BOX gel documentation system (Syngene, Cambridge, U.K.) and analyzed with GeneTools software (Syngene, Cambridge, U.K.). The concentration of FaMATs was then determined from a standard curve generated with bovine serum albumin.

### In Vitro Enzymatic Assay

Malonyltransferase enzymatic assay was adopted from Luo et al.^11^ and slightly modified. The reaction mixture comprised 20 mM potassium phosphate buffer, pH 7.0, 0.6 mM acyl-CoA, 0.6 mM acyl acceptor, 0.5 mM EDTA, 5 mM β-mercaptoethanol, and 2.5 μg of purified FaMAT protein. The mixture was incubated at 30 °C for 18 h. The reaction was terminated by adding a double amount of methanol. In addition, the specific activities of FaMATs toward apigenin-6-*C*-glucoside, apigenin-7-*O*-glucoside, quercetin-3-*O*-glucoside, pelargonidin-3-*O*-glucoside, aesculin, thymol *O*-glucoside, and furanone glucosides were determined using 25 μg FaMATs, incubated at 30 °C for 30 min. This allowed for a direct comparison of the initial rates. The sample was analyzed by LC-ESI-MS using Agilent 1100 Series HPLC with Luna C-18 column (150 mm × 2 mm × 3 μm, 100 Å, Phenomenex, California) connected to an Esquire 3000 Plus Ion Trap mass spectrometer (Bruker Daltonics, Bremen, Germany). The mobile phase comprised solvent A (0.1% HCOOH in H_2_O) and solvent B (0.1% HCOOH in CH_3_OH), with a gradient system as follows: 0–50% solvent B in 30 min, 50–100% solvent B in 5 min, 100% solvent B for 15 min, 100–0% solvent B in 5 min, and 0% solvent B for 10 min. The flow rate was 0.2 mL min^–1^. Specifications for the mass spectrometer were as follows: Helium was used as the collision gas, N_2_ as the spray gas, capillary temperature 330 °C, and scan range from *m*/*z* 50–975. The percentage of conversion was calculated based on the peak area of products and substrate in the UV chromatogram, assuming that the extinction coefficient of the product was the same as that of the substrate.^35^ Products and substrates that cannot be detected using a DAD detector, such as monoterpenol and alkyl glycosides, were calculated based on the peak area in the LC-MS extracted ion chromatogram (EIC). The amount of substrate remaining in the sample was calculated by plotting the peak area on the standard curve function of the substrate. The product amount was calculated by subtracting the remaining substrate amount in the sample from that of the substrate control without protein.

### Optimization Assay and Enzyme Kinetic Study

The optimal conditions of the enzymatic assay were determined for the parameters incubation time, pH value, and reaction temperature using quercetin 3-*O*-glucoside as the acyl acceptor, malonyl-CoA as the acyl donor, and 2.5 μg of FaMATs. The optimum incubation duration was determined from 15 min until 18 h at pH 7.0 and incubation temperature of 30 °C. The optimum pH was assessed in the range pH 3.0–5.0 (citric acid-sodium citrate buffer), pH 6.0–8.0 (potassium phosphate buffer), and pH 9.2–10.4 (sodium carbonate-sodium bicarbonate buffer). The optimum temperature was determined from 5 °C until 50 °C, at pH 7.0. In addition, the optimal incubation time using HDMF *O*-glucoside was determined with 25 μg of FaMATs at pH 7.0 and 30 °C, ranging from 5 min to 8 h.

Kinetic parameters for FaMATs were conducted using furanone glucosides such as HDMF *O*-glucoside, 5-EHMF *O*-glucoside, maple furanone *O*-glucoside, and sotolon *O*-glucoside. The reaction comprised a serial concentration of acceptors from 100 to 2500 μM, a fixed concentration of 600 μM malonyl-CoA, and 25 μg of FaMATs. Reactions were performed at 30 °C for 30 min. The products were analyzed by LC-ESI-MS. The product quantities were determined using the peak areas of the acylated glycosides in the UV chromatogram and the standard curve function of glycosides. It was assumed that the extinction coefficient of the acylated product is identical to that of the substrate. The parameters *K*_M_ and *k*_cat_ were determined using the Michaelis–Menten diagram.

### Hydrolysis Assay by β-Glucosidase toward Malonylated Product

The solutions for the hydrolysis reaction consist of the corresponding malonylation reaction sample, 40 mM potassium phosphate buffer pH 6.0, and almond β-glucosidase 16 U.^36^ The hydrolysis reaction was carried out at 50 °C for 2 h and terminated at 95 °C for 15 min.^37^ The samples were analyzed by LC-ESI-MS.

### Isolation and Identification of Malonylated Maple Furanone Glucoside

The malonylated product of maple furanone glucoside was synthesized by an in vitro enzymatic assay using FaMAT4C_1_/S_1_. The malonylated maple furanone glucoside was isolated using an HPLC System Goebel Analytik (Hallertau, Germany) equipped with a column Zorbax Eclipse XDB-C8 (150 mm × 4.6 mm). The first separation was performed using an isocratic mobile phase of 5% ethanol with 0.1% formic acid and the second separation using isocratic mobile phase of 2% ethanol with 10 mM ammonium formate, flow rate 1.5 mL min^–1^, λ = 232 nm, and sample injection volume was 10 μL. The fractions containing the malonylated products were freeze-dried. The dried extracts were dissolved in DMSO-*d*_6_, and subsequently analyzed by solution-state NMR spectroscopy on a 1.2 GHz Bruker Avance Neo Cryo spectrometer (Bruker Biospin GmbH, Ettlingen, Germany).

## Results and Discussion

### Isolation of Candidate *MAT* Genes and Production of Recombinant Proteins

An in-house transcriptome data set of *F. vesca* cv. Reine des Vallées revealed five *MAT* genes expressed in fruit tissues.^38,39^ To investigate their potential role in the malonylation of glycosides during strawberry fruit ripening, the *MAT* candidate genes 04261 (*MAT1*), 04262 (*MAT2*), 03835 (*MAT3*), 29347 (*MAT4*), and 04266 (*MAT5*) were isolated from two *Fragaria × ananassa* cultivars, “Senga Sengana” (S) and “Candonga” (C). These genes were cloned into an *E. coli* expression system, and the corresponding recombinant proteins were produced. Three MAT proteins, namely, FaMAT1C (isolated from cv Candonga), FaMAT1S (isolated from cv Senga Sengana, orthologous to FaMAT1C), and FaMAT4C_1_/S_1_ (identical sequences isolated from Candonga and Senga Sengana) catalyzed the acylation of flavonoid glycosides and anthocyanins such as kaempferol 3-*O*-glucoside **16**, quercetin 3-*O*-glucoside **12**, cyanidin 3-*O*-glucoside **38**, and pelargonidin 3-*O*-glucoside **36**. FaMAT1C and FaMAT1S show 99.4% sequence identity (Figure S1). They differ in only three amino acids. The sequence similarity and identity between FaMAT1C and FaMAT4C_1_/S_1_ are 70.9% and 58.6%, respectively, and between FaMAT1S and FaMAT4C_1_/S_1_ are 71.1% and 59.0%, respectively. Phylogenetic analysis by Wang et al.^40^ showed that FaMATs are closely related to anthocyanin 5-*O*-glucoside-6″-*O*-malonyltransferase from *Arabidopsis thaliana* (At5MAT) (NP_189600.1) and malonyltransferases from *Glycine max* (AQY54373.1) and *Medicago truncatula* (ABY91221.1, ADV04046.1, ABY91222.1, and ABY91220.1).

The FaMAT amino acid sequences contain the three conserved domains of BAHD/HXXXD acyltransferases that are responsible for their functions. Motif 1 HXXXD contributes to the catalysis of acyl transfer. The histidine of motif 1 deprotonates the hydroxyl group of the acceptor molecules, which triggers a nucleophilic attack on the thioester of the acyl donor.^41^ Sequence motif 1 in FaMAT1C and FaMAT1S is HAVLD, and thus differs from FaMAT4C_1_/S_1_, which has the sequence HAILD. The YF/KGNC/A motif is found in anthocyanin acyltransferase and is part of the substrate binding site, while DFGW/FG motif is thought to be responsible for maintaining the conformational integrity of protein.^42^ All FaMATs have identical YFGNC and DFGWG motifs.

### FaMAT Acylate HDMF Glucoside and Other Furanone Glucosides

Since HDMF (6′-*O*-malonyl) glucoside is a natural component of strawberry fruit, we tested HDMF glucoside as a substrate for the FaMATs. FaMAT1C, FaMAT1S, and FaMAT4C_1_/S_1_ successfully transferred malonic acid from malonyl-CoA to HDMF glucoside. Three products were formed, which featured pseudomolecular ions at *m*/*z* 377 [M + H]^+^ and could be separated chromatographically (Figure 1). The MS fragmentation pattern of each product showed the cleavage of HDMF with *m*/*z* = 129 [M + H]^+^ (Figure 1b). The ratios of the compounds produced by the FaMAT enzymes were nearly identical. Compound 1 showed the lowest signal, while isomers 2 and 3 showed similar signal areas.

**Figure 1:**
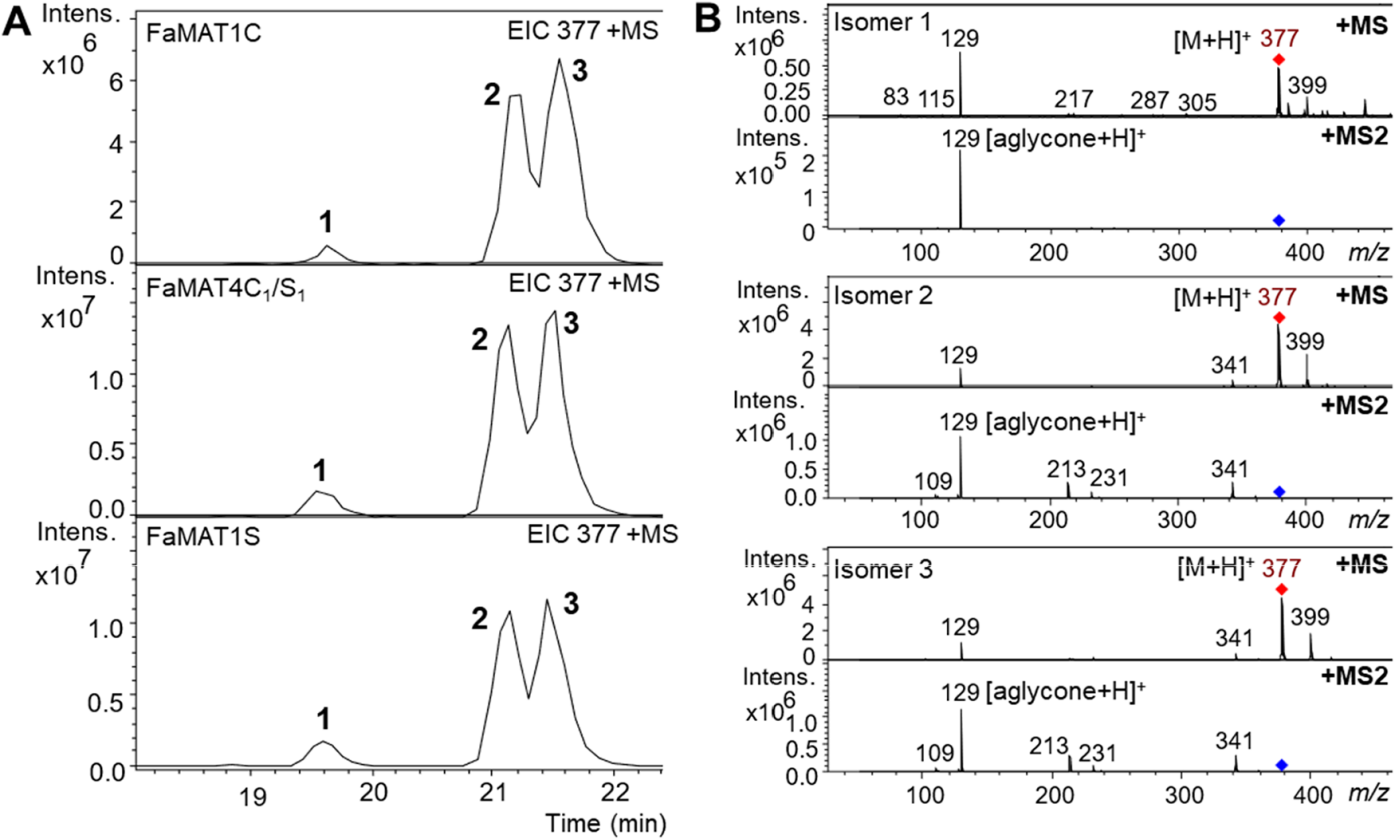
LC-MS analysis of products formed by FaMAT1C, FaMAT1S, and FaMAT4C_1_/S_1_ from HDMF glucoside and malonyl-CoA. Extracted ion chromatogram (EIC) of malonylated products (A). MS and MS^2^ fragmentation patterns of isomers 1–3 (B).

The diastereomeric mixture of HDMF (6′-*O-*malonyl) glucoside was first isolated from strawberries in 1996 and attributed to the keto–enol tautomerism of HDMF.^23^ A study showed that the HDMF enantiomers are stable at a pH of 4–5 but rapidly racemize at a neutral pH due to tautomerism (Figure 2A).^43,44^ Based on the peak ratio of the three isomers, we assume that signals 2 and 3, which elute later, are the two diastereomers of the keto–enol form, in which the malonyl group is bound to the 6-OH position of the glucosyl (Figure 2B). Isomer 1 was then the dienol derivative. HDMF is known to racemize rapidly, resulting in the formation of a diastereomeric mixture of HDMF glucosides. These observations demonstrate that tautomerization occurs prior to malonylation.^44^ Alternatively, the malonyl group could be bound to a position other than 6-OH of the glucosyl group. As will be demonstrated later, malonylation of other monoglucosides—such as apigenin-6-*C*-glucoside **1** and kaempferol 7-*O*-glucoside **24**, resulted in the formation of two isomeric products. This observation suggests that the malonyl group can be attached to different hydroxyl positions on the glucosyl moiety. Dm3MAT2, a MAT that malonylates anthocyanins at position 6” of the sugar, also catalyzes malonylation at position 3”.^45^

**Figure 2:**
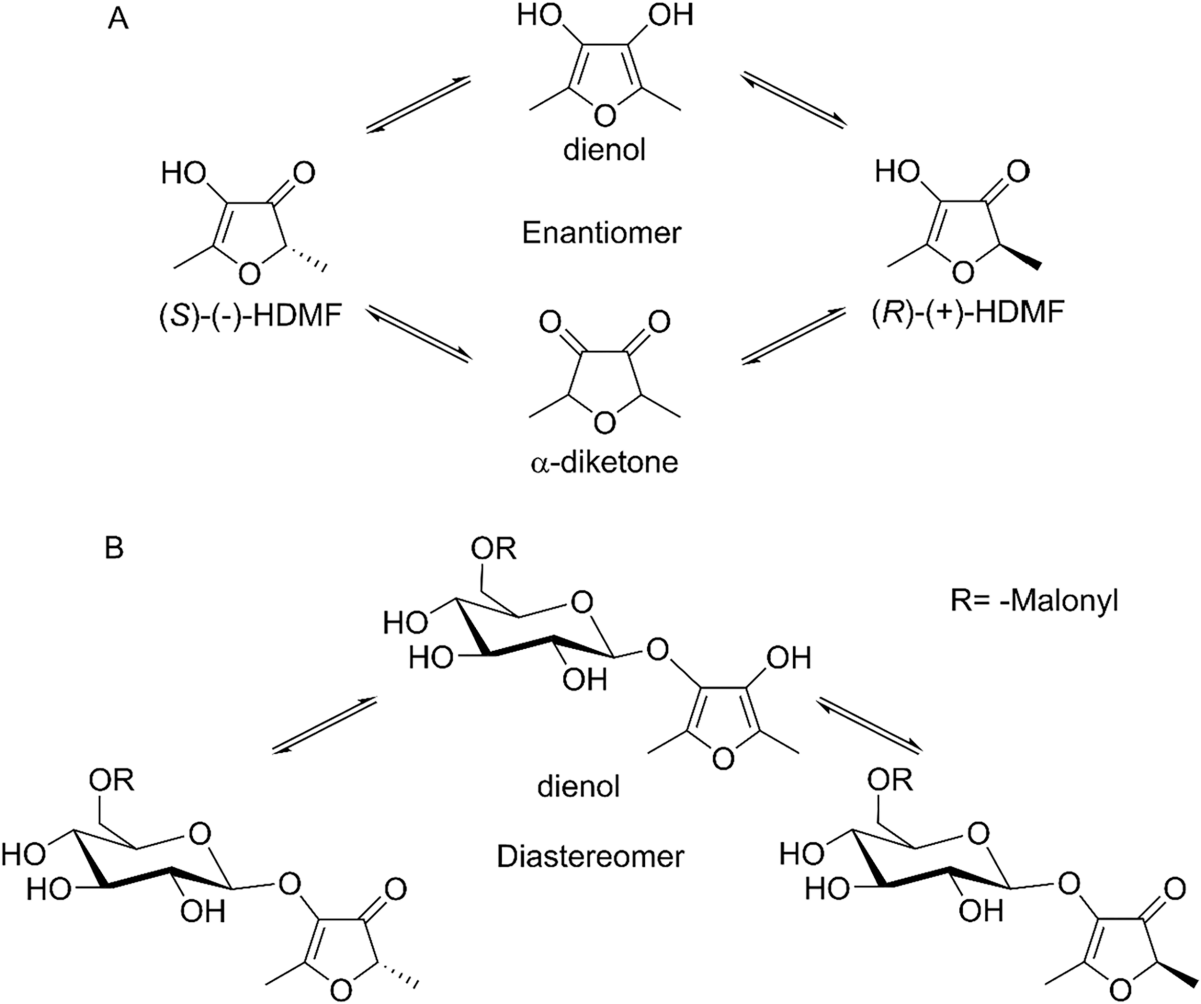
Racemization of HDMF due to keto–enol tautomerism and its implications. Keto–enol equilibrium of HDMF isomers^43^ (A). Three isomeric HDMF malonyl glucosides can be derived from the HDMF isomers (B).

We determined the activity of FaMATs toward other furanone glucosides structurally related to HDMF glucoside. These included 5-EHMF (2­(or 5)-ethyl-4-hydroxy-5­(or 2)-methyl-3­(2*H*)-furanone) glucoside, also known as homofuraneol glucoside; 3-hydroxy-4,5-dimethylfuran-2­(5*H*)-one glucoside, commonly referred to as sotolon glucoside; and 2-ethyl-3-hydroxy-4-methyl-5H-furan-2-one glucoside also known as maple furanone glucoside (Figures S2–S3). Each substrate, similar to HDMF glucoside, was converted to three isomeric products with the pseudomolecular ion *m*/*z* 391 [M + H]^+^ for 5-EHMF malonyl glucoside (Figure S2), *m*/*z* 399 [M + Na]^+^ for sotolon malonyl glucoside (Figure S2), and *m*/*z* 413 [M + Na]^+^ for maple furanone malonyl glucoside (Figure S3). The product ion spectra (MS2) showed that each malonyl glucoside released its aglycone, namely, 5-EHMF with *m*/*z* 143 [M + H]^+^ (Figure S2), sotolon with *m*/*z* 151 [M + Na]^+^ (Figure S2), and maple furanone with *m*/*z* 165 [M + Na]^+^ (Figure S3).

As in the case of HDMF malonyl glucoside, the three isomeric compounds are likely to represent a pair of diastereomers and a regioisomer. Similar to the racemization of HDMF, 5-EHMF undergoes keto–enol isomerization to produce the dienol, keto–enol, and diketone forms.^45^ The absolute configurations of the 5-EHMF enantiomers were determined as (*R*)-(+)-5-EHMF and (*S*)-(−)-5-EHMF (Figure S3)^46^ and the sotolon enantiomers as (*R*)-(−)-sotolon and (*S*)-(+)-sotolon, as well as the maple furanone enantiomers as (*R*)-(+)-maple furanone and (*S*)-(−)-maple furanone (Figure S3).^47^

### Effect of pH Value, Temperature, and Incubation Time as well as Kinetic Investigation of FaMATs

To determine the effect of pH, temperature, and incubation time on the enzymatic activity of FaMATs, we utilized quercetin-3-*O*-glucoside as the acyl acceptor, malonyl-CoA as the acyl donor, and 2.5 μg of FaMATs (Figure S4A–C). Quercetin-3-*O*-glucoside and its malonylated product are naturally occurring glycosides in strawberries.^48,49^ At pH values of 3.0–4.0 and 9.7–10.4, the FaMATs produced little to no quercetin-3-*O*-(6′-*O*-malonyl) glucoside. The maximum amount of product is formed at pH 6.0–8.0 for FaMAT1C and FaMAT1S and at pH 6.4–7.4 for FaMAT4C_1_/S_1_. The optimum temperature for FaMAT1C and FaMAT1S is 20–40 °C, for FaMAT4C_1_/S_1_ 30–40 °C. The investigation of the incubation time with quercetin-3-*O*-glucoside showed that the equilibrium state, in which the rate of the forward reaction corresponds to the reverse reaction, is reached after 2 h of incubation for FaMAT1C and FaMAT1S and after 6 h of incubation for FaMAT4C_1_/S_1_. A time-course experiment using 25 μg of FaMATs and HDMF *O*-glucoside as the acyl acceptor showed that the maximum product yield was achieved after 1 hour of incubation with FaMAT4C1/S1 and FaMAT1S, and after 2 hours with FaMAT1C (Figure S4D). Notably, following 1 hour of incubation, the product concentration in reactions with FaMAT4C1/S1 and FaMAT1S gradually declined, while the substrate concentration increased, suggesting a possible reverse reaction.^50^

To evaluate the catalytic efficiency of FaMATs toward various furanone glucosides, enzyme assays were conducted using 0.60 mM malonyl-CoA and increasing concentrations of acyl acceptor substrates at pH 7.0 and 30 °C for 30 min. Kinetic parameters were derived from Michaelis–Menten plots (Figure S5). Among the FaMAT proteins, FaMAT1S exhibited the lowest *K*_M_ value (0.37 mM) and the highest catalytic efficiency (*k*_cat_/*K*_M_ = 8.2 mM^–1^·min^–1^) for HDMF glucoside (Table 1). FaMAT4C_1_/S_1_ exhibited slightly lower catalytic efficiency toward HDMF glucoside (*k*_cat_/*K*_M_ = 7.1 mM^–1^·min^–1^) compared to FaMAT1S. FaMAT4C_1_/S_1_ demonstrated superior binding affinity and catalytic efficiency for other furanone glucosides, including 5-EHMF glucoside, sotolon glucoside, and maple furanone glucoside, outperforming the other FaMAT variants. These results indicate that FaMAT4C_1_/S_1_ is the most efficient enzyme for converting these substrates to their corresponding malonylated products. The *K*_M_ values of the FaMATs toward furanones vary from 0.37 mM to greater than 2.5 mM and the *k*_cat_ values from 1.2 min^–1^ to 7.4 min^–1^. These values are in a similar range to those for an anthocyanidin malonyltransferase from *A. thaliana* with *K*_M_ = 0.167 mM and *k*_cat_ = 1.2 min^–1^ for cyanidin glucoside and *K*_M_ = 0.07 mM and *k*_cat_ = 7.2 min^–1^ for the proposed natural substrate cyanidin 3-*O*-[2″-*O*-(xylosyl)-6″-*O*-(4-*O*-(glucosyl)-4-coumaroyl) glucoside]­5-*O*-glucoside.^51^

**Table 1:** Kinetic Data of FaMATs toward Furanone Glucosides

substrates	protein	*K*_M_ (mM)	*k*_cat_ (min^–1^)	*k*_cat_/*K*_M_ (mM^–1^ min^–1^)
HDMF glucoside	FaMAT1C	0.77 ± 0.2	3.4 ± 0.4	4.4
	FaMAT4C_1_/S_1_	0.43 ± 0.1	3.0 ± 0.1	7.1
	FaMAT1S	0.37 ± 0.1	3.0 ± 0.2	8.2
5-EHMF glucoside	FaMAT1C	2.35 ± 0.5	7.4 ± 0.8	3.1
	FaMAT4C_1_/S_1_	0.55 ± 0.1	6.4 ± 0.3	11.5
	FaMAT1S	0.83 ± 0.1	5.6 ± 0.2	6.7
maple furanone glucoside	FaMAT1C	>2.5	na^a^	na
	FaMAT4C_1_/S_1_	0.40 ± 0.0	4.2 ± 0.1	10.3
	FaMAT1S	0.66 ± 0.1	4.1 ± 0.3	6.2
sotolon glucoside	FaMAT1C	>2.5	na	na
	FaMAT4C_1_/S_1_	1.09 ± 0.2	3.1 ± 0.3	2.8
	FaMAT1S	1.31 ± 0.3	1.2 ± 0.1	0.9

a Not available.

### FaMATs Exhibit Substrate Promiscuity with Various Types of Glycosides

To investigate the substrate preferences and donor specificities of FaMATs in more detail, 87 different glycosides with different sugar components were incubated with malonyl-CoA as an acyl donor. In the study, 67 substrates were converted into malonylated glycosides (Figure 3). The glycosides that were malonylated consisted of different aglycones, such as flavones (**1**-**7**), flavonols (**8**-**31**), flavanones (**32**-**33**), isoflavones (**34**-**35**), anthocyanins (**36**-**40**), coumarins (**41**-**45**), phenolics (**46**-**53**), anthraquinones (**54**), furanones (**55**-**58**), pyranones (**59**-**60**), phenylpropenoids (**61**), monoterpenols (**62**-**63**), alkyls (**64**-**65**), lignan (**66**), and anthranilic acid (**67**) (Figures 4 and 5, Table S3, Figures S6–S26).

**Figure 3:**
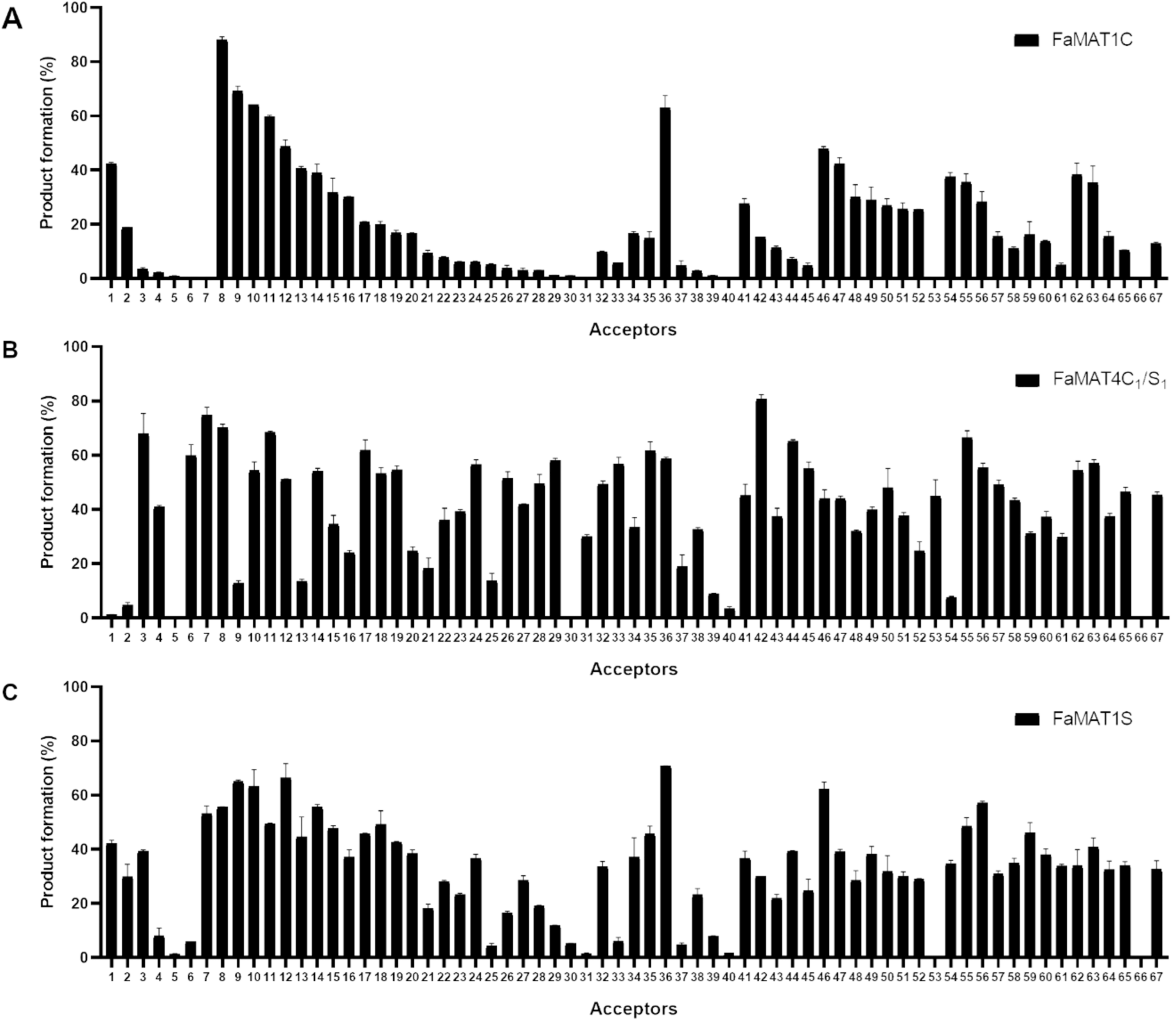
Quantification of malonylated glycosides formed from acceptor substrates **1**-**67** by LC. The structures of acceptors **1**-**67** are shown in Figures 4 and 5. Product formation (%) of glycosides was catalyzed by FaMAT1C (**A**), FaMAT4C_1_/S_1_ (**B**), and FaMAT1S (**C**).

**Figure 4:**
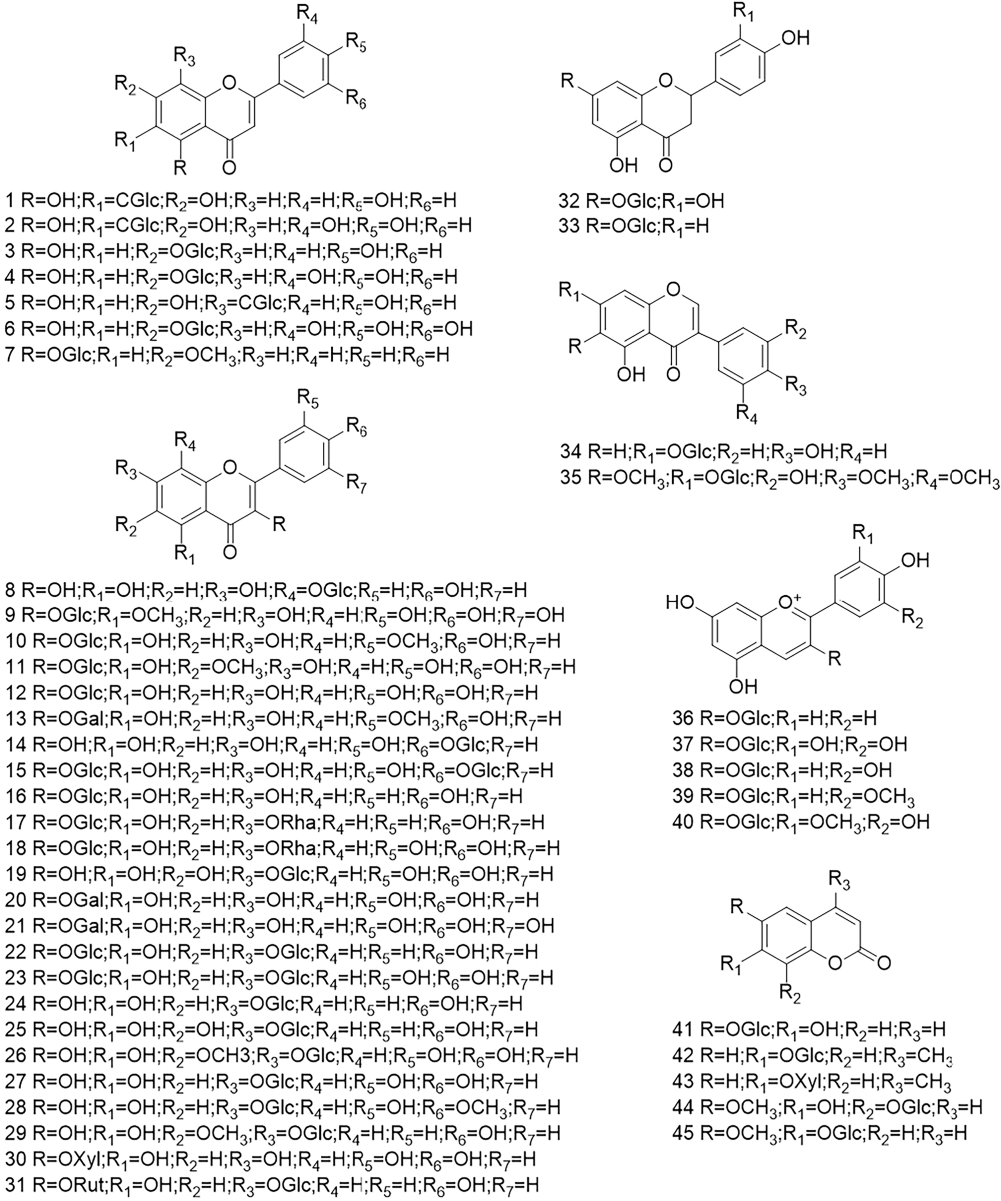
Chemical structures of acceptors. Flavone glycosides (**1**-**7**), flavonol glycosides (**8**-**31**), flavanone glycosides (**32**-**33**), isoflavone glycosides (**34**-**35**), anthocyanin glycosides (**36**-**40**), and coumarin glycosides (**41**-**45**). The chemical names and trivial names are given in Table S3.

**Figure 5:**
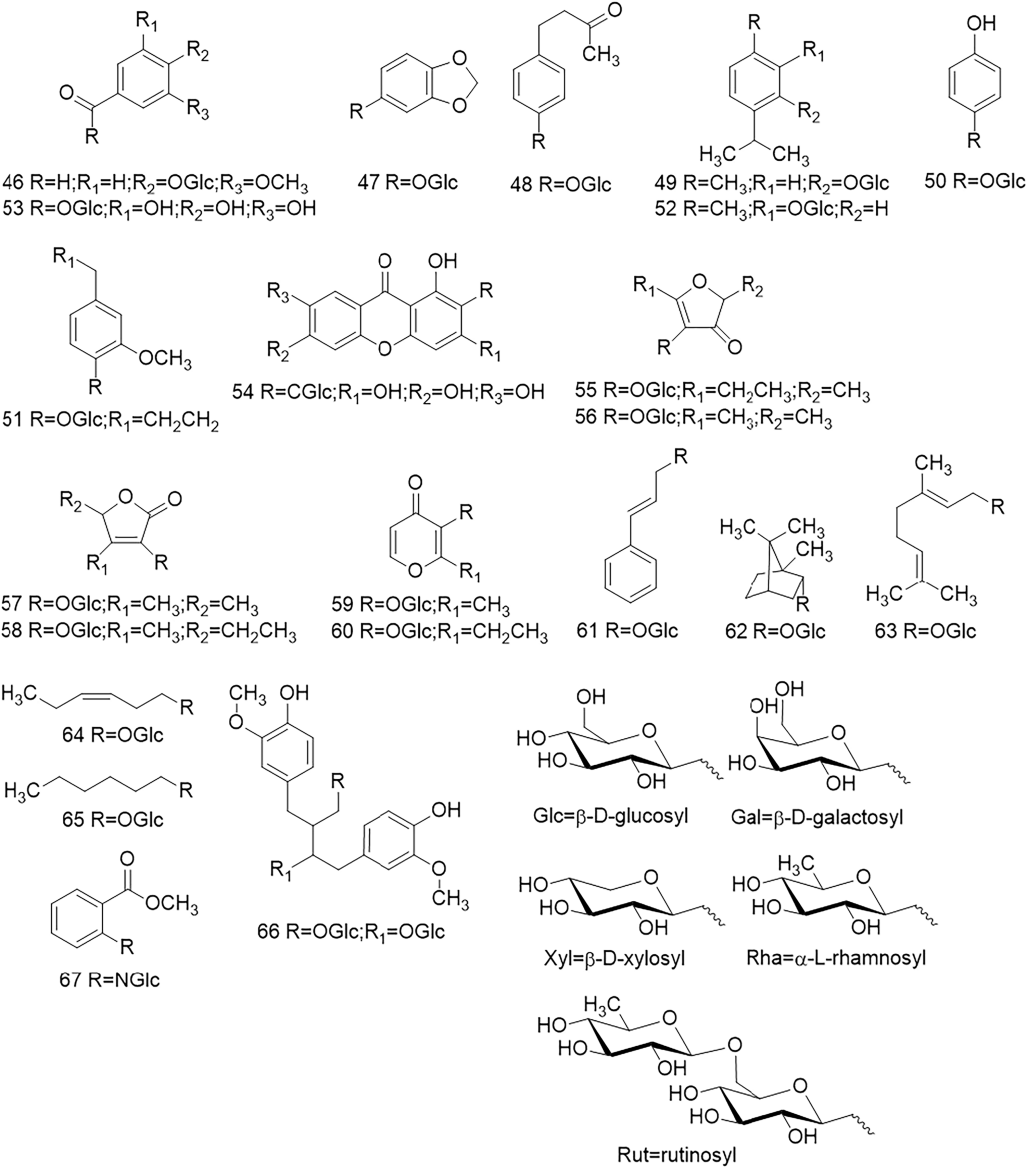
Chemical structures of acceptors. Phenolic glycosides (**46**-**53**), anthraquinone glycoside (**54**), furanone glycosides (**55**-**58**), pyranone glycosides (**59**-**60**), phenylpropenyl glycoside (**61**), monoterpenol glycosides (**62**-**63**), alkyl glycosides (**64**-**65**), lignan glycoside (**66**), and benzoic acid ester glycoside (**67**). The chemical names and trivial names are listed in Table S3.

This result underscored that FaMATs show a high substrate acceptance. The natural substrates of FaMATs are likely flavonoid glucosides and furanone glucosides, as their malonic acid derivatives are found in strawberry fruits. MATs that malonylate furanone glucosides, pyranone glucosides, phenylpropenyl glucosides, monoterpenol glucosides, alkyl glucosides, and benzoic acid ester *N*-glucoside have not been described to date. CtMaT1 from *Cistanche tubulosa* is also capable of acylating various types of glycosides.^52^ The converted acyl acceptors are natural products useful for pharmaceutical and nutraceutical applications, such as β-glucogallin due to its antidiabetic activity,^4^ apigenin-7-glucoside due to its anti-inflammatory activity,^53^ fraxetin-8-glucoside (Fraxin) due to its hepatoprotective activity,^54^ and thymol glucoside due to its expectorant and antibacterial activity.^55^

FaMATs malonylated a range of glycosides with different glycone types, showing a clear preference for glucosides. Additionally, three galactosides, such as isorhamnetin-3-*O*-galactoside **13**, quercetin-3-*O*-galactoside **20**, and myricetin-3-*O*-galactoside **21** were also malonylated. Compared to product amounts formed from isorhamnetin-3-*O*-glucoside **10** and quercetin-3-*O*-glucoside **12**, the amount of malonylated galactosides were lower. FaMATs also converted xylosides such as quercetin-3-*O*-xyloside **30** and 4-methylumbelliferyl-*O*-xyloside **43**. However, the corresponding product amounts were lower compared to the galactosides and glucosides carrying the same aglycones. FaMAT4C_1_/S_1_ did not form a product of quercetin-3-*O*-xyloside **30**.

*O-*glycosides as well as *C*- (apigenin-6-*C*-glucoside **1**, luteolin-6-*C*-glucoside **2**, apigenin-8-*C*-glucoside **5**, and mangiferin **54**), and *N*-glycosides (methyl anthranilate *N*-glucoside **67**) were malonylated. The glycones could be bound to different sites of the aglycone. For flavonoid glycosides, the sugar could be linked at position C5 (**6**), C6 (**1**, **2**), C7 (**3-4**, **7**, **19**, **22**-**29**, **31**-**35**), C8 (**5**, **8**) on ring A, position C4 (**14**, **15**) on ring B, and position C3 (**9**, **10**, **11**-**13**, **15**-**18**, **20**-**23**, **30**, **36**-**40**) on ring C. FaMAT4C_1_/S_1_ formed the lowest amounts of malonylated *C*-glucosides, and no product was obtained with apigenin-8-*C*-glucoside **5**. FaMAT1C and FaMAT1S, however, showed regiospecificity as a larger amount of product was formed with 6-*C*-glucoside **1** compared to 8-*C*-glucoside **5**.

FaMATs converted not only monoglycosides but also diglycosides, such as compounds with two glucosyl groups (**15**, **22**, **23**, **66**), glucosyl and rhamnosyl (**17**, **18**), as well as glucosyl and rutinosyl (**31**). FaMAT4C_1_/S_1_ formed three isomeric monomalonylated products from quercetin-3,4-di-*O*-glucoside **15** (Figure S10) and kaempferol-3,7-di-*O*-glucoside **22** (Figure S13), while the other FaMATs produced only one or two isomers from the diglucosides, respectively. All FaMATs malonylated quercetin-3,7-di-*O*-glucoside **23** to three isomeric monomalonylated products (Figure S13). In contrast, all proteins only produced a monomalonylated product of kaempferol-3-*O*-glucoside-7-*O*-rhamnoside **17** and quercetin-3-*O*-glucoside-7-*O*-rhamnoside **18** (Figure S11). Notably, FaMAT4C_1_/S_1_ and FaMAT1S converted kaempferol-7-*O*-glucoside-3-*O*-rutinoside **31** into two and one monomalonylated products (*m*/*z* 843 [M + H]^+^), respectively. Their pseudomolecular ions were fragmented into kaempferol-7-malonylglucoside with *m*/*z* 535 [M + H]^+^ (Figure S16). In addition, a monomalonylated product of secoisolariciresinol diglucoside **66** (*m*/*z* 771 [M-H]^−^) was formed only in extremely small amounts (Figure S26).

FaMATs exhibited dimalonylation activity using diglucosides, such as quercetin-3,4-*O*-diglucoside **15** (Figure S10), kaempferol-3,7-*O*-diglucoside **22** (Figure S13), and quercetin-3,7-*O*-diglucoside **23** (Figure S13). FaMAT4C_1_/S_1_ converted quercetin-3,4-*O*-diglucoside **15** into a dimalonylated product (*m*/*z* 797 [M-H]^−^). However, the product yield was very low. All FaMATs produced dimalonylated products of kaempferol-3,7-*O*-diglucoside **22** (*m*/*z* 781 [M-H]^−^) and quercetin-3,7-*O*-diglucoside **23** (*m*/*z* 797 [M-H]^−^). FaMAT1S formed the highest amount of dimalonylated products. Other BAHD/HXXXD acyltransferases also exhibit dimalonylation activity, such as CtMaT1 from *C. tubulosa* ^52^ and Aat1 anthocyanin acyltransferase from maize.^56^

FaMAT1C had the lowest acylation activity among the FaMAT proteins. Moreover, some substrates could not be acylated by FaMAT1C, such as tricetin-7-*O*-glucoside **7**, kaempferol-7-glucoside-3-*O*-rutinoside **31**, petunidin-3-*O*-glucoside **40**, and β-glucogallin **53**. In contrast, FaMAT4C_1_/S_1_ showed the highest product yields for most substrates. FaMAT1S and FaMAT1C showed a similar product spectrum, which confirms the assumption that they are orthologs (Figure 3). The specific activity of FaMATs was determined based on the initial rate, with the enzymatic assay conducted at pH 7.0 and 30 °C for 30 min (Figure S27). FaMAT4C_1_/S_1_ exhibited high activity on most substrates, including apigenin-7-*O*-glucoside **3**, pelargonidin-3-*O*-glucoside **36**, thymol *O*-glucoside **49**, 5-EHMF *O*-glucoside **55**, HDMF *O*-glucoside **56**, sotolon *O*-glucoside **57**, and maple furanone *O*-glucoside **58**, while it showed very low activity on apigenin-6-*C*-glucoside **1**. FaMAT1S demonstrated the highest activity on apigenin-6-*C*-glucoside **1**, quercetin-3-*O*-glucoside **12**, and aesculin **41**. Both FaMAT1S and FaMAT1C showed high activity toward apigenin-6-*C*-glucoside **1** and low activity toward apigenin-7-*O*-glucoside **3**, suggesting a preference for *C*-glucosides over *O*-glucosides. In contrast, FaMAT4C1/S1 displayed the opposite selectivity.

Despite their broad substrate promiscuity, the FaMATs were unable to acylate 20 glycosides (Table S4). FaMATs could not esterify rhamnosides, arabinosides, rhamnoglucosides, arabinoglucosides, and rutinosides. FaMATs preferably catalyzed the acylation of glucosides and galactosides that possess a 6-OH group, with the exception of xylosides, which are also acylated to a lesser extent. In addition, FaMATs are unable to malonylate some *C*-glucosides and a sophoroside, such as apigenin-6-*C*-glucoside-7-*O*-glucoside, apigenin-6,8-di-*C*-glucoside, luteolin-6,8-di-*C*-glucoside, apigenin-6-*C*-glucoside-8-*C*-arabinoside, and kaempferol-3-*O*-sophoroside-7-*O*-glucoside, possibly due to steric hindrance.

We further analyzed the acyl donor preference of the FaMATs. In this screening, aliphatic acyl donors, such as malonyl-CoA, acetyl-CoA, acryloyl-CoA, and crotonyl-CoA, as well as aromatic acyl donors, such as p-coumaroyl-CoA, feruloyl-CoA, and 4-hydroxybenzoyl-CoA were used. FaMATs used only malonyl-CoA as an acyl donor. The FaMATS analyzed in this study are therefore acyl donor-specific transferases that only transfer malonic acid but have a broad substrate acceptance with regard to their acceptor molecules.

To identify the binding sites of FaMATs for malonyl-CoA and the acyl acceptor HDMF glucoside, protein structures were predicted using AlphaFold2, and molecular docking was performed using AutoDock Vina (Supporting Data 1; Figures S36–S39). The docking results revealed that a conserved histidine residue within the HXXXD motif could play a critical role in substrate binding. This residue is probably involved in interactions with both malonyl-CoA and HDMF glucoside, highlighting its importance in the catalytic mechanism of FaMATs (Figures S38 and S39).

### Malonylated Glucosides Are Protected from Glucosidase Attack

Given previous reports suggesting that malonylated glucosides exhibited increased resistance to glycosidase-mediated hydrolysis,^57^ we conducted a detailed investigation into the enzymatic stability of malonyl glycosides. This analysis aimed to evaluate the potential biological significance of biochemical acylation in protecting glycosides from degradation. Quercetin-3-*O*-glucoside, pelargonidin-3-*O*-glucoside, HDMF glucoside, scopolin, and quercetin-3-*O*-galactoside were malonylated using FaMAT4C_1_/S_1_ and kaempferol-3,7-*O*-diglucoside using FaMAT1S. The mixtures of products and remaining substrates were subjected to hydrolysis by almond β-glucosidase. As a control, the corresponding glycosides were incubated with identical hydrolase. LC-MS analyses revealed that in the mixtures of malonylated product and glucoside substrates, the glucosides are readily degraded by glucosidase, whereas quercetin-3-*O*-(6′-*O*-malonyl)-glucoside, pelargonidin-3-*O*-(6′-*O*-malonyl)-glucoside, HDMF (6′-*O*-malonyl) glucoside, and malonylated scopolin resist enzymatic attack (Figures S28 and S29).

Quercetin-3-*O*-galactoside was not degraded by almond β-glucosidase because the enzyme is specific for the hydrolysis of β-glucosides (Figure S30). The dimalonylated product of kaempferol-3,7-di-*O*-glucoside (*m*/*z* 781 [M-H]^−^) was also stable against enzymatic hydrolysis (Figure 6). While the monomalonylated products (*m*/*z* 695 [M-H]^−^) were degraded to kaempferol-3-*O*-(6′-*O*-malonyl)-glucoside (*m*/*z* 533 [M-H]^−^) and kaempferol-7-*O*-(6′-*O*-malonyl)-glucoside (*m*/*z* 533 [M-H]^−^), the dimalonylated diglucoside (*m*/*z* 781 [M-H]^−^) remained untouched. Kaempferol-3-*O*-glucoside (*m*/*z* 447 [M-H]^−^), kaempferol-7-*O*-glucoside (*m*/*z* 447 [M-H]^−^), and kaempferol (*m*/*z* 285 [M-H]^−^) were formed by hydrolysis of the diglucoside substrate in the mixture (Figure 6). This study shows that malonylation effectively protects glucosides from hydrolysis by β-glucosidase. Steric hindrance of the aliphatic acyl groups protects the glucosidic bond from nucleophilic attack.^58^

**Figure 6:**
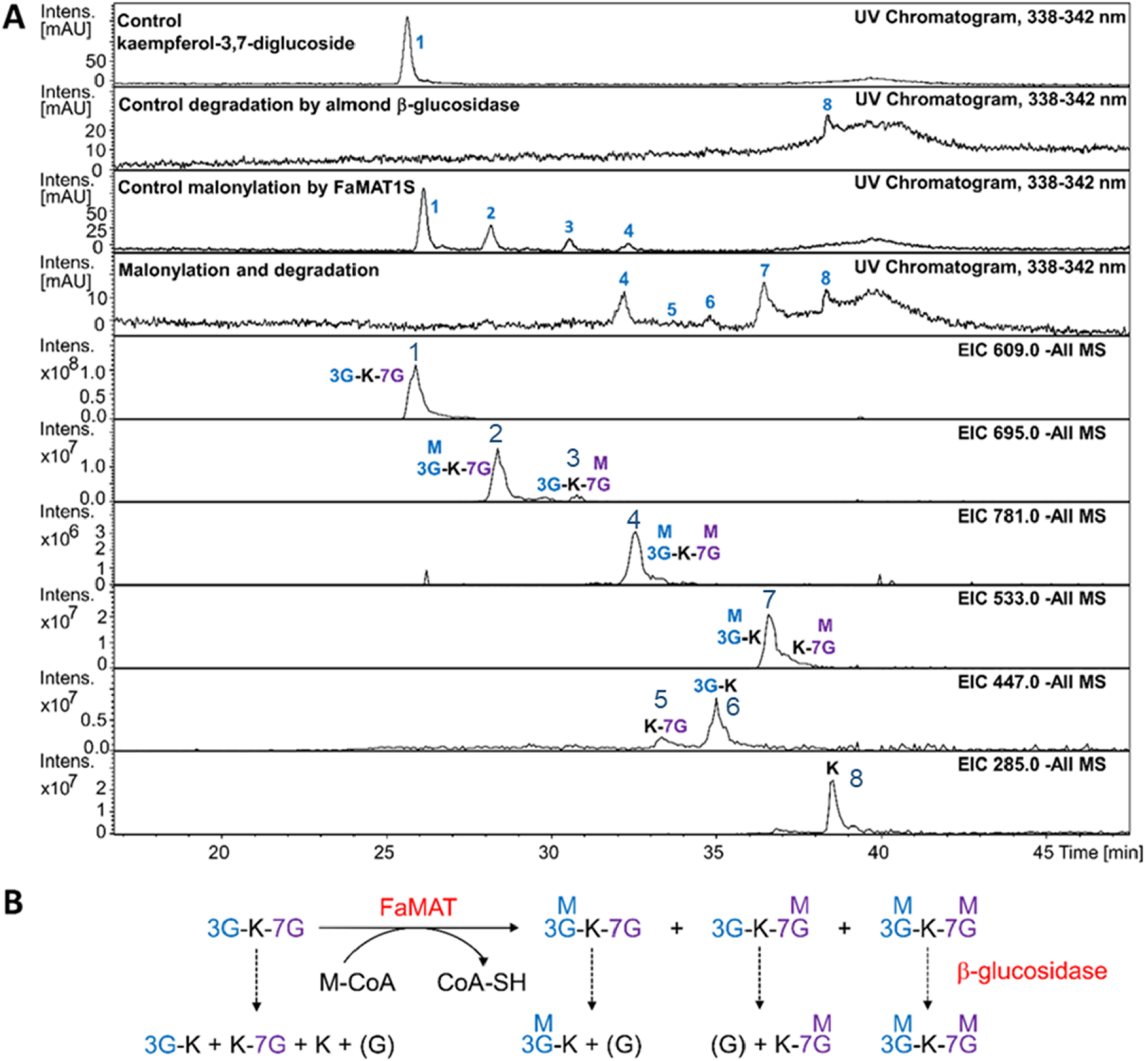
LC-MS analysis to investigate the stability of malonylated products formed from kaempferol-3,7-di-*O*-glucoside toward almond β-glucosidase. LC-MS chromatograms (**A**). Compound **1**: kaempferol-3,7-di-*O*-glucoside *m*/*z* 609 [M-H]^−^, **2** and **3**: monomalonylated product of kaempferol-3,7-di-*O*-glucoside *m*/*z* 695 [M-H]^−^, **4**: dimalonylated product of kaempferol-3,7-di-*O*-glucoside *m*/*z* 781 [M-H]^−^, **5**: kaempferol-7-*O*-glucoside *m*/*z* 447 [M-H]^−^, **6**: kaempferol-3-*O*-glucoside *m*/*z* 447 [M-H]^−^, **7**: kaempferol-3-*O*-(6′-*O*-malonyl)-glucoside and kaempferol-7-*O*-(6′-*O*-malonyl)-glucoside *m*/*z* 533 [M-H]^−^, **8**: kaempferol *m*/*z* 285 [M-H]^−^. Scheme for explaining product formation (**B**). K, kaempferol; G, glucose; M, malonic acid. FaMAT forms three malonylated products from kaempferol-3,7-di-*O*-glucoside, whereas glucosidase can only cleave glucosides but is unable to release malonylated glucose.

### Maple Furanone Malonyl Glucoside Was Identified

To confirm the binding site of malonic acid, we isolated and identified the previously undescribed malonylated maple furanone glucosides. FaMAT4C_1_/S_1_ produced three isomers of maple furanone malonyl glucoside (Figure S31). The two major isomers were isolated using semipreparative HPLC. We detected the substrate (maple furanone glucoside) in the product fractions, likely due to the degradation of the malonylated products under acidic conditions. The solvent used (5% ethanol with 0.1% formic acid) had a pH of 3.04, which may have promoted the hydrolysis of the malonyl group. We therefore carried out a second separation using the same column but a buffered mobile phase (ethanol 2% with 10 mM ammonium formate at pH 6.45). Unlike the first separation, malonylated products elute earlier, as they are ionized at high pH and more polar than the substrate maple furanone glucoside (Figure S31B–C).

Isolated maple furanone malonyl glucosides were identified by ^1^H NMR, 2D DQC–COSY, H,C HSQC, and H,C HMBC (Figures S32–S35). The ^1^H NMR data of maple furanone malonyl glucoside isomer 1 and isomer 2 are shown in Table S5. There is a downfield shift in position 6-OH on the glucosyl moiety of the product isomer 1 (δ 4.21 and δ 4.02) and isomer 2 (δ 4.25 and δ 4.04) compared to that of maple furanone glucoside (δ 3.63 and δ 3.45). The ^13^C NMR analysis shows a chemical downfield shift for C-6′ of the glucosyl group of product isomer 1 (δ 63.98) and product isomer 2 (δ 63.90) compared to that of the substrate (δ 61.16) (Table S6). NMR identification demonstrates that the malonyl groups of both product isomers were transferred to the same position 6′–OH of the glucosyl moiety (Figure 7). The chemical shifts of ^1^H and ^13^C are almost identical for both isomers at all positions. It is therefore assumed that two isomers are the 6′–OH malonylated diastereomeric β-d-glucosides of (*R*)-(+)-maple furanone and (*S*)-(−)-maple furanone.^47^

**Figure 7:**
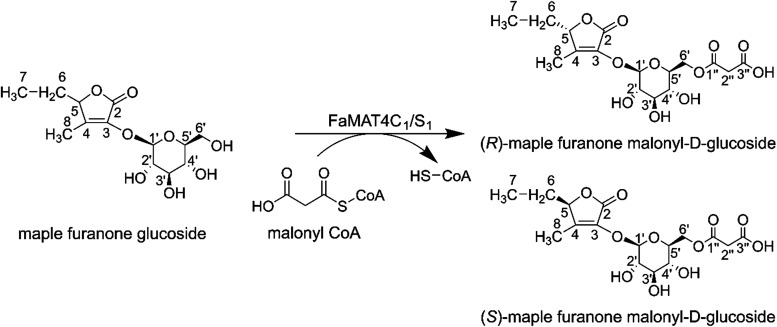
Malonylation of maple furanone glucoside by FaMAT4C_1_/S_1_.

## Supplementary Material



## Notes

Primer sequence for gene cloning (Table S1); gene assignment on NCBI (Table S2); list of acceptors that are malonylated by FaMATs (Table S3); list of acceptors that could not be acylated by the FaMATs (Table S4); ^1^H NMR and ^13^C NMR of maple furanone malonyl glucoside isomer 1 and isomer 2 (Tables S5 and S6); multiple alignments of FaMAT proteins (Figure S1); extracted ion chromatogram and mass spectrum of malonylated products from 5-EHMF-glucoside, sotolon glucoside, and maple furanone glucoside as well as the enantiomers of 5-EHMF, sotolon, and maple furanone (Figures S2 and S3); effect of pH, temperature, and incubation time on FaMAT enzymatic activity (Figure S4); Michaelis–Menten plots of FaMAT activity toward furanone glucosides (Figure S5); UV chromatogram and extracted ion chromatogram of glycosides and malonylated products, as well as the mass spectra of the malonylated products (Figures S6–S26); specific activity of FaMATs (Figure S27); stability of malonylated products from quercetin-3-*O*-glucoside, pelargonidin-3-*O*-glucoside, HDMF glucoside, scopolin, and quercetin-3-*O*-galactoside against almond β-glucosidase (Figures S28–S30); UV chromatogram of separation of malonylated maple furanone glucoside (Figure S31); ^1^H NMR spectrum, COSY, HSQC, and HMBC of maple furanone malonyl glucoside isomer 1 and isomer 2 (Figures S32–S35); molecular docking predicted the binding sites of FaMATs and malonyl-CoA and HDMF glucoside (Supplementary Data 1); Ramachandran plot of the FaMAT protein model (Figure S36); prediction of secondary structures of FaMATs (Figure S37); and docking of FaMATs with malonyl-CoA and HDMF glucoside (Figures S38 and S39) (PDF)

## Abbreviations and Nomenclature

HDMF: 4-hydroxy-2,5-dimethyl-3­(2*H*)-furanone
FaMAT: *Fragaria* × *ananassa* malonyltransferase
CoA: coenzyme A
PCR: polymerase chain reaction
LB: lysogeny broth
GST: glutathione-*S*-transferase
IPTG: isopropyl β-d-thiogalactopyranoside
PMSF: phenylmethylsulfonyl fluoride
SDS PAGE: sodium dodecyl-sulfate polyacrylamide gel electrophoresis
EDTA: ethylenediaminetetraacetic acid
LC-ESI-MS: liquid chromatography–electrospray ionization–mass spectrometry
HPLC: high-performance liquid chromatography
UV: ultraviolet
DAD: diode array detector
EIC: extracted ion chromatogram
5-EHMF: 5-ethyl-4-hydroxy-2-methyl-3­(2*H*)-furanone
NMR: nuclear magnetic resonance
HMMF: 4-hydroxy-5-methyl-2-methylene-3­(2*H*)-furanone
FaEO: *Fragaria* × *ananassa* enone oxidoreductase
DMMF: 2,5-dimethyl-4-methoxy-3­(2*H*)-furanone
FaOMT: *Fragaria* × *ananassa* O-methyltransferase
FaGT: *Fragaria* × *ananassa* glucosyltransferase
COSY: correlated spectroscopy
HSQC: heteronuclear single-quantum coherence
HMBC: heteronuclear multiple-bond correlation
