# Supplementary material for: Promiscuous Transferases Malonylate Furaneol Glucoside in Fragaria × ananassa
Source: J Agric Food Chem. 2026 Feb 5;74(6):5442–53. doi: 10.1021/acs.jafc.5c09017 (PMC12921856; doi:10.1021/acs.jafc.5c09017)
Supplement: Supplementary file 1 [file jf5c09017_si_001.pdf]

Supporting Information

Promiscuous transferases malonylate furaneol glucoside in *Fragaria* × *ananassa*

Martha Purnami Wulanjati[1,2], Johanna Trinkl[1], Xiran Wang[1], Thomas Hoffmann[1], Wilfried Schwab[1]*

[1]Biotechnology of Natural Products, TUM School of Life Sciences, Technical University of Munich, 85354 Freising, Germany

[2]Research Center for Food Technology and Processing (PRTPP), National Research and Innovation Agency (BRIN), 55861 Yogyakarta, Indonesia

*Corresponding author:

Wilfried Schwab

wilfried.schwab@tum.de

Tables

Table S1. Primer sequences for gene cloning

**Gene amplification from genomic DNA**		
1_Gene04261_f_untr	5‘-CTGGTTCAATCTCTAGTCTGTCTCTCCA-3‘	Metabion, Planegg
1_Gene04261_r_untr	5‘-GCAACAACATTAACACCATTATTATTGCT-3‘	Metabion, Planegg
1_Gene04261_f_ATG	5‘-ATGGCACATCCAAACTCACTTGTAAATG-3‘	Metabion, Planegg
1_Gene04261_r_ATG	5‘-TCAAAGGTTTTCAAGACCTTTGGCAAATTGTG-3‘	Metabion, Planegg
2_Gene04262_f_untr	5‘-CATTTTCTATCTCTTTGCTCCTTCGTAAGTA-3‘	Metabion, Planegg
2_Gene04262_r_untr	5‘-GTTTCTCTTGGTCCATAATCTCAATC-3‘	Metabion, Planegg
2_Gene04262_f_ATG	5‘-ATGGCAAACTTATCAGTGAAGAAAGTTGAGG-3‘	Metabion, Planegg
2_Gene04262_r_ATG	5‘-CTAATGCACTAGGCTAGCAAAGAGAGCA-3‘	Metabion, Planegg
3_Gene03835_f_untr	5‘-AGTTTCAATCAATTCAGCATCATCTCCA-3‘	Metabion, Planegg
3_Gene03835_r_untr	5‘-ATTGGGCCTTGGTGTACATATGGCT-3‘	Metabion, Planegg
3_Gene03835_f_ATG	5‘-ATGGCATCTCCAAACTCATCTGCAAGAG-3‘	Metabion, Planegg
3_Gene03835_r_ATG	5‘-TCAATTTCCCTTGAGACCTTCGGCAAATAG-3‘	Metabion, Planegg
4_Gene29347_f_untr	5‘-CTTCTTCTAATCTTCTTCTTCCCCA-3‘	Metabion, Planegg
4_Gene29347_r_untr	5‘-ATAGGGCTTTTCTCAACTCCTCTCAC-3‘	Metabion, Planegg
4_Gene29347_f_ATG	5‘-ATGGAGCAACCAAGCTCGGTGAAACTGG-3‘	Metabion, Planegg
4_Gene29347_r_ATG	5‘-TCAGTGTTTTCCAAAACCTTTAGCAAATAGAG-3‘	Metabion, Planegg
5_Gene04266_f_untr	5‘-CAAGTTTCTTCCAATTGATCATCTCTA-3‘	Metabion, Planegg
5_Gene04266_f_ATG	5‘-ATGGCTTTCCGAAACTCAACTACAAAAGTGG-3‘	Metabion, Planegg
5_Gene04266_r_ATG	5‘-TCAAGCTGGAGCCGATACCTCCCAGGCGGTTTTT GACCTCGCG-3‘	Metabion, Planegg
**Subcloning into pGEX4T-1**		
C1-f	5'-ATGGATCCATGGCACATCCAAACTCACTTG-3'	Metabion, Planegg
C1-r	5'-ATGCGGCCGCTCAAAGGTTTTCAAGGCCTTTG-3'	Metabion, Planegg
C2-f	5'-ATGGATCCATGGCAAACTTATCAGTGAAGAAAG-3'	Metabion, Planegg
C2-r	5'-ATGCGGCCGCCTAATGCACTAGGCTAGCAAAG-3'	Metabion, Planegg
C3-f	5'-ATGGATCCATGGCATCTCCAAACTCATCTG-3'	Metabion, Planegg
C3-r	5'-ATGCGGCCGCTCAGCCTTGACTAAACAAACCC-3'	Metabion, Planegg
C4-f	5'-ATGGATCCATGGAGCAACCAAGCTCGG-3'	Metabion, Planegg
C4-r	5'-ATGCGGCCGCTCAGTGTTTTCCAAAACCTTTAG-3'	Metabion, Planegg
S1-f	5'-ATGGATCCATGGCACATCCAAACTCACTTG-3'	Metabion, Planegg
S1-r	5'-ATGCGGCCGCTCAAAGGTTTTCAAGGCCTTTG-3'	Metabion, Planegg
C5-f	5'-ATGGATCCATGGCTTTCCGAAACTCAACTAC-3'	Metabion, Planegg
C5-r	5'-ATGCGGCCGCTCAAATCCTAGAATGGTTTTTGAG-3'	Metabion, Planegg

The underlined sequence part indicates the cleavage sites of restriction enzymes.

Table S2. Gene assignment on NCBI, National Center for Biotechnology Information (www.ncbi.nlm.nih.gov)

No.	Code of gene	Accession number
1	MAT1C	OR636097 (Candonga)
2	MAT1S	OR636099 (Senga sengana)
3	MAT4C$_1$/S$_1$	OR636098 (Candonga)

Table S3. List of acceptors that are malonylated by FaMATs

Compound group	No.	Compound name	Substrate RT (min)	Substrate formula	Product RT (min)	Product formula	positive MS (*m/z*)	negative MS (*m/z*)	positive MS2 (*m/z*)	negative MS2 (*m/z*)
Flavone glycoside	1	Apigenin-6-*C*-glucoside/ Isovitexin	31.3	$C_{21}H_{20}O_{10}$	32.8; 34.0	$C_{24}H_{22}O_{13}$	519 $[M+H]^+$	517 $[M-H]^-$	501, 483, 465, 433, 415, 379, 361, 337, 313	473, 311
	2	Luteolin-6-*C*-glucoside/ Homoorrientin	29.3	$C_{21}H_{20}O_{11}$	31.3; 32.3	$C_{24}H_{22}O_{14}$	535 $[M+H]^+$	533 $[M-H]^-$	517, 499, 481, 449, 431, 395, 377, 353, 329, 299, 287	489, 327
	3	Apigenin-7-*O*-glucoside	33.7	$C_{21}H_{20}O_{10}$	36.1; 36.9	$C_{24}H_{22}O_{13}$	519 $[M+H]^+$	473 $[M-COOH]^-$	475, 433, 271	413, 377, 311, 293, 269
	4	Luteolin-7-*O*-glucoside	31.7	$C_{21}H_{20}O_{11}$	34.5; 35.6	$C_{24}H_{22}O_{14}$	535 $[M+H]^+$	533 $[M-H]^-$	449; 287, 241	489
	5	Apigenin-8-*C*-glucoside	29.6	$C_{21}H_{20}O_{10}$	32.4	$C_{24}H_{22}O_{13}$	519 $[M+H]^+$	517 $[M-H]^-$	501, 475, 457, 439, 415, 379, 361, 337, 313, 295, 271	473, 311
	6	Tectochrysin-5-*O*-glucoside	38.2	$C_{22}H_{22}O_{9}$	38.6	$C_{5}H_{24}O_{12}$	517 $[M+H]^+$	471 $[M-COOH]^-$	269	267
	7	Tricetin-7-*O*-glucoside	29.4	$C_{21}H_{20}O_{12}$	33.6	$C_{24}H_{22}O_{15}$	551 $[M+H]^+$	549 $[M-H]^-$	465, 303	505
Flavonol glycoside	8	Herbacetin-8-*O*-glucoside	36.0	$C_{21}H_{20}O_{12}$	37.0	$C_{24}H_{22}O_{15}$	551 $[M+H]^+$	505 $[M-COOH]^-$	303	445, 301
	9	Methylmyricetin-3-*O*-glucoside	32.5	$C_{22}H_{22}O_{13}$	34.2	$C_{25}H_{24}O_{16}$	581 $[M+H]^+$	579 $[M-H]^-$	375, 333	535, 331
	10	Isorhamnetin-3-*O*-glucoside	35.1	$C_{22}H_{22}O_{12}$	36.7	$C_{25}H_{24}O_{15}$	565 $[M+H]^+$	563 $[M-H]^-$	317	315, 300

11	Patuletin-3-*O*-glucoside	32.5	$C_{22}H_{22}O_{13}$	33.6	$C_{25}H_{24}O_{16}$	581 $[M+H]^+$	535 $[M-COOH]^-$	333	493, 475, 331, 287
12	Quercetin-3-*O*-glucoside	32.7	$C_{21}H_{20}O_{12}$	34.0	$C_{24}H_{21}O_{15}$	551 $[M+H]^+$	549 $[M-H]^-$	303	505, 301
13	Isorhamnetin 3-*O*-galactoside/ Cacticin	34.9	$C_{22}H_{22}O_{12}$	36.6	$C_{25}H_{24}O_{15}$	565 $[M+H]^+$	563 $[M-H]^-$	317	519, 315
14	Quercetin-4-*O*-glucoside/ Spiraeosid	34.4	$C_{21}H_{20}O_{12}$	36.9	$C_{24}H_{22}O_{15}$	551 $[M+H]^+$	549 $[M-H]^-$	303	505, 301
15	Quercetin-3,4-*O*-diglucoside	28.7	$C_{27}H_{30}O_{17}$	30.3; 31.3; 32.3 (mono-malonyl)	$C_{30}H_{32}O_{20}$	713 $[M+H]^+$	711 $[M-H]^-$	551, 465, 345, 303	667, 505, 463, 301
				33.7 (dimalonyl)	$C_{33}H_{34}O_{23}$		797 $[M-H]^-$		709, 505, 301
16	Kaempferol-3-*O*-glucoside	34.7	$C_{21}H_{20}O_{11}$	36.3	$C_{24}H_{22}O_{14}$	535 $[M+H]^+$	533 $[M-H]^-$	287	489
17	Kaempferol-3-*O*-glucoside-7-*O*-rhamnoside	32.1	$C_{27}H_{30}O_{15}$	33.7	$C_{30}H_{32}O_{18}$	681 $[M+H]^+$	679 $[M-H]^-$	535, 433, 287	635, 489
18	Quercetin-3-*O*-glucoside-7-*O*-rhamnoside	30.0	$C_{27}H_{30}O_{16}$	31.3	$C_{30}H_{32}O_{19}$	697 $[M+H]^+$	695 $[M-H]^-$	551, 533, 449, 303	651
19	Quercetagetin-7-*O*-glucoside	28.2	$C_{21}H_{19}O_{13}$	31.8	$C_{24}H_{21}O_{16}$	567 $[M+H]^+$	565 $[M-H]^-$	319	521, 317
20	Quercetin-3-*O*-galactoside	32.0	$C_{21}H_{20}O_{12}$	33.9	$C_{24}H_{22}O_{15}$	551 $[M+H]^+$	549 $[M-H]^-$	303	505, 301
21	Myricetin-3-*O*-galactoside	30.0	$C_{21}H_{20}O_{13}$	32.1	$C_{24}H_{22}O_{16}$	567 $[M+H]^+$	565 $[M-H]^-$	319	521
22	Kaempferol-3,7-*O*-diglucoside	26.0	$C_{27}H_{30}O_{16}$	28.0; 29.2; 30.3 (mono-malonyl)	$C_{30}H_{32}O_{19}$	697 $[M+H]^+$	695 $[M-H]^-$	535, 517, 449, 287	651, 531, 489
				32.0 (dimalonyl)	$C_{33}H_{34}O_{22}$		781 $[M-H]^-$		693, 531, 489

	23	Quercetin-3,7-*O*-diglucoside	24.0	$C_{27}H_{30}O_{17}$	25.7; 27.3; 28.6 (mono-malonyl)	$C_{30}H_{32}O_{20}$	713 $[M+H]^+$	711 $[M-H]^-$	551, 465, 345, 303	667
					30.0 (dimalonyl)	$C_{33}H_{34}O_{23}$		797 $[M-H]^-$		753, 709
	24	Kaempferol-7-*O*-glucoside	32.8	$C_{21}H_{20}O_{11}$	35.6; 36.5	$C_{24}H_{22}O_{14}$	535 $[M+H]^+$	489 $[M-COOH]^-$	449, 287	429, 327, 285
	25	6-Hydroxy-kaempferol-7-*O*-glucoside	30.9	$C_{21}H_{20}O_{12}$	34.3	$C_{24}H_{22}O_{15}$	551 $[M+H]^+$	505 $[M-COOH]^-$	303	301
	26	Patuletin-7-*O*-glucoside	32.5	$C_{22}H_{22}O_{13}$	33.6	$C_{25}H_{24}O_{16}$	581 $[M+H]^+$	535 $[M-COOH]^-$	333	475, 373, 331, 287
	27	Quercetin-7-*O*-glucoside	30.2	$C_{21}H_{20}O_{12}$	32.9; 34.0	$C_{24}H_{22}O_{15}$	551 $[M+H]^+$	549 $[M-H]^-$	465, 303	505
	28	Tamarixetin-7-*O*-glucoside	33.9	$C_{22}H_{22}O_{12}$	36.8	$C_{25}H_{24}O_{15}$	565 $[M+H]^+$	563 $[M-H]^-$	479, 317	519, 357, 315
	29	6-Methoxy-kaempferol-7-*O*-glucoside	33.9	$C_{22}H_{22}O_{12}$	36.7	$C_{25}H_{24}O_{15}$	565 $[M+H]^+$		317	
	30	Quercetin-3-*O*-xyloside	33.1	$C_{20}H_{18}O_{11}$	35.6	$C_{23}H_{20}O_{14}$	521 $[M+H]^+$	475 $[M-COOH]^-$	303	415, 301, 271
	31	Kaempferol-7-*O*-glucoside-3-*O*-rutinoside	26.5	$C_{33}H_{40}O_{20}$	29.4; 30.9	$C_{36}H_{42}O_{23}$	843 $[M+H]^+$	841 $[M-H]^-$	681, 535, 287	797, 593
Flavanone glycoside	32	Eriodictyol-7-*O*-glucoside	27.9	$C_{21}H_{22}O_{11}$	30.9; 32.1	$C_{24}H_{24}O_{14}$	537 $[M+H]^+$	535 $[M-H]^-$	519, 501, 471, 397, 365, 331, 289, 253, 163	491, 287
	33	Naringenin-7-*O*-glucoside	30.3	$C_{21}H_{22}O_{10}$	33.1; 34.3	$C_{24}H_{24}O_{13}$	521 $[M+H]^+$	519 $[M-H]^-$	503, 485, 381, 315, 297, 273, 179, 153	475, 271
Isoflavone glycoside	34	Genistein-7-*O*-glucoside/ Genistin	30.0	$C_{21}H_{20}O_{10}$	32.9; 34.9	$C_{24}H_{22}O_{13}$	519 $[M+H]^+$		433, 271	

	35	Irigenin-7-*O*-glucoside	32.1	$C_{24}H_{26}O_{13}$	34.2; 36.2	$C_{27}H_{28}O_{16}$	609 $[M+H]^+$		361	
Antho-cyanin glycoside	36	Pelargonidin-3-*O*-glucoside	23.4	$C_{21}H_{21}O_{10}^+$	26.5; 28.4	$C_{24}H_{23}O_{13}^+$	519 $[M]^+$		433, 271	
	37	Delphinidin-3-*O*-glucoside	22.0	$C_{21}H_{21}O_{12}^+$	26.9	$C_{24}H_{23}O_{15}^+$	551 $[M]^+$		303	
	38	Cyanidin-3-*O*-glucoside	22.0	$C_{21}H_{21}O_{11}^+$	25.3; 27.2	$C_{24}H_{23}O_{14}^+$	535 $[M]^+$		449, 287	
	39	Peonidin-3-*O*-glucoside	24.2	$C_{22}H_{23}O_{11}^+$	28.9	$C_{25}H_{25}O_{14}^+$	549 $[M]^+$		301	
	40	Petunidin-3-*O*-glucoside	22.8	$C_{22}H_{23}O_{12}^+$	27.7	$C_{25}H_{25}O_{15}^+$	565 $[M]^+$		521, 317	
Coumarin glycoside	41	6,7-dihydroxy-coumarin-6-*O*-glucoside/ Aesculin	18.5	$C_{15}H_{16}O_9$	23.6; 25.5	$C_{18}H_{18}O_{12}$	427 $[M+H]^+$	381 $[M-COOH]^-$	179	177
	42	4-methyl-umbelliferyl-D-*O*-glucoside	22.2	$C_{16}H_{18}O_8$	26.3; 28.4	$C_{19}H_{20}O_{11}$	425 $[M+H]^+$		177	
	43	4-methyl-umbelliferyl-D-*O*-xyloside	26.8	$C_{15}H_{16}O_7$	31.4	$C_{18}H_{18}O_{10}$	395 $[M+H]^+$		177	
	44	Fraxetin-8-*O*-glucoside/ Fraxin	21.7	$C_{16}H_{18}O_{10}$	23.7; 25.1	$C_{19}H_{20}O_{13}$	457 $[M+H]^+$	455 $[M-H]^-$	421, 209	411, 369, 207
	45	7-hydroxy-6-methoxycoumarin-7-*O*-glucoside/ Scopolin	19.8	$C_{16}H_{18}O_9$	23.6; 25.8	$C_{19}H_{20}O_{12}$	441 $[M+H]^+$		193	
Phenolic glycoside	46	Vanillin *O*-glucoside	17	$C_{14}H_{18}O_8$	24.5	$C_{17}H_{20}O_{11}$	423 $[M+Na]^+$		379, 361, 337, 259, 227, 175	
	47	Sesamol *O*-glucoside	16.5	$C_{13}H_{16}O_8$	23.5; 24.5	$C_{16}H_{18}O_{11}$	409 $[M+Na]^+$		365, 323, 305, 271, 229, 167	
	48	Raspberry ketone *O*-glucoside	20.2	$C_{16}H_{22}O_7$	26.0	$C_{19}H_{24}O_{10}$	435 $[M+Na]^+$		391, 349, 187	

	49	Thymol *O*-glucoside	37.4	$C_{16}H_{24}O_6$	38.3	$C_{19}H_{26}O_9$	421 [M+Na]$^+$		377, 335, 271, 218	
	50	Arbutin	6.6	$C_{12}H_{16}O_7$	15.1	$C_{15}H_{18}O_{10}$	381 [M+Na]$^+$	313 [M-COOH]$^-$	337, 295, 272, 253, 227	253, 235, 203, 151, 109
	51	Eugenol *O*-glucoside	32.4	$C_{16}H_{22}O_7$	35.8	$C_{19}H_{24}O_{10}$	435 [M+Na]$^+$		391, 373, 349, 227	
	52	Carvacrol *O*-glucoside	37.2	$C_{16}H_{24}O_6$	38.1	$C_{19}H_{26}O_9$	421 [M+Na]$^+$		377, 359, 335, 271, 127	
	53	1-*O*-galloyl-β -D-glucopyranoside/ β-glucogallin	8.9	$C_{13}H_{16}O_{10}$	14.8; 15.7	$C_{16}H_{18}O_{13}$		373 [M-COOH]$^-$		313, 240, 211, 193, 169, 151, 125
Anthra-quinone glycoside	54	Mangiferin	26.4	$C_{19}H_{18}O_{11}$	30.4	$C_{22}H_{20}O_{14}$	509 [M+H]$^+$	507 [M-H]$^-$	491, 473, 423, 405, 369, 351, 327, 303, 271, 229	463, 421, 356, 331, 301
Furanone glycoside	55	5-EHMF *O*-glucoside	19.8	$C_{13}H_{20}O_8$	24.6; 25.4; 25.9	$C_{16}H_{22}O_{11}$	391 [M+H]$^+$	345 [M-COOH]$^-$	355, 231, 213, 143, 125	327, 303, 285, 267, 249, 225, 207, 183, 165, 141, 101
	56	HDMF *O*-glucoside	14.6	$C_{12}H_{18}O_8$	19.4; 20.9; 21.3	$C_{15}H_{20}O_{11}$	377 [M+H]$^+$	331 [M-COOH]$^-$	341, 231, 213, 129, 109	313, 289, 271, 253, 235, 193, 181, 169, 127, 101
	57	Sotolon *O*-glucoside	12.6	$C_{12}H_{18}O_8$	17.8; 19.6; 19.9	$C_{15}H_{20}O_{11}$	399 [M+Na]$^+$		355, 337, 313, 271, 227, 151	

	58	Maple furanone *O*-glucoside	17.1	$C_{13}H_{20}O_8$	21.5; 23.2; 23.5	$C_{16}H_{22}O_{11}$	413 $[M+Na]^+$		369, 351, 327, 271, 227, 165	
Pyranone glycoside	59	Maltol *O*-glucoside	11.7	$C_{12}H_{16}O_8$	16.6; 18.6	$C_{15}H_{18}O_{11}$	375 $[M+H]^+$		127	
	60	Ethylmaltol *O*-glucoside	16.9	$C_{13}H_{18}O_8$	21.3; 23.4	$C_{16}H_{20}O_{11}$	389 $[M+H]^+$		141	
Phenyl-propenyl glycoside	61	Cinnamyl alcohol *O*-glucoside	29.3	$C_{15}H_{20}O_6$	33.4	$C_{18}H_{22}O_9$	405 $[M+Na]^+$		385, 361, 343, 319, 301, 287, 243	
Mono-terpenol glycoside	62	Borneol *O*-glucoside	38.3	$C_{16}H_{28}O_6$	38.7	$C_{19}H_{30}O_9$	425 $[M+Na]^+$		381, 363, 339, 245	
	63	Geranyl *O*-glucoside	38.7	$C_{16}H_{28}O_6$	39.0	$C_{19}H_{30}O_9$	425 $[M+Na]^+$		381, 364, 339, 289, 245	
Alkyl glycoside	64	Cis-3-hexenol *O*-glucoside	26.8	$C_{12}H_{22}O_6$	32.4	$C_{15}H_{24}O_9$	371 $[M+Na]^+$		327, 285	
	65	Hexyl *O*-glucoside	31.8	$C_{12}H_{24}O_6$	36.2	$C_{15}H_{26}O_9$	373 $[M+Na]^+$		329, 287	
Lignan glycoside	66	Secoisolariciresinol *O*-diglucoside	27.7	$C_{32}H_{46}O_{16}$	30.1	$C_{35}H_{48}O_{19}$		771 [M-H]-		727, 667
Benzoic acid ester glycoside	67	Methyl anthranilate *N*-glucoside	26.3	$C_{14}H_{19}NO_7$	31.4	$C_{17}H_{21}NO_{10}$	400 $[M+H]^+$		382, 364, 346, 278, 242, 194, 152, 120	

Table S4. List of acceptors that could not be acylated by the FaMATs.

Compound group	No.	Compound name	Formula	Molecular weight (g/mol)
Rhamnosides	68	Quercetin-3-*O*-rhamnoside	$C_{21}H_{20}O_{11}$	448.4
Rhamnoglucosides (linkage not specified)	69	Apigenin-7-*O*-rhamnoglucoside	$C_{27}H_{30}O_{14}$	578.5
	70	Naringenin-7-*O*-rhamnoglucoside	$C_{27}H_{32}O_{14}$	580.5
	71	3´-Methylmyricetin-3-*O*-rhamnoglucoside	$C_{8}H_{32}O_{17}$	640.5
	72	Kaempferol-3-*O*-rhamnoglucoside	$C_{27}H_{30}O_{15}$	594.5
	73	Kaempferol-7-*O*-rhamnoglucoside	$C_{27}H_{30}O_{15}$	594.5
	74	Isosakuranetin-7-*O*-rhamnoglucoside	$C_{28}H_{34}O_{14}$	594.6
Arabinosides	75	Cyanidin-3-*O*-arabinoside chloride	$C_{20}H_{19}ClO_{10}$	454.8
	76	Quercetin-3-*O*-arabinoside	$C_{20}H_{18}O_{11}$	434.3
Arabinoglucosides	77	Quercetin-3-O-arabinoglucoside	$C_{26}H_{28}O_{16}$	596.49
Rutinoside	78	Quercetin-3-*O*-rutinoside	$C_{27}H_{30}O_{16}$	610.5
	79	Kaempferol-3-*O*-rutinoside	$C_{27}H_{30}O_{15}$	594.52
	80	Isorhamnetin-3-*O*-rutinoside	$C_{28}H_{32}O_{16}$	624.54
	81	Cyanidin-3-*O*-rutinoside	$C_{27}H_{31}ClO_{15}$	630.98
	82	Pelargonidin-3-*O*-rutinoside	$C_{27}H_{31}O_{14}^{+}$	579.5
C-glucosides	83	Apigenin-6-*C*-glucoside-7-*O*-glucoside	$C_{27}H_{30}O_{15}$	594.5
	84	Apigenin-6,8-di-*C*-glucoside	$C_{27}H_{30}O_{15}$	594.5
	85	Luteolin-6,8-di-*C*-glucoside	$C_{27}H_{30}O_{16}$	610.52
	86	Apigenin-6-*C*-glucoside-8-*C*-arabinoside	$C_{26}H_{28}O_{14}$	564.5
Sophoroside	87	Kaempferol-3-*O*-sophoroside-7-*O*-glucoside	$C_{33}H_{40}O_{21}$	772.7

Table S5. The ^{1}H-NMR of maple furanone (6'-*O*-malonyl) glucoside isomer 1 and isomer 2

^{1}H	Maple furanone malonyl glucoside isomer 1		Maple furanone malonyl glucoside isomer 2		Reference
	δ		δ		
Aglycone					
5	4.90	1H, *m*	4.91	1H, *m*	
6	1.52	2H, *tt* (13.7, 7.4)	1.53	2H, *ddt* (21.6, 14.2, 7.1)	1
7	0.77	3H, *t* (7.3)	0.77	3H, *t* (7.5)	
8	1.89	3H, *s*	1.89	3H, *s*	
Glucosyl					
1'	4.94	1H, *d* (7.8)	5.04	1H, *d* (7.9)	
2'	3.23	1H, *t* (9.0)	3.23	1H, *m*	
3'	3.13-3.15	1H, *m*	3.12-3.15	1H, *m*	
4'	3.13-3.15	1H, *m*	3.12-3.15	1H, *m*	
5'	3.13-3.15	1H, *m*	3.12-3.15	1H, *m*	
6'	4.02	1H, H-6'a, *dd* (11.9, 7.0)	4.04	1H, H-6'a, *dd* (11.9, 7.0)	
	4.21	1H, H-6'b, *d* (11.9)	4.25	1H, H-6'b, *dd* (11.9, 2.1)	
Malonyl					
2''	3.40	2H, *s*	3.45	2H, *s*	

1200 MHz, δ in ppm, *J* in Hz, in DMSO-d_6

Table S6. The ^{13}C-NMR of maple furanone (6'-*O*-malonyl) glucoside isomer 1 and isomer 2

^{13}C	Maple furanone malonyl glucoside isomer 1	Maple furanone malonyl glucoside isomer 2	Reference
	δ	δ	
Aglycone			
2	167.98	167.94	
3	142.09	141.96	
4	137.78	137.50	
5	80.83	80.83	1
6	24.88	25.04	
7	7.94	7.98	
8	10.52	10.43	
Glucosyl			
1'	100.92	100.59	
2'	76.60	76.51	
3'	73.86	73.86	
4'	70.29	70.37	
5'	77.76	77.68	
6'	63.98	63.90	
Malonyl			
1''	170.80	170.90	
2''	59.0	61.07	
3''	170.47	170.53	

300 MHz, δ in ppm, in DMSO-d_6

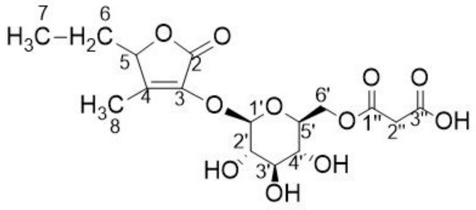

FIGURES

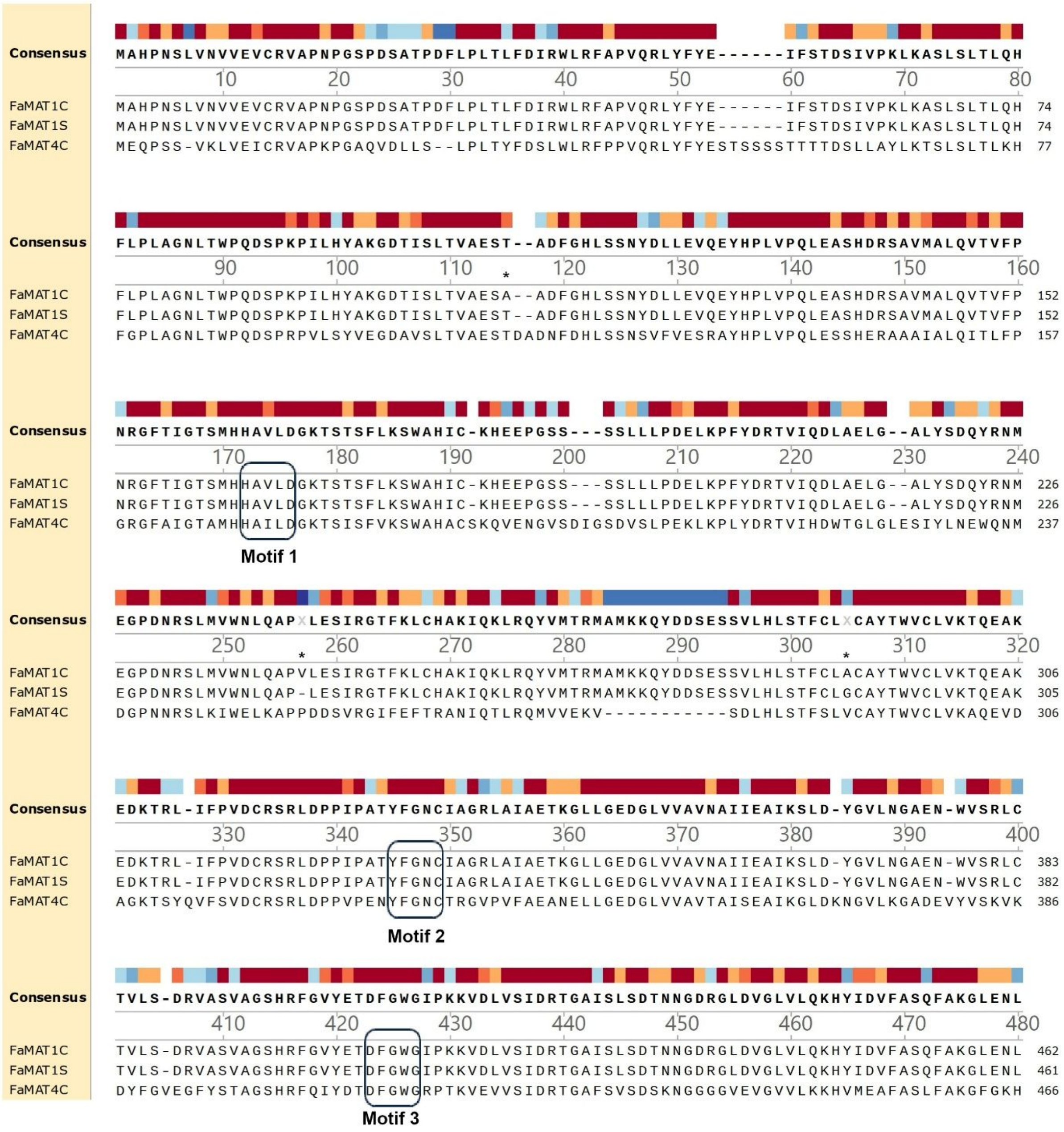

Figure S1. Multiple sequence alignment of FaMAT1C, FaMAT4C$_1$/S$_1$, and FaMAT1S protein using MUSCLE (www.ebi.ac.uk/jdispatcher/msa/muscle?stype=protein). Three conserved motifs contribute to the catalytic activity. The asterisks show the different amino acids between FaMAT1C and FaMAT1S.

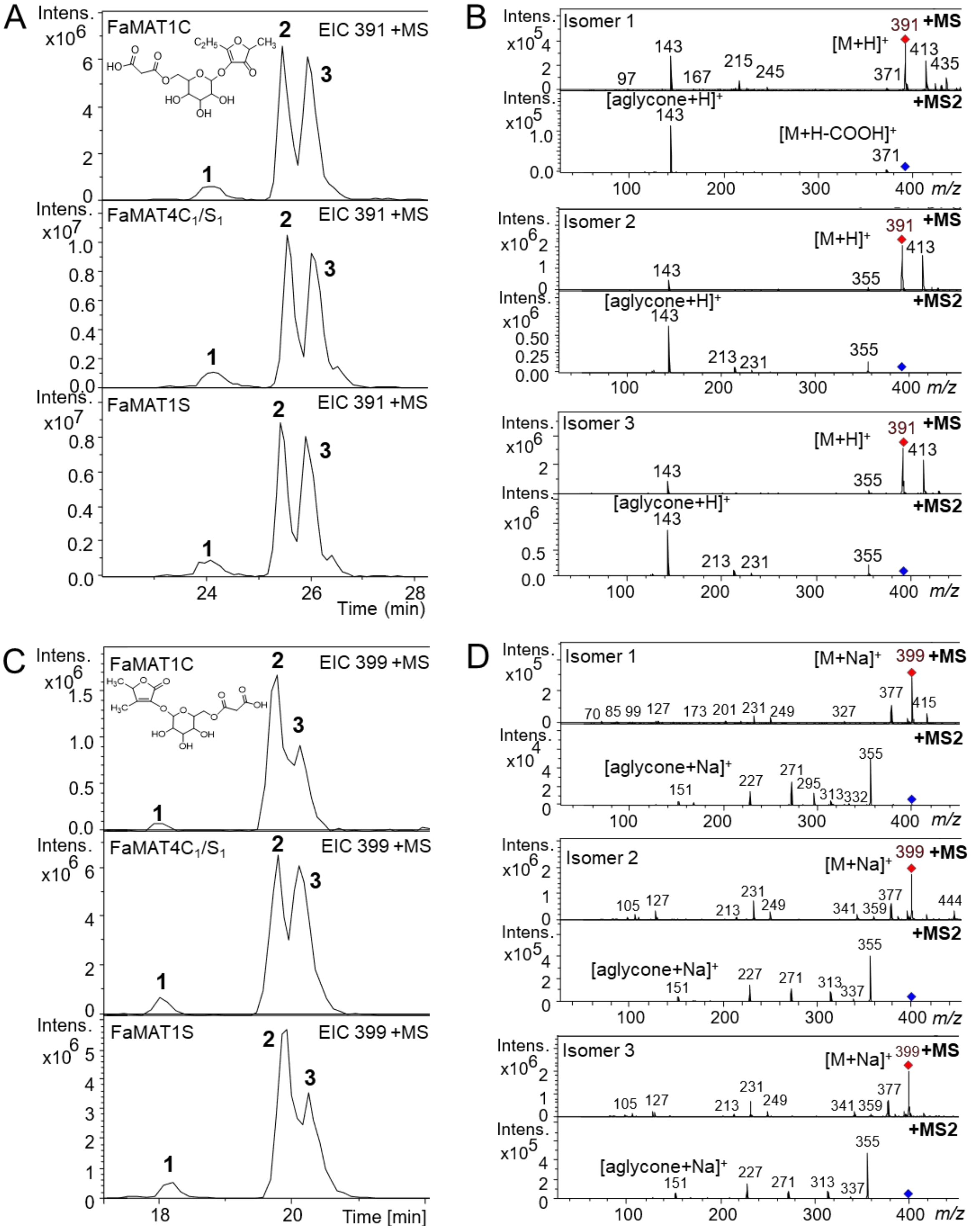

Figure S2. LC-MS analysis of malonylated products. Extracted ion chromatogram (EIC) of the products formed by FaMAT1C, FaMAT1S, and FaMAT4C$_1$/S$_1$ from 5-EHMF-glucoside **55** (A) and sotolon glucoside **57** (C). MS and MS2 data of malonylated products formed from 5-EHMF-glucoside (B) and sotolon glucoside (D).

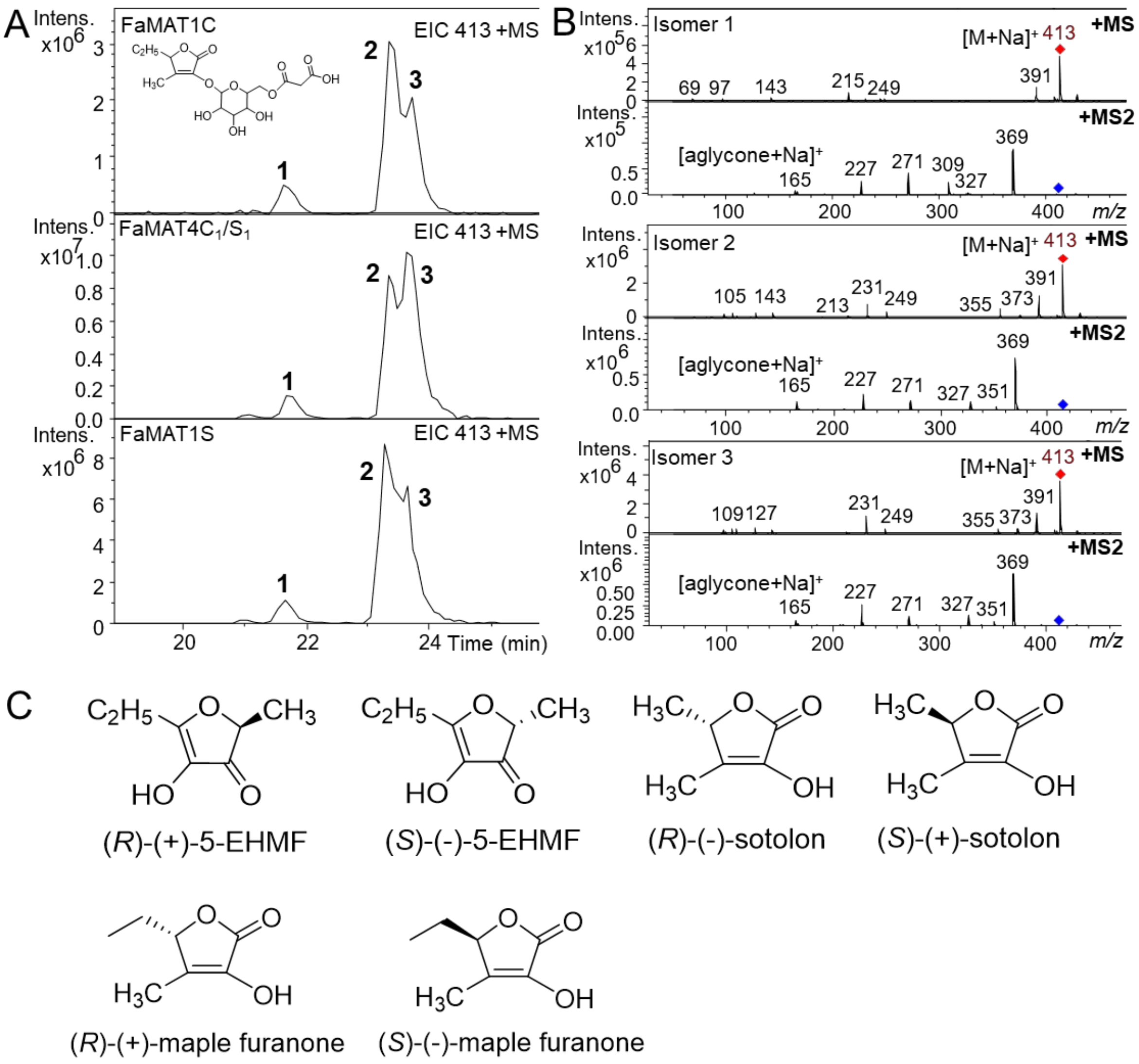

Figure S3. LC-MS analysis of malonylated products. Extracted ion chromatogram (EIC) of the products formed by FaMAT1C, FaMAT1S, and FaMAT4C$_1$/S$_1$ from maple furanone glucoside **58** (A). MS and MS2 data of malonylated products formed from maple furanone glucoside (B). Configuration of the enantiomers of 5-EHMF, sotolon, and maple furanone according to Nakahashi et al.[1] and Monde et al.[2] (C).

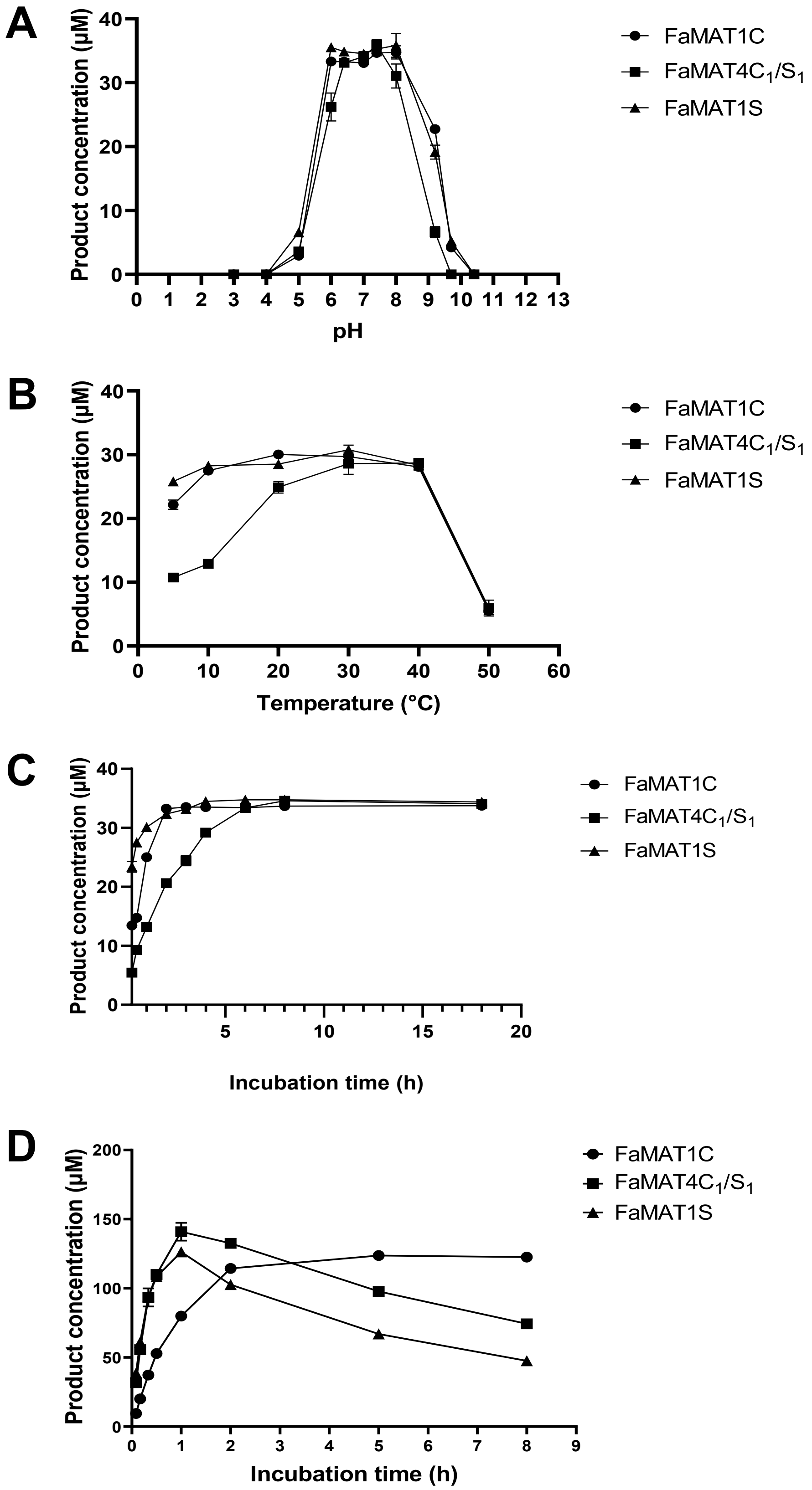

Figure S4. Effect of pH (A), temperature (B), and incubation time (C) on the enzymatic activity of FaMATs (2.5 µg) using quercetin-3-*O*-glucoside as the acyl acceptor. Time course experiment with FaMATs (25 µg) and HDMF *O*-glucoside as the substrate (D).

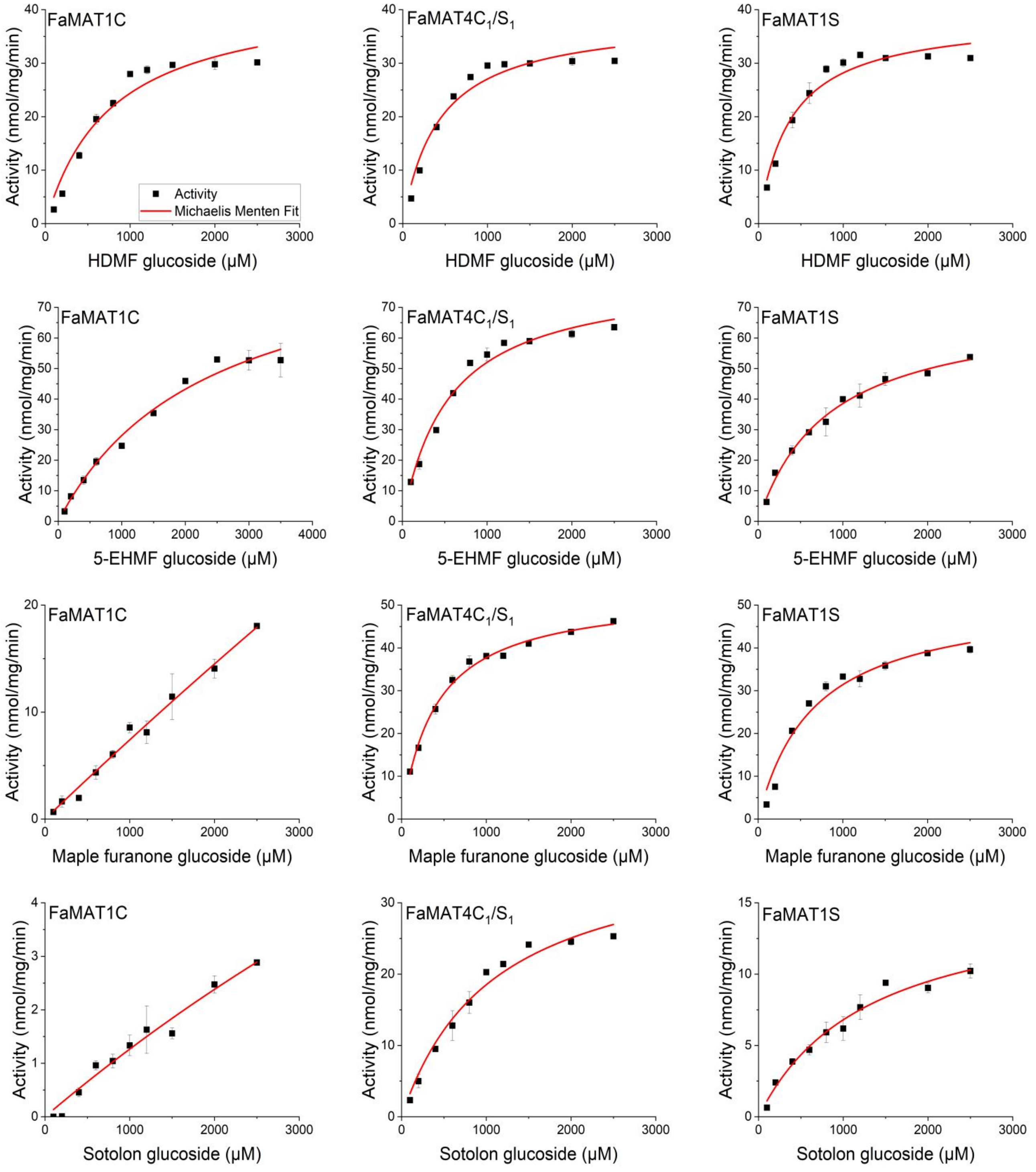

Figure S5. Michaelis-Menten diagrams of FaMATs using HDMF *O*-glucoside, 5-EHMF *O*-glucoside, maple furanone *O*-glucoside, and sotolon *O*-glucoside as acceptors. The plots illustrate the relationship between acceptor concentration and enzyme activity.

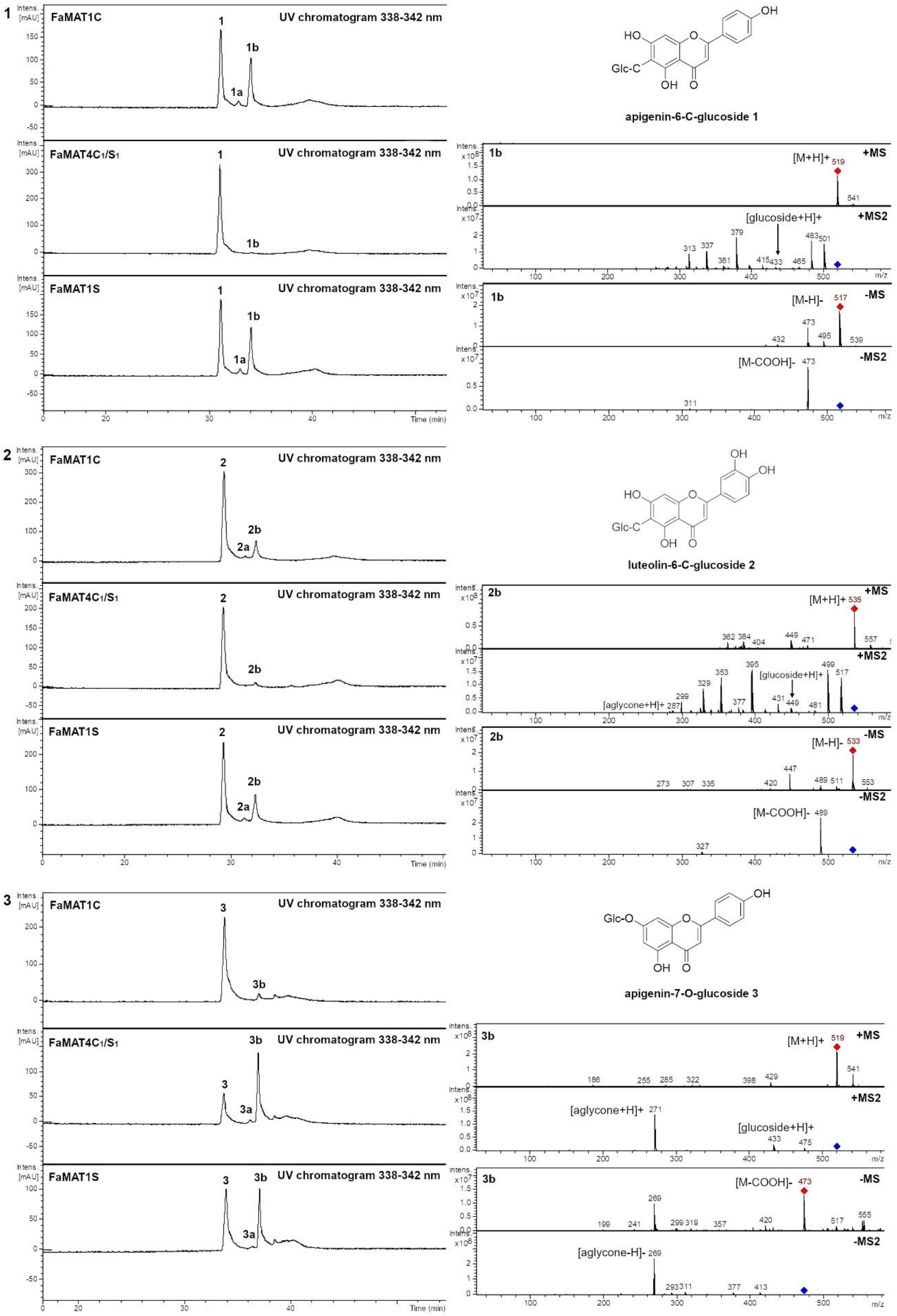

Figure S6. LC-MS analysis of the substrates and corresponding products. UV chromatogram of apigenin-6-*C*-glucoside **1**, luteolin-6-*C*-glucoside **2**, apigenin-7-*O*-glucoside **3**, and their malonylated products **1a**, **1b**, **2a**, **2b**, **3a**, and **3b**, respectively. Mass spectra (MS) and product ion spectra (MS2) of the malonylated products are shown on the right-hand side. + positive mode, - negative mode. Pseudo-molecular ions are marked in red and blue.

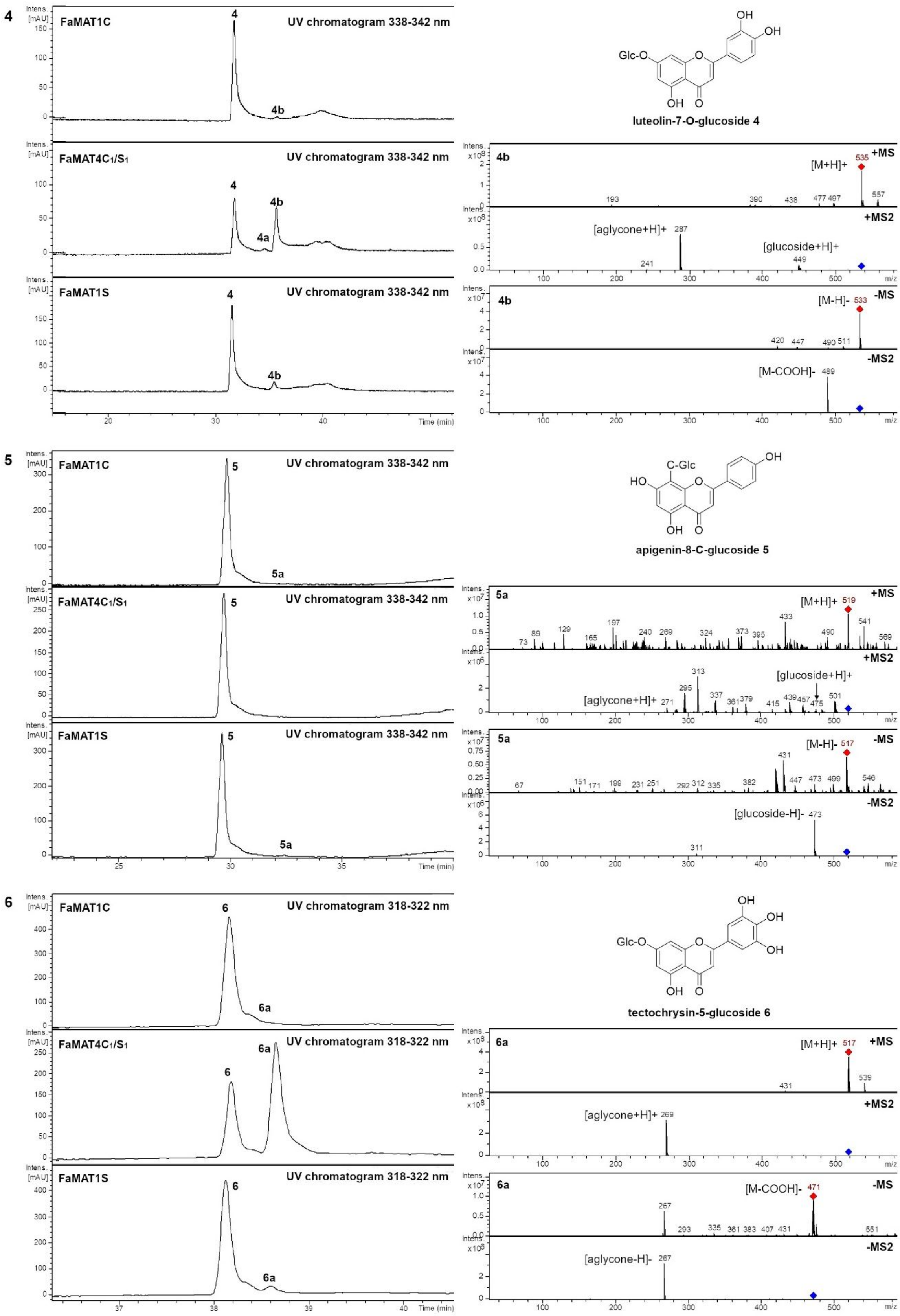

Figure S7. LC-MS analysis of the substrates and corresponding products. UV chromatogram of luteolin-7-*O*-glucoside **4**, apigenin-8-*C*-glucoside **5**, tectochrysin-5-*O*-glucoside **6**, and their malonylated products **4a**, **4b**, **5a**, and **6a**, respectively. Mass spectra (MS) and product ion spectra (MS2) of the malonylated products are shown on the right-hand side. + positive mode, - negative mode. Pseudo-molecular ions are marked in red and blue.

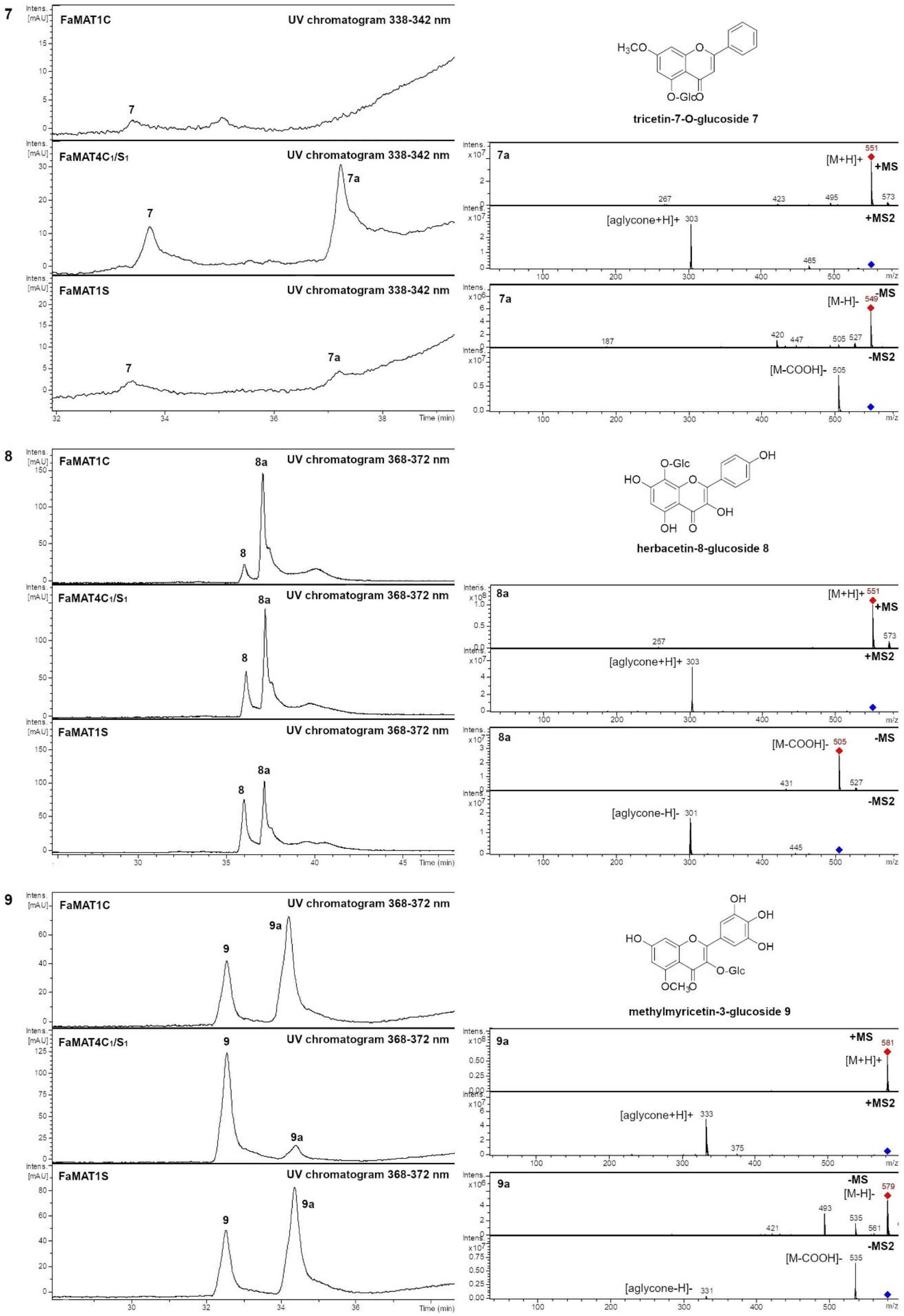

Figure S8. LC-MS analysis of the substrates and corresponding products. UV chromatogram of tricetin-7-*O*-glucoside **7**, herbacetin-8-*O*-glucoside **8**, methylmyricetin-3-*O*-glucoside **9**, and their malonylated products **7a**, **8a**, and **9a**, respectively. Mass spectra (MS) and product ion spectra (MS2) of the malonylated products are shown on the right-hand side. + positive mode, - negative mode. Pseudo-molecular ions are marked in red and blue.

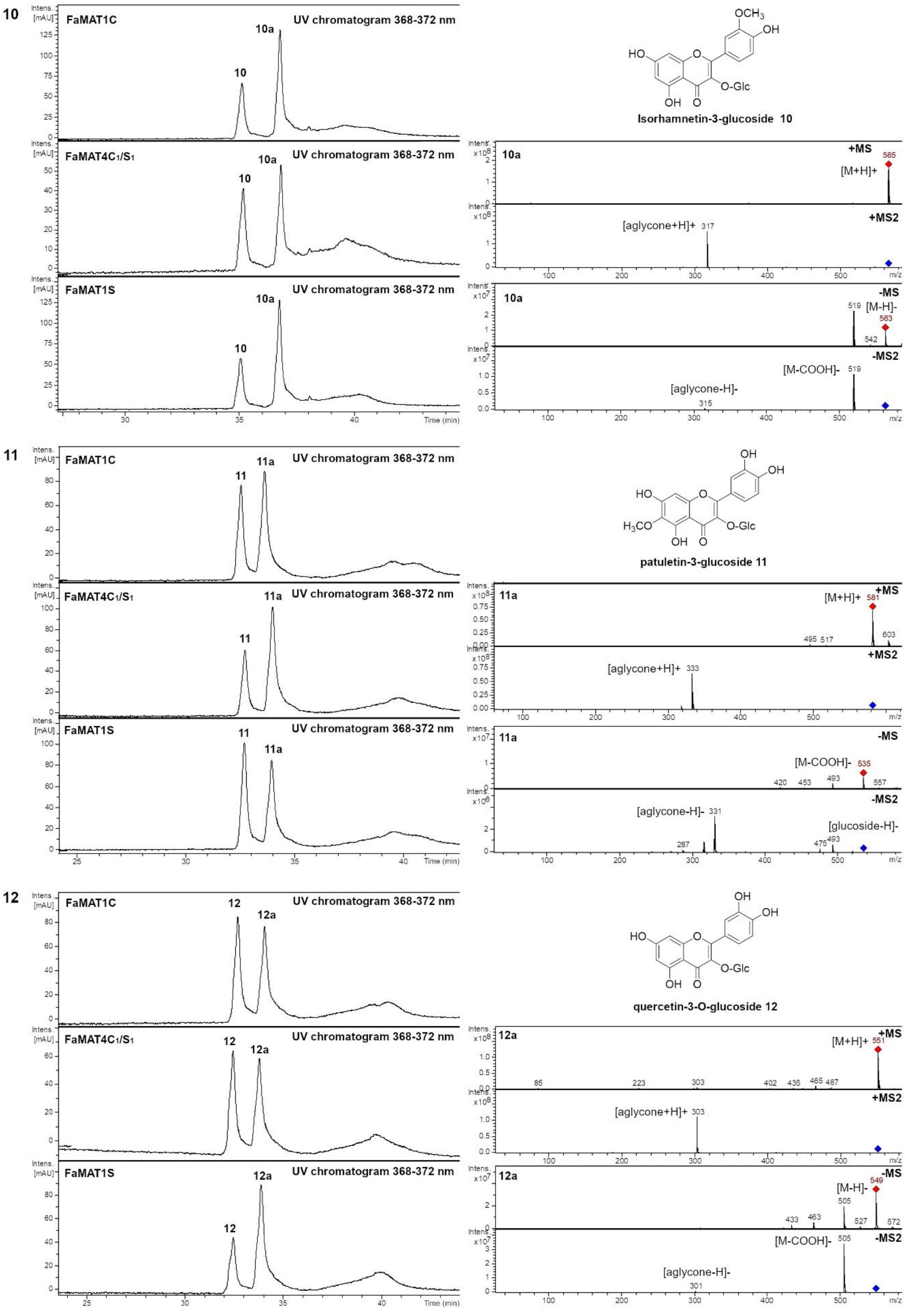

Figure S9. LC-MS analysis of the substrates and corresponding products. UV chromatogram of isorhamnetin-3-*O*-glucoside **10**, patuletin-3-*O*-glucoside **11**, quercetin-3-*O*-glucoside **12**, and their malonylated products **10a**, **11a**, and **12a**, respectively. Mass spectra (MS) and product ion spectra (MS2) of the malonylated products are shown on the right-hand side. + positive mode, - negative mode. Pseudo-molecular ions are marked in red and blue.

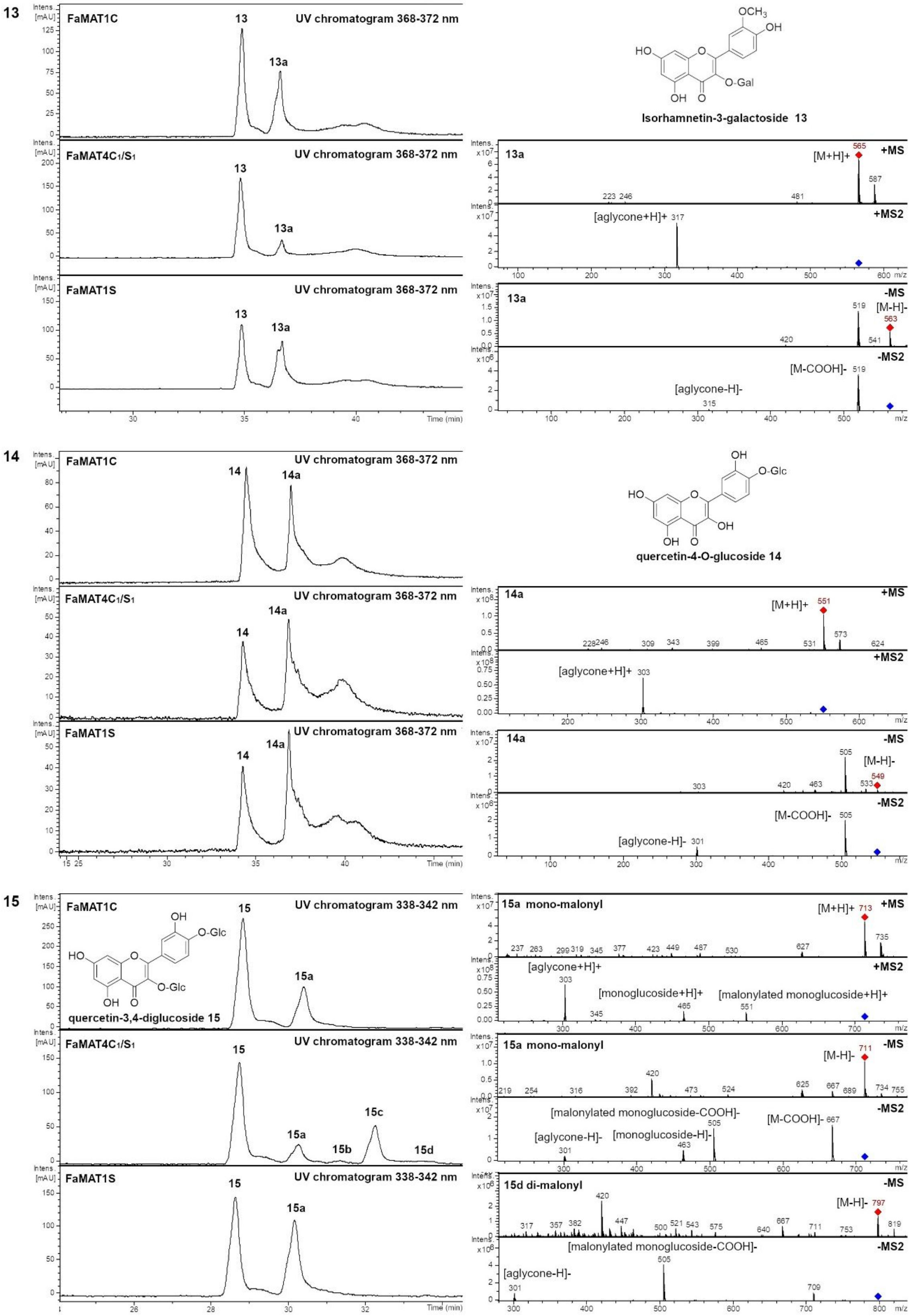

Figure S10. LC-MS analysis of the substrates and corresponding products. UV chromatogram of isorhamnetin 3-*O*-galactoside **13**, quercetin-4-*O*-glucoside **14**, quercetin-3,4-di-*O*-glucoside **15**, and their malonylated products **13a**, **14a**, **15a** (mono-malonyl)**, 15b** (mono-malonyl)**, 15c** (mono-malonyl), and **15d** (di-malonyl) respectively. Mass spectra (MS) and product ion spectra (MS2) of the malonylated products are shown on the right-hand side. + positive mode, - negative mode. Pseudo-molecular ions are marked in red and blue.

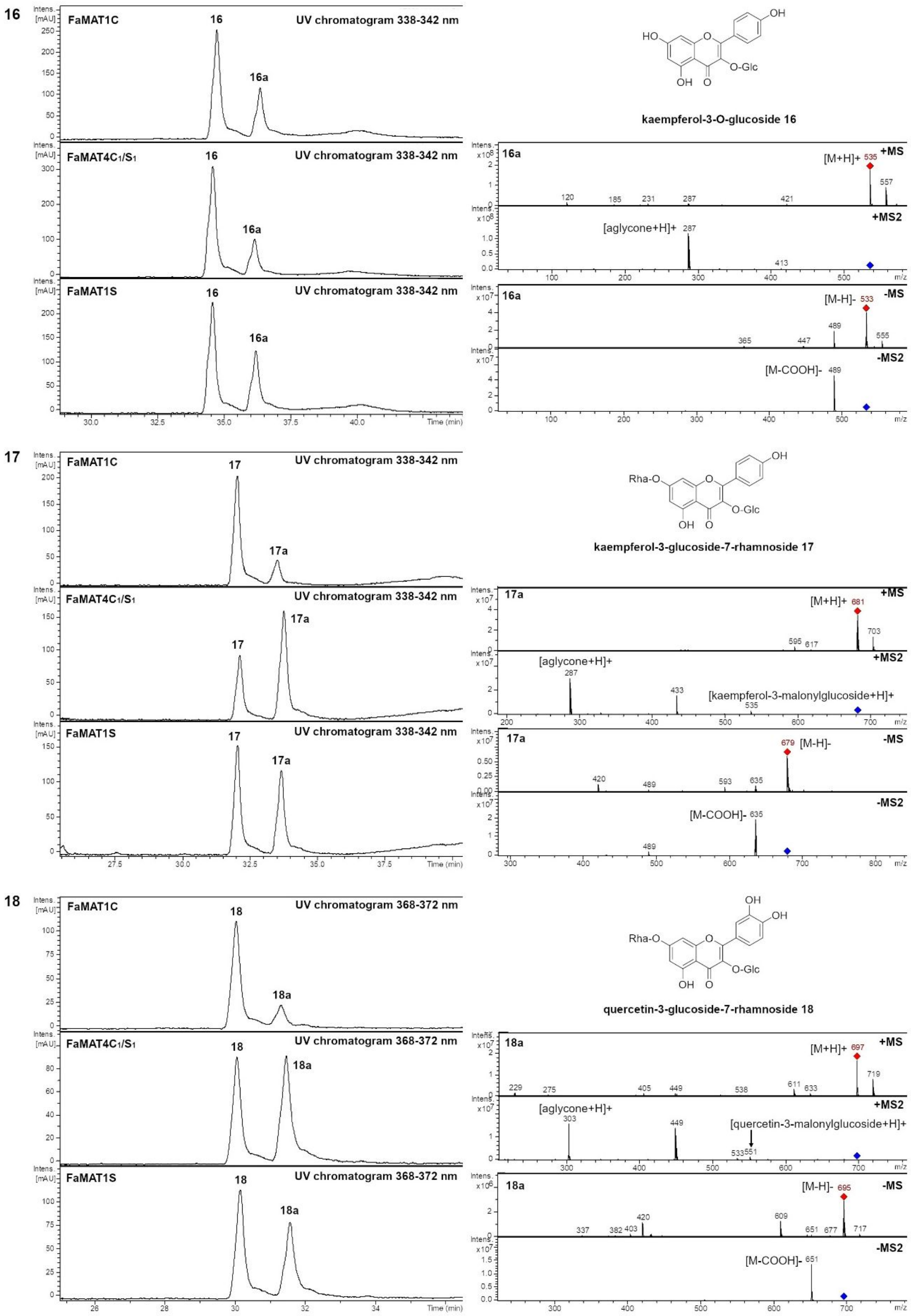

Figure S11. LC-MS analysis of the substrates and corresponding products. UV chromatogram of kaempferol-3-*O*-glucoside **16**, kaempferol-3-*O*-glucoside-7-*O*-rhamnoside **17**, quercetin-3-*O*-glucoside-7-*O*-rhamnoside **18**, and their malonylated products **16a**, **17a**, and **18a**, respectively. Mass spectra (MS) and product ion spectra (MS2) of the malonylated products are shown on the right-hand side. + positive mode, - negative mode. Pseudo-molecular ions are marked in red and blue.

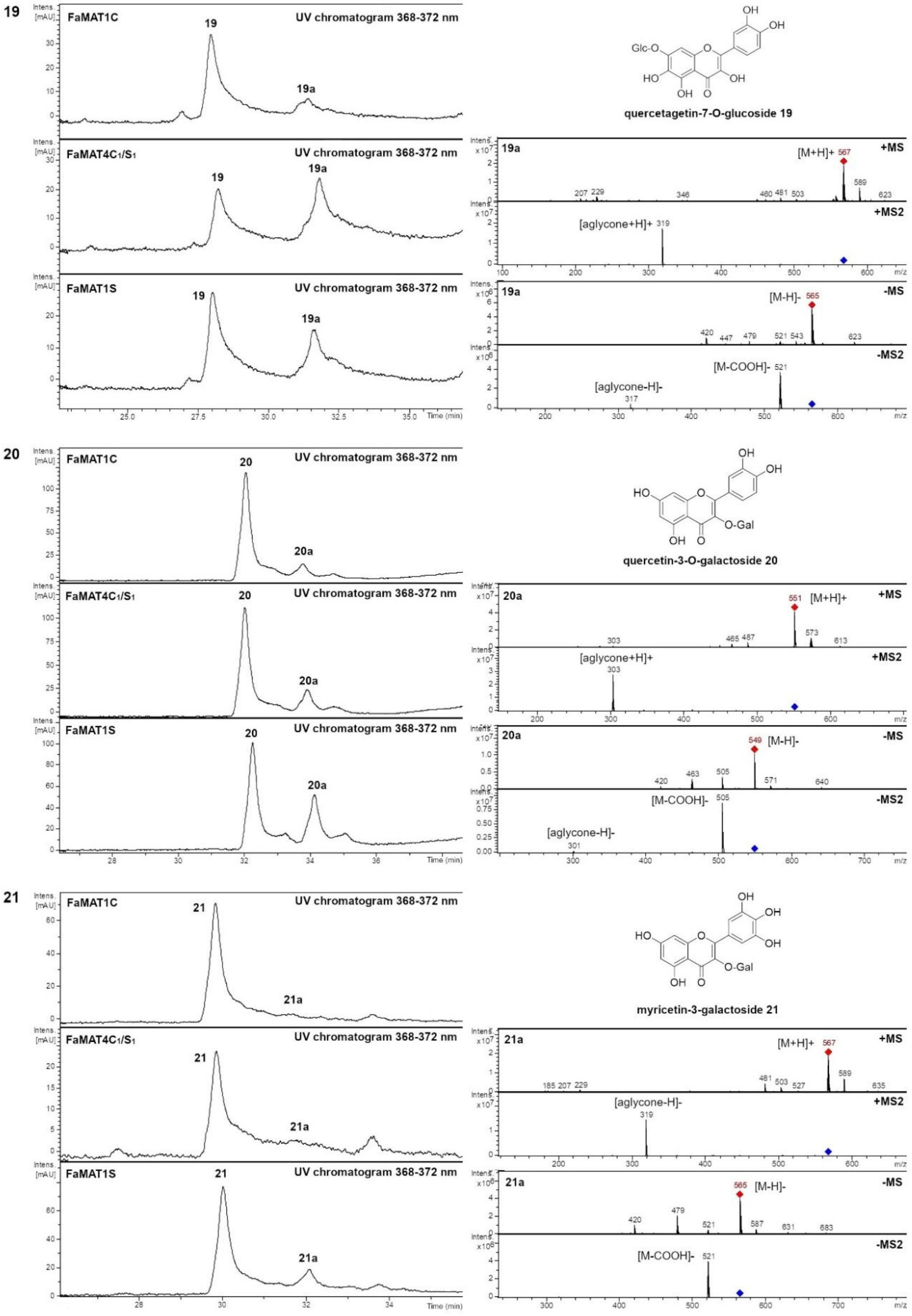

Figure S12. LC-MS analysis of the substrates and corresponding products. UV chromatogram of quercetagetin-7-*O*-glucoside **19**, quercetin-3-*O*-galactoside **20**, myricetin-3-*O*-galactoside **21**, and their malonylated products **19a**, **20a**, and **21a**, respectively. Mass spectra (MS) and product ion spectra (MS2) of the malonylated products are shown on the right-hand side. + positive mode, - negative mode. Pseudo-molecular ions are marked in red and blue.

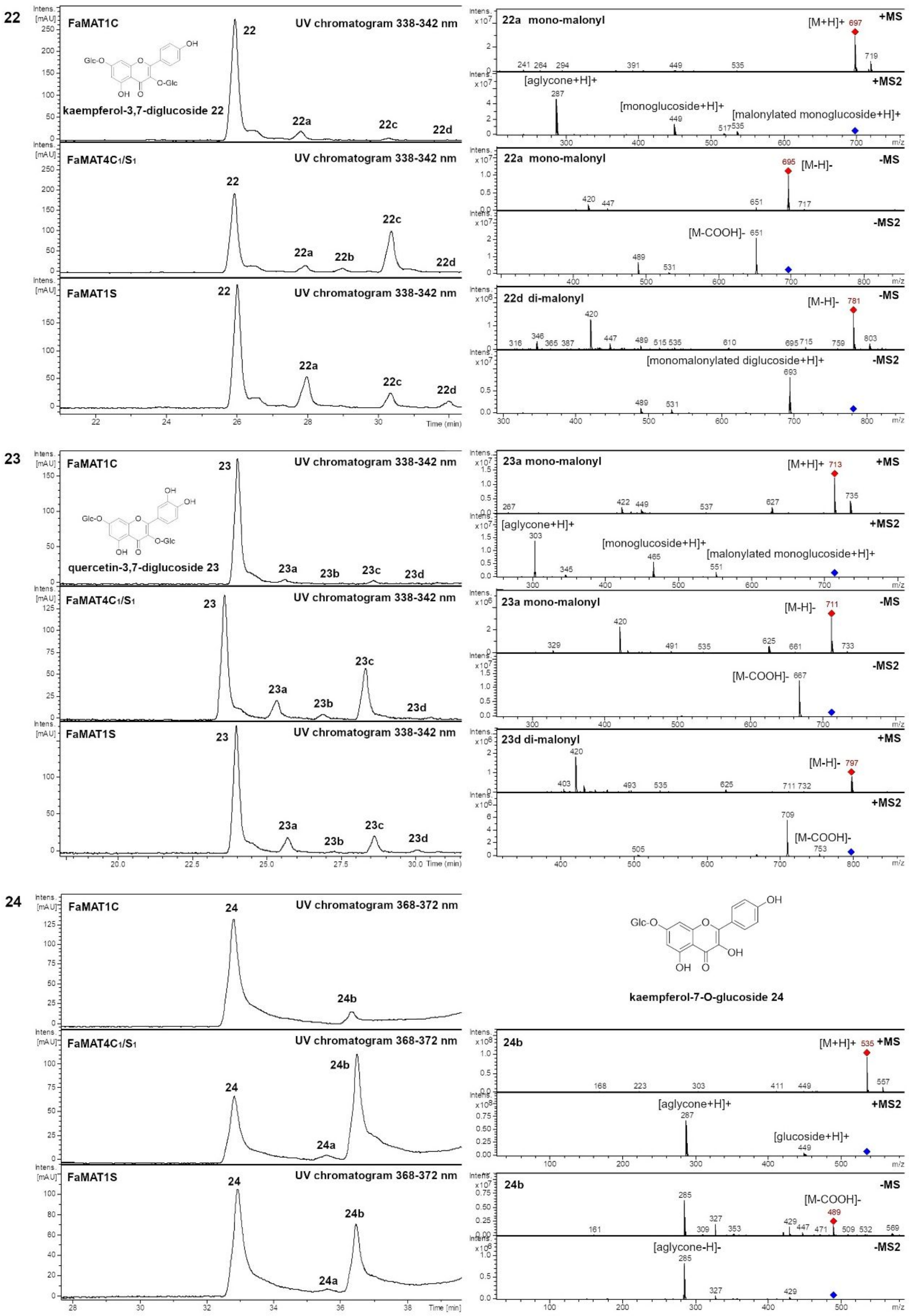

Figure S13. LC-MS analysis of the substrates and corresponding products. UV chromatogram of kaempferol-3,7-di-*O*-glucoside **22**, quercetin-3,7-di-*O*-glucoside **23**, kaempferol-7-*O*-glucoside **24**, and their malonylated products **22a**, **22b**, **22c**, **22d** (di-malonyl), **23a**, **23b**, **23c**, **23d** (di-malonyl), **24a**, and **24b**, respectively. Mass spectra (MS) and product ion spectra (MS2) of the malonylated products are shown on the right-hand side. + positive mode, - negative mode. Pseudo-molecular ions are marked in red and blue.

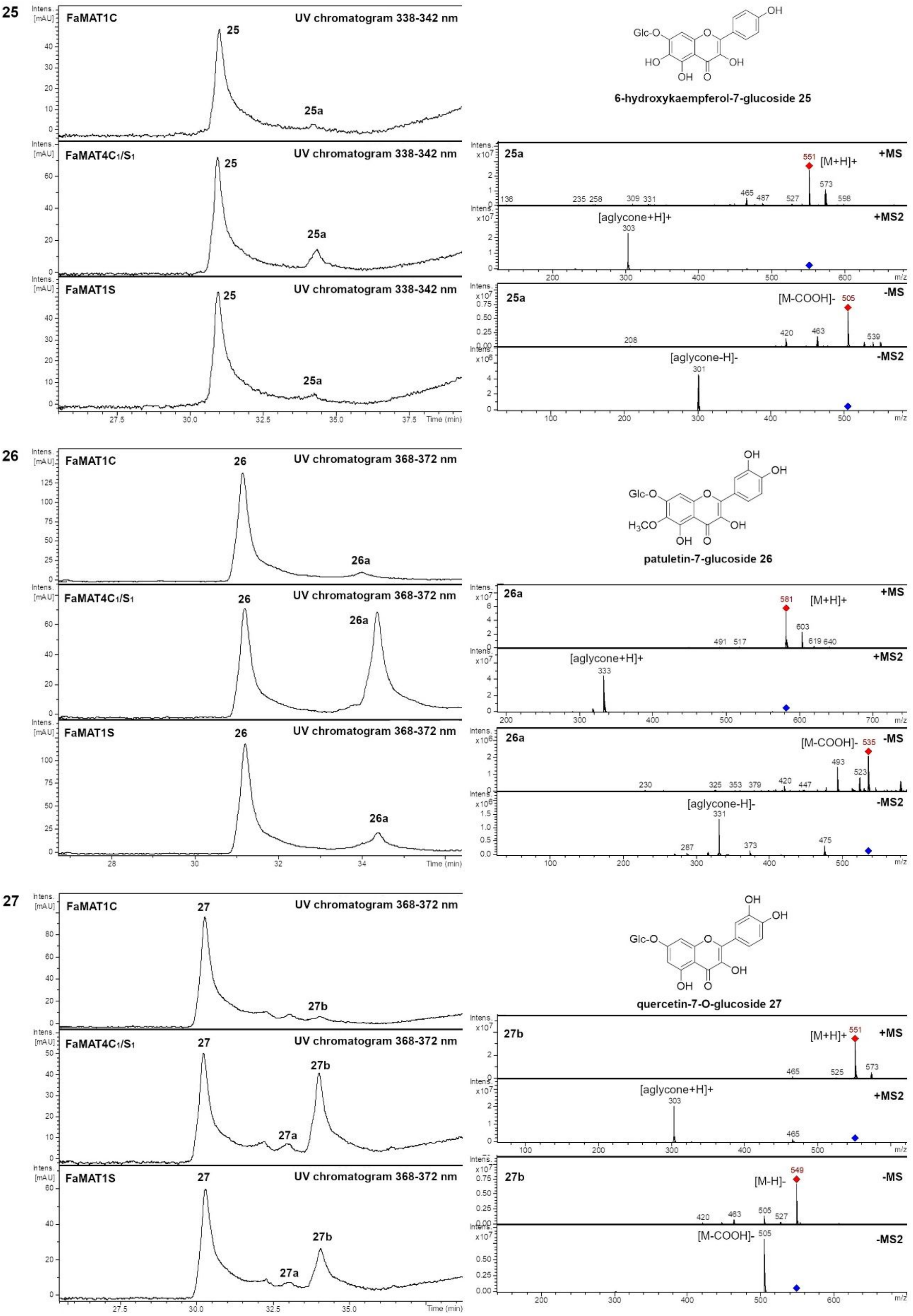

Figure S14. LC-MS analysis of the substrates and corresponding products. UV chromatogram of 6-hydroxykaempferol-7-*O*-glucoside **25**, patuletin-7-*O*-glucoside **26**, quercetin-7-*O*-glucoside **27**, and their malonylated products **25a**, **26a**, **27a**, and **27b**, respectively. Mass spectra (MS) and product ion spectra (MS2) of the malonylated products are shown on the right-hand side. + positive mode, - negative mode. Pseudo-molecular ions are marked in red and blue.

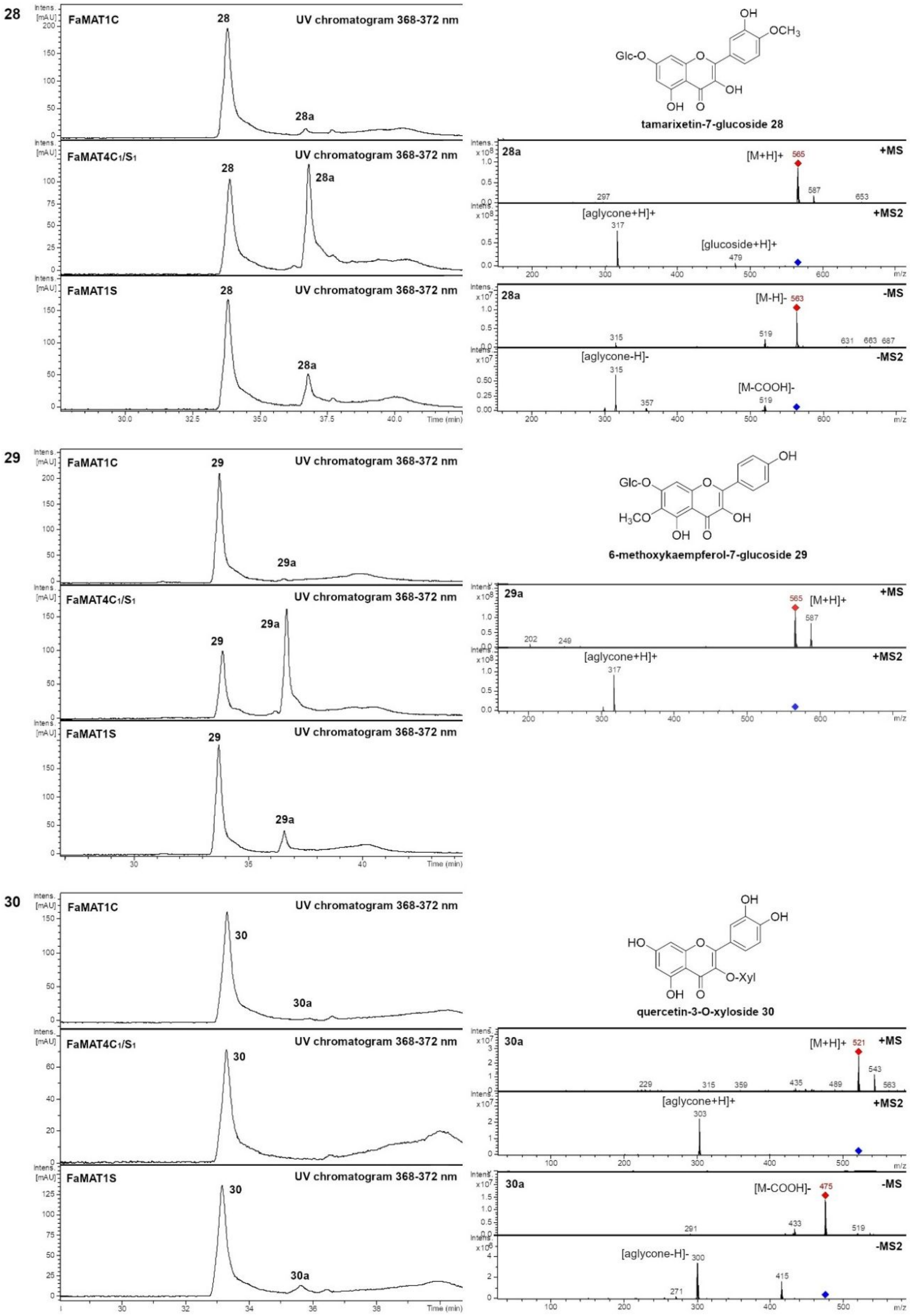

Figure S15. LC-MS analysis of the substrates and corresponding products. UV chromatogram of tamarixetin-7-*O*-glucoside **28**, 6-methoxykaempferol-7-*O*-glucoside **29**, quercetin-3-*O*-xyloside **30**, and their malonylated products **28a**, **29a**, and **30a**, respectively. Mass spectra (MS) and product ion spectra (MS2) of the malonylated products are shown on the right-hand side. + positive mode, - negative mode. Pseudo-molecular ions are marked in red and blue.

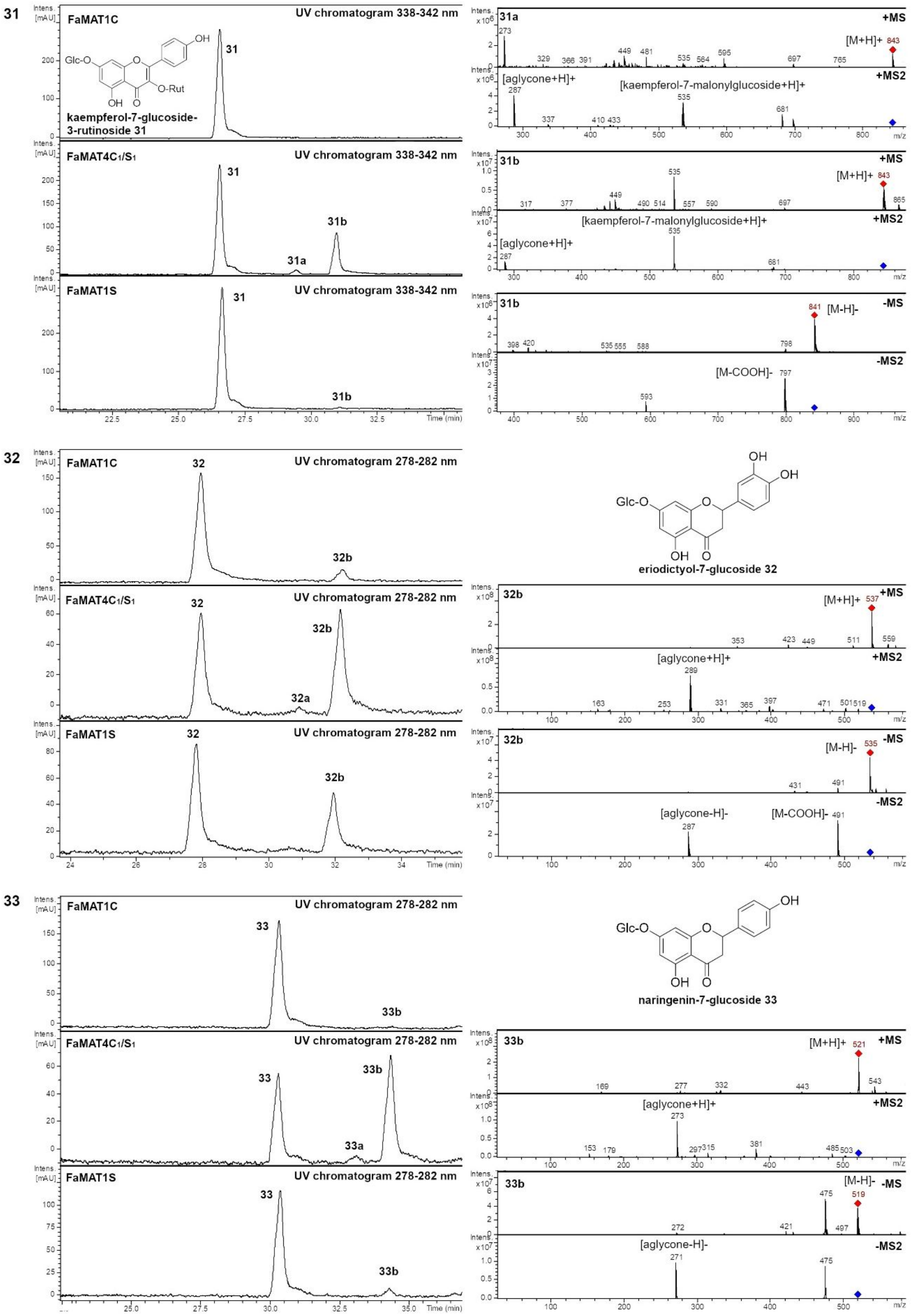

Figure S16. LC-MS analysis of the substrates and corresponding products. UV chromatogram of kaempferol-7-*O*-glucoside-3-rutinoside **31**, eriodictyol-7-*O*-glucoside **32**, naringenin-7-*O*-glucoside **33**, and their mono-malonylated products **31a**, **31b**, **32a**, **32b**, **33a** and **33b**, respectively. Mass spectra (MS) and product ion spectra (MS2) of the malonylated products are shown on the right-hand side. + positive mode, - negative mode. Pseudo-molecular ions are marked in red and blue.

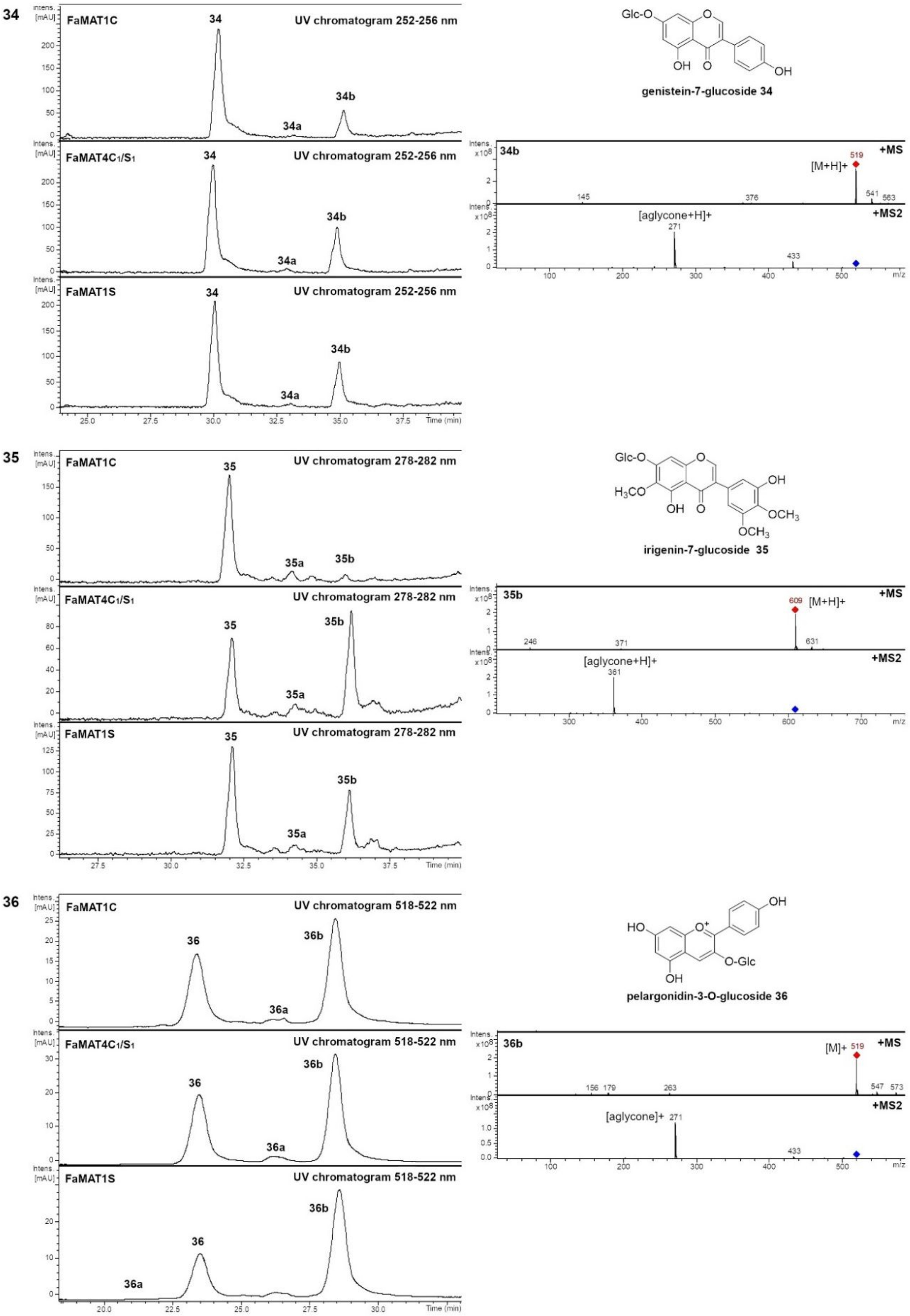

Figure S17. LC-MS analysis of the substrates and corresponding products. UV chromatogram of genistein-7-*O*-glucoside **34**, irigenin-7-*O*-glucoside **35**, pelargonidin-3-*O*-glucoside **36**, and their malonylated products **34a**, **34b**, **35a**, **35b**, **36a**, and **36b**, respectively. Mass spectra (MS) and product ion spectra (MS2) of the malonylated products are shown on the right-hand side. + positive mode, - negative mode. Pseudo-molecular ions are marked in red and blue.

37

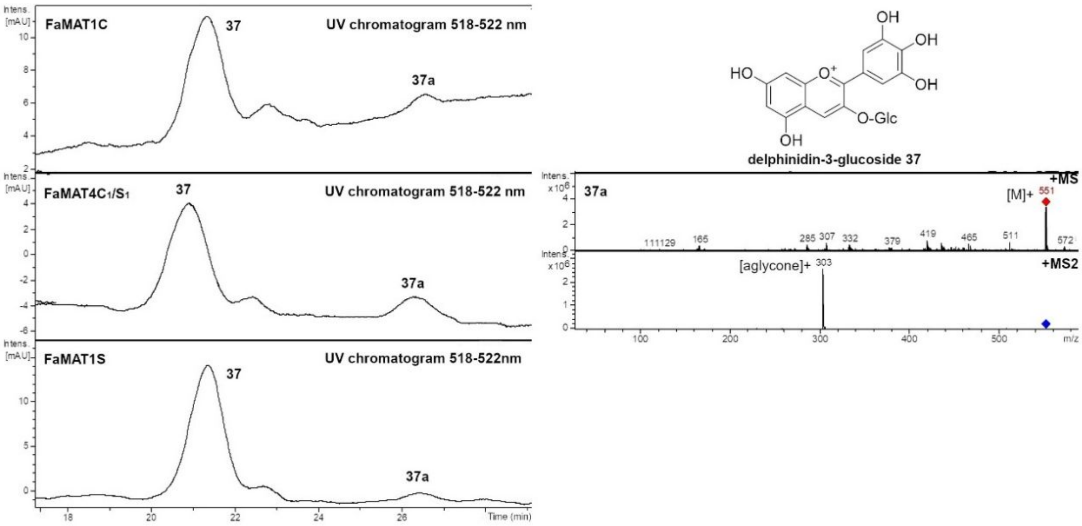

38

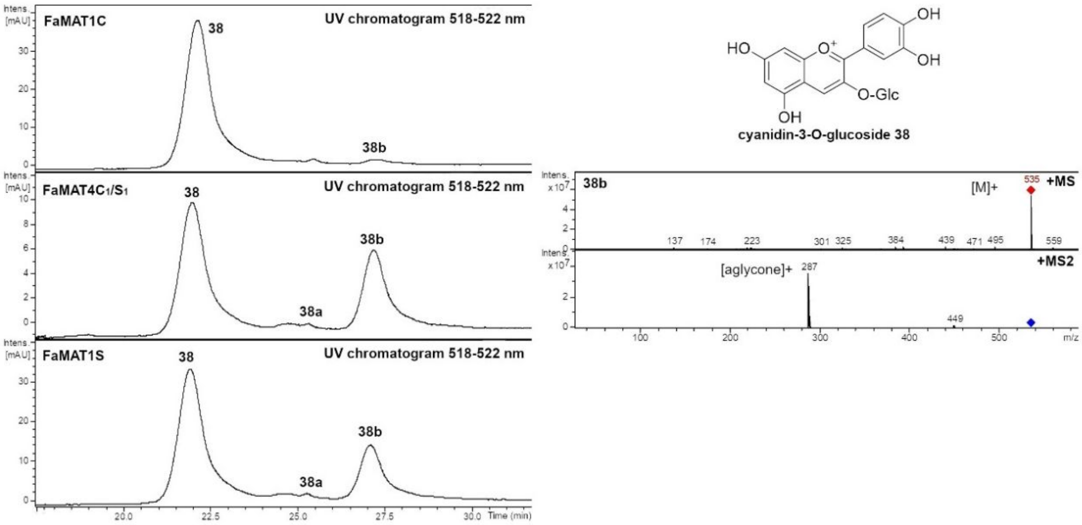

39

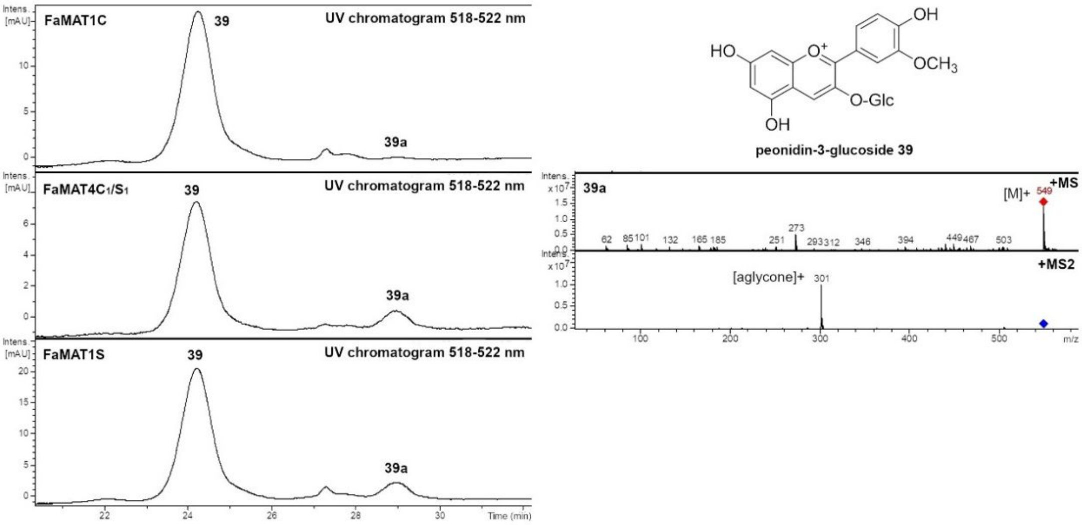

Figure S18. LC-MS analysis of the substrates and corresponding products. UV chromatogram of delphinidin-3-*O*-glucoside **37**, cyanidin-3-*O*-glucoside **38**, peonidin-3-*O*-glucoside **39**, and their malonylated products **37a**, **38a**, **38b**, and **39a**, respectively. Mass spectra (MS) and product ion spectra (MS2) of the malonylated products are shown on the right-hand side. + positive mode, **-** negative mode. Pseudo-molecular ions are marked in red and blue.

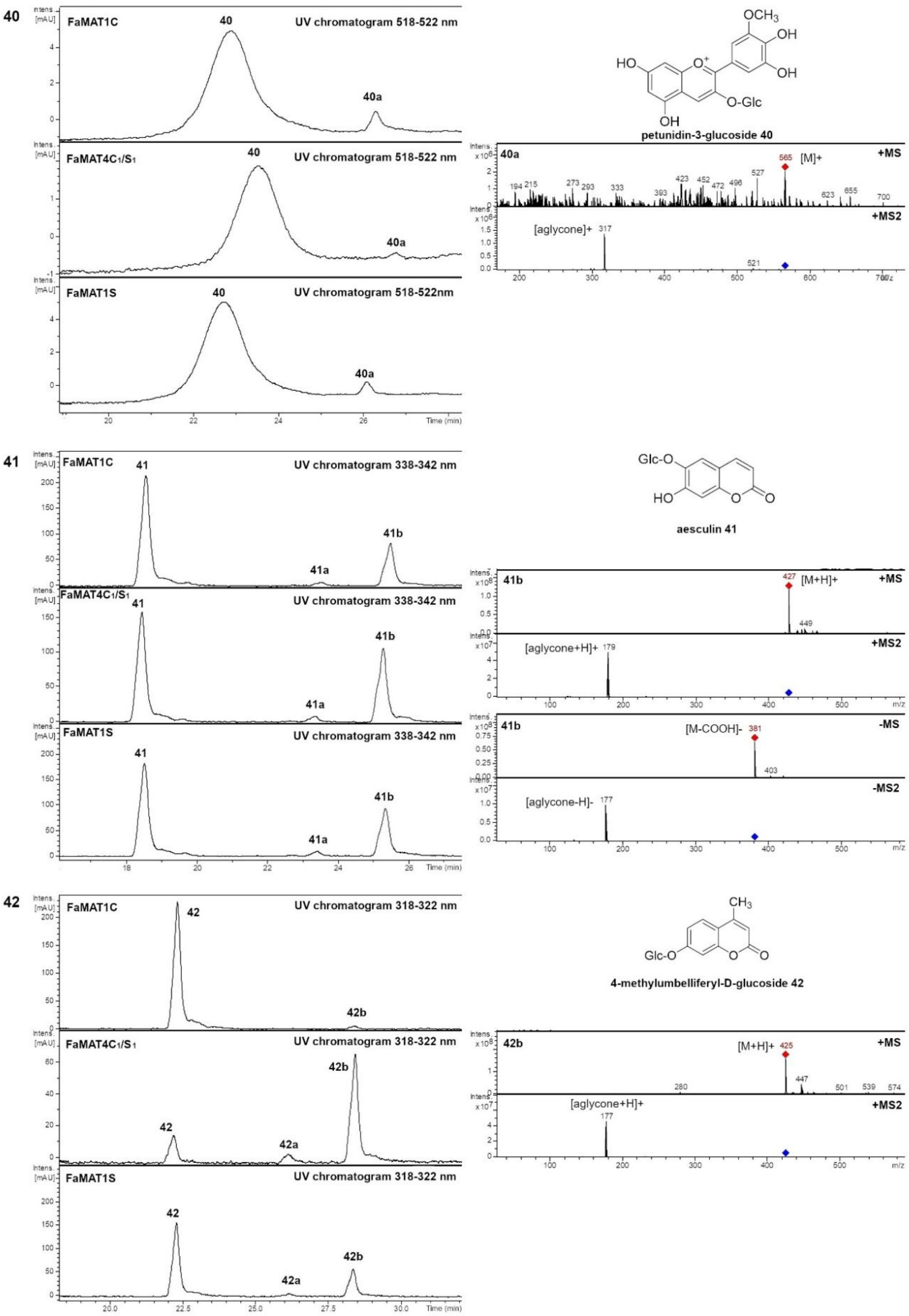

Figure S19. LC-MS analysis of the substrates and corresponding products. UV chromatogram of petunidin-3-*O*-glucoside **40**, aesculin **41**, 4-methylumbelliferyl-D-*O*-glucoside **42**, and their malonylated products **40a**, **41a**, **41b**, **42a**, and **42b**, respectively. Mass spectra (MS) and product ion spectra (MS2) of the malonylated products are shown on the right-hand side. + positive mode, - negative mode. Pseudo-molecular ions are marked in red and blue.

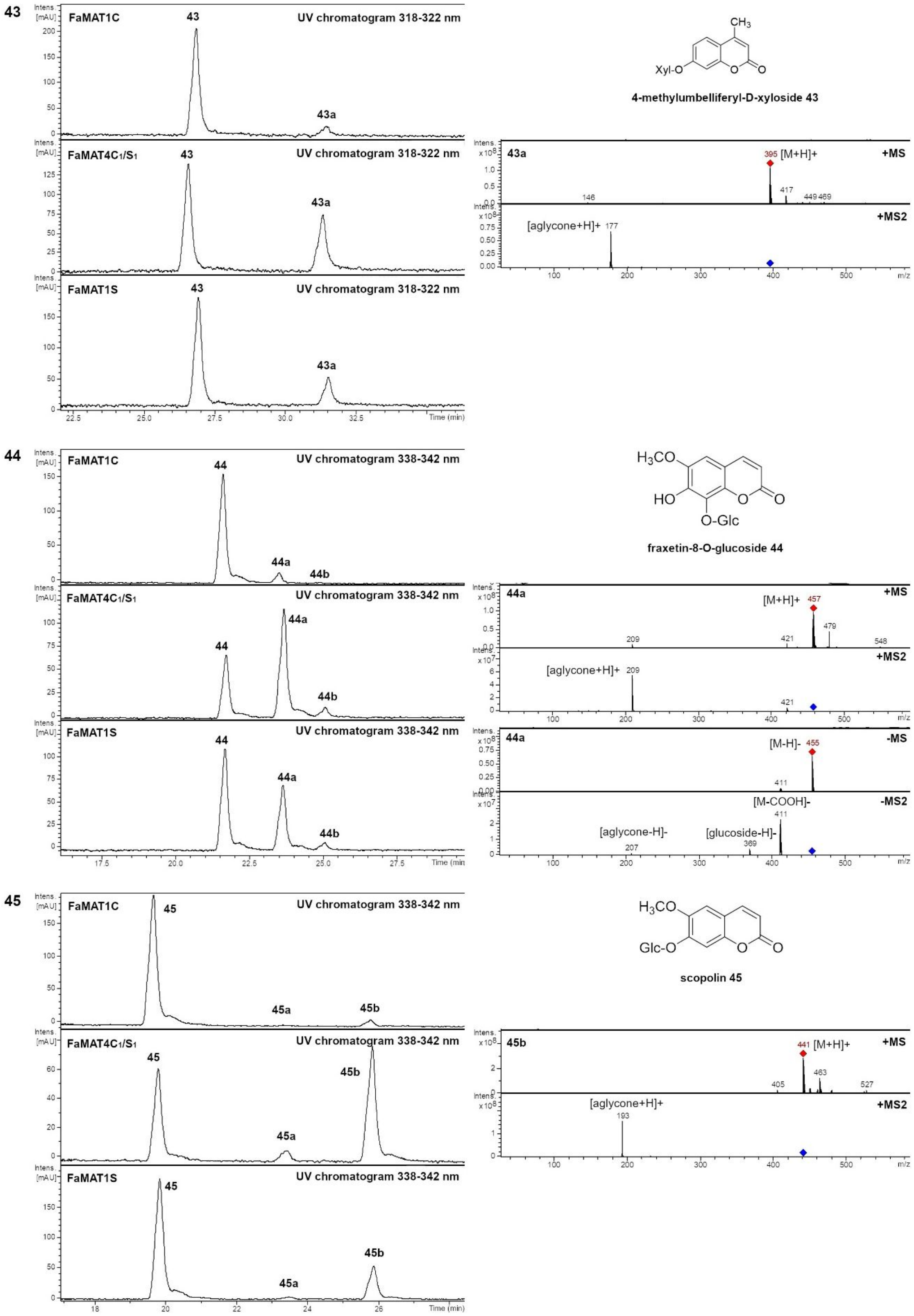

Figure S20. LC-MS analysis of the substrates and corresponding products. UV chromatogram of 4-methylumbelliferyl-D-*O*-xyloside **43**, fraxetin-8-*O*-glucoside **44**, scopolin **45**, and their malonylated products **43a**, **44a**, **44b**, **45a**, and **45b**, respectively. Mass spectra (MS) and product ion spectra (MS2) of the malonylated products are shown on the right-hand side. + positive mode, - negative mode. Pseudo-molecular ions are marked in red and blue.

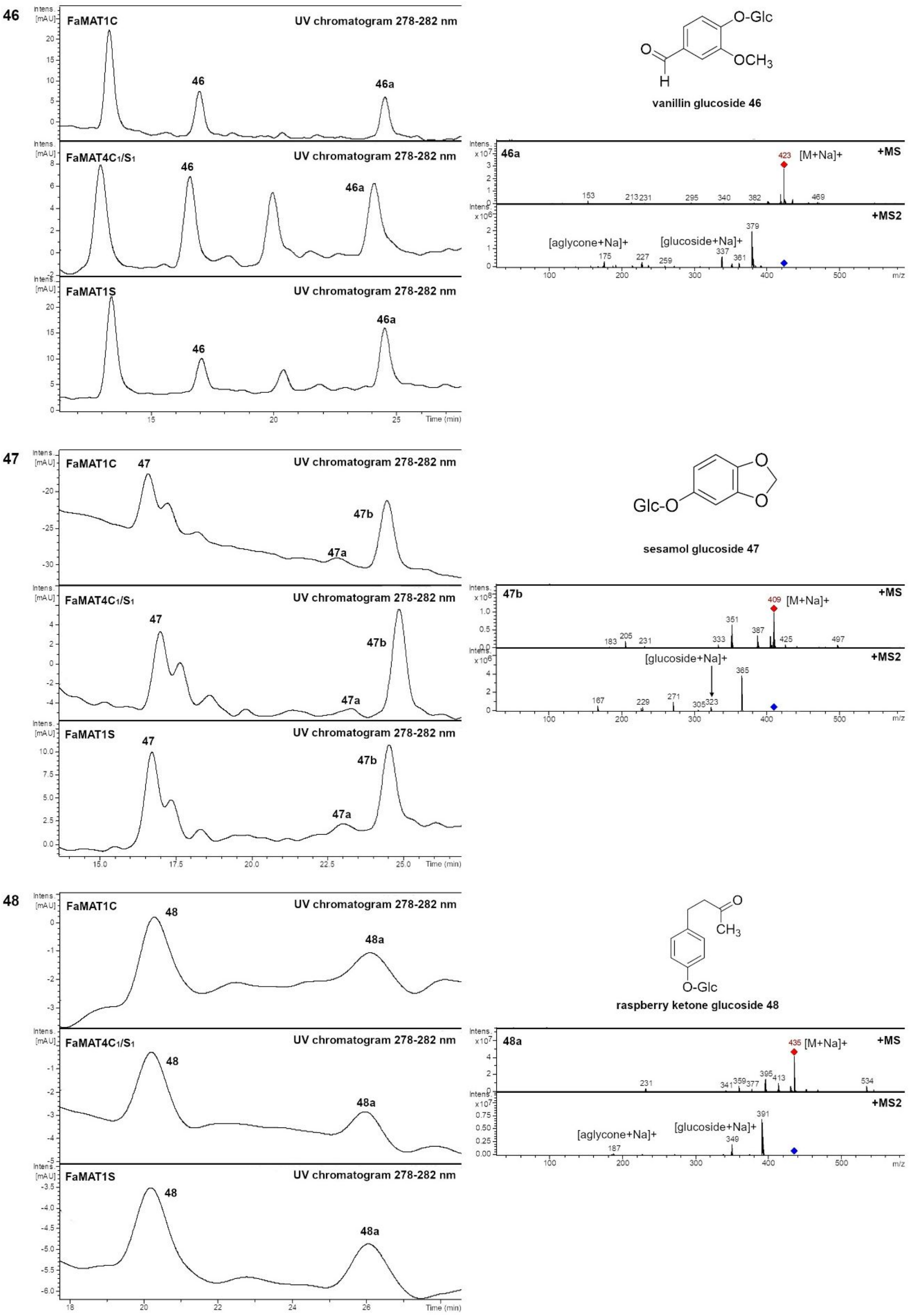

Figure S21. LC-MS analysis of the substrates and corresponding products. UV chromatogram of vanillin glucoside **46**, sesamol glucoside **47**, raspberry ketone glucoside **48**, and their malonylated products **46a**, **47a**, **47b**, and **48a**, respectively. Mass spectra (MS) and product ion spectra (MS2) of the malonylated products are shown on the right-hand side. + positive mode, - negative mode. Pseudo-molecular ions are marked in red and blue.

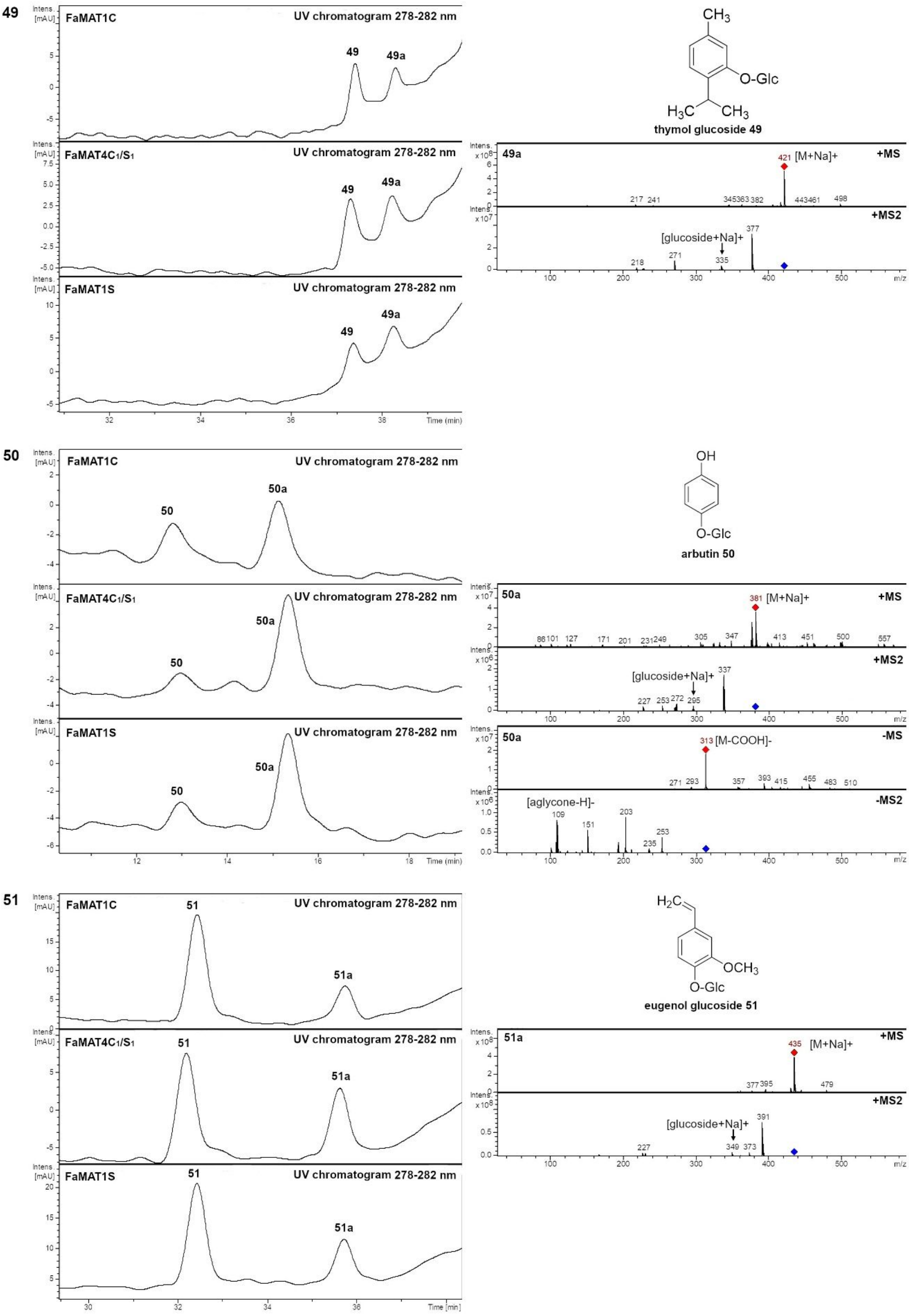

Figure S22. LC-MS analysis of the substrates and corresponding products. UV chromatogram of thymol *O*-glucoside **49**, arbutin **50**, eugenol *O*-glucoside **51**, and their malonylated products **49a**, **50a**, and **51a**, respectively. Mass spectra (MS) and product ion spectra (MS2) of the malonylated products are shown on the right-hand side. + positive mode, - negative mode. Pseudo-molecular ions are marked in red and blue.

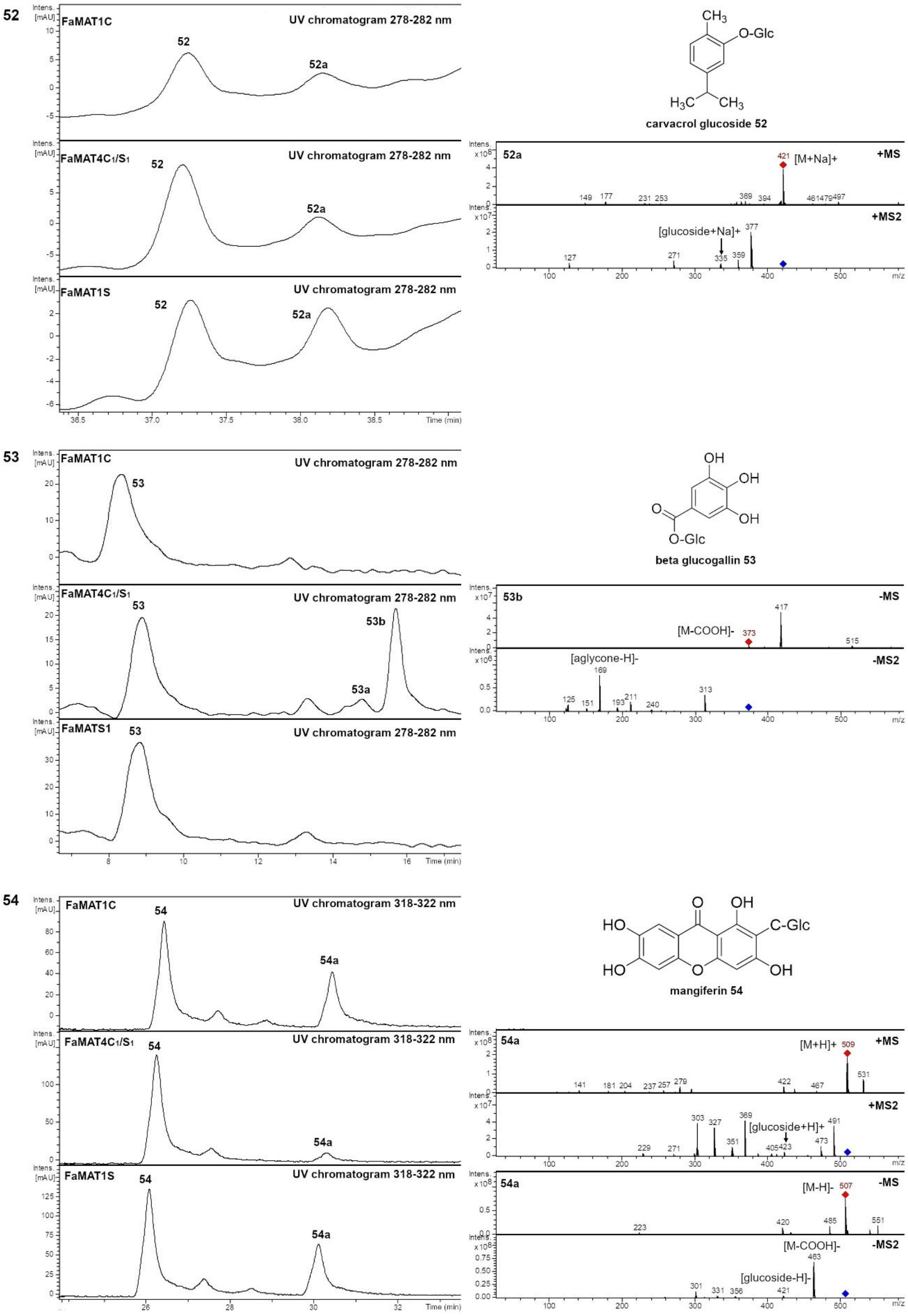

Figure S23. LC-MS analysis of the substrates and corresponding products. UV chromatogram of carvacrol *O*-glucoside **52**, β-glucogallin **53**, mangiferin **54**, and their malonylated products **52a**, **53a**, **53b**, and **54a**, respectively. Mass spectra (MS) and product ion spectra (MS2) of the malonylated products are shown on the right-hand side. + positive mode, - negative mode. Pseudo-molecular ions are marked in red and blue.

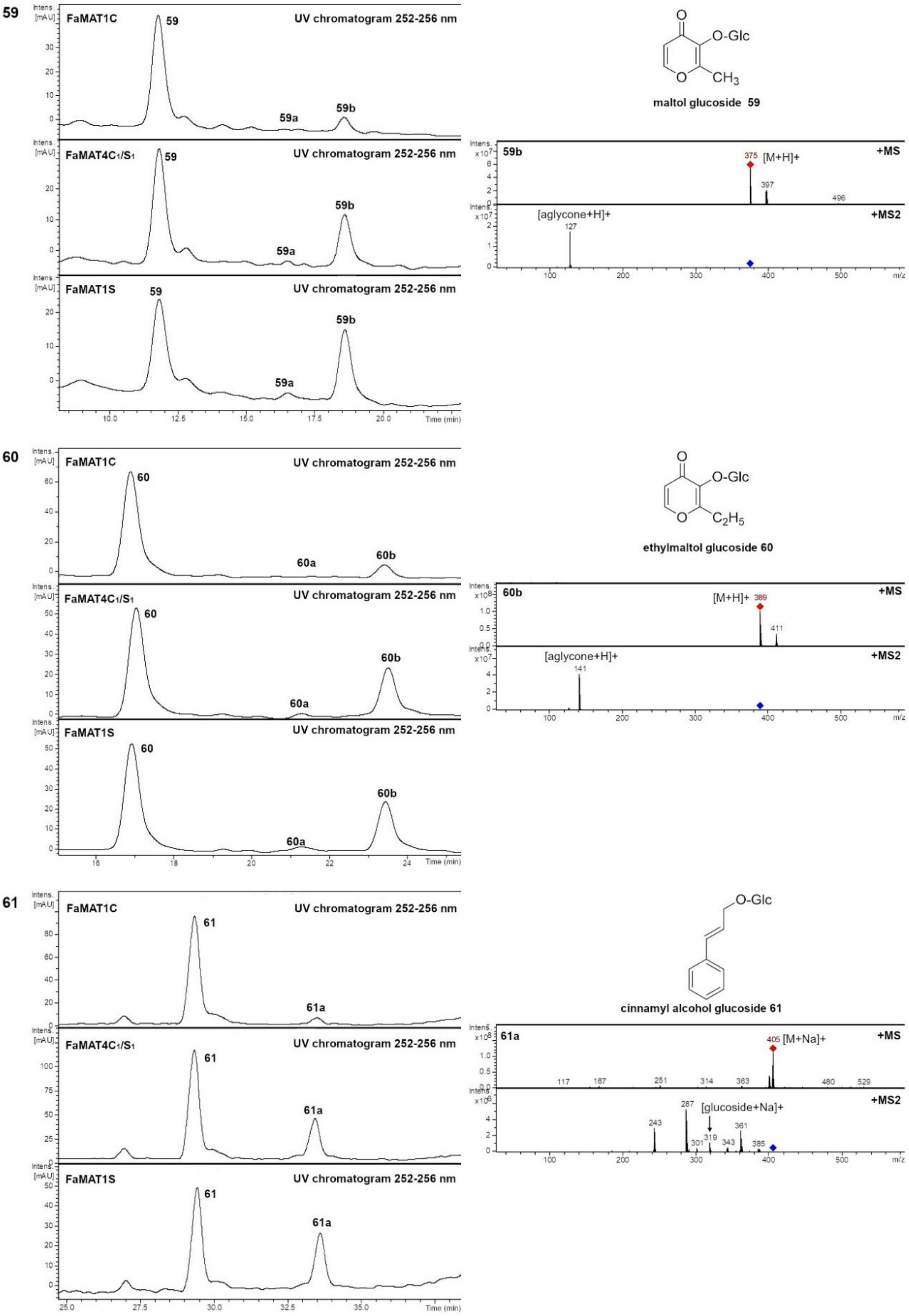

Figure S24. LC-MS analysis of the substrates and corresponding products. UV chromatogram of maltol *O*-glucoside **59**, ethylmaltol *O*-glucoside **60**, cinnamyl alcohol *O*-glucoside **61** and their malonylated products **59a, 59b**, **60a**, **60b**, and **61a,** respectively. Mass spectra (MS) and product ion spectra (MS2) of the malonylated products are shown on the right-hand side. + positive mode, - negative mode. Pseudo-molecular ions are marked in red and blue.

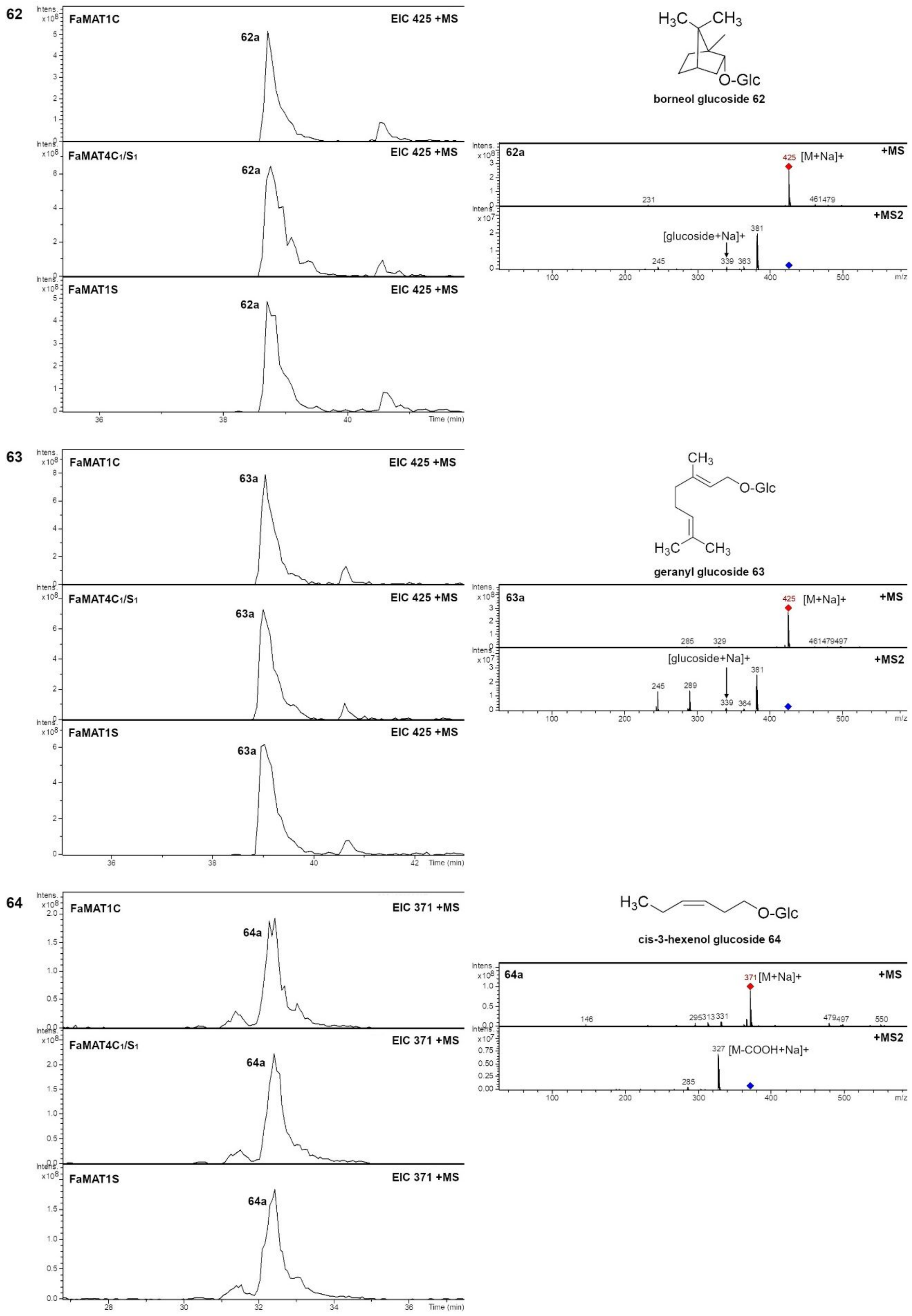

Figure S25. LC-MS analysis of the substrates and corresponding products. Extracted ion chromatogram (EIC) of borneol malonyl glucoside **62a,** geranyl malonyl glucoside **63a**, and cis-3-hexenol malonyl glucoside **64a**. Mass spectra (MS) and product ion spectra (MS2) of the malonylated products are shown on the right-hand side. + positive mode, - negative mode. Pseudo-molecular ions are marked in red and blue.

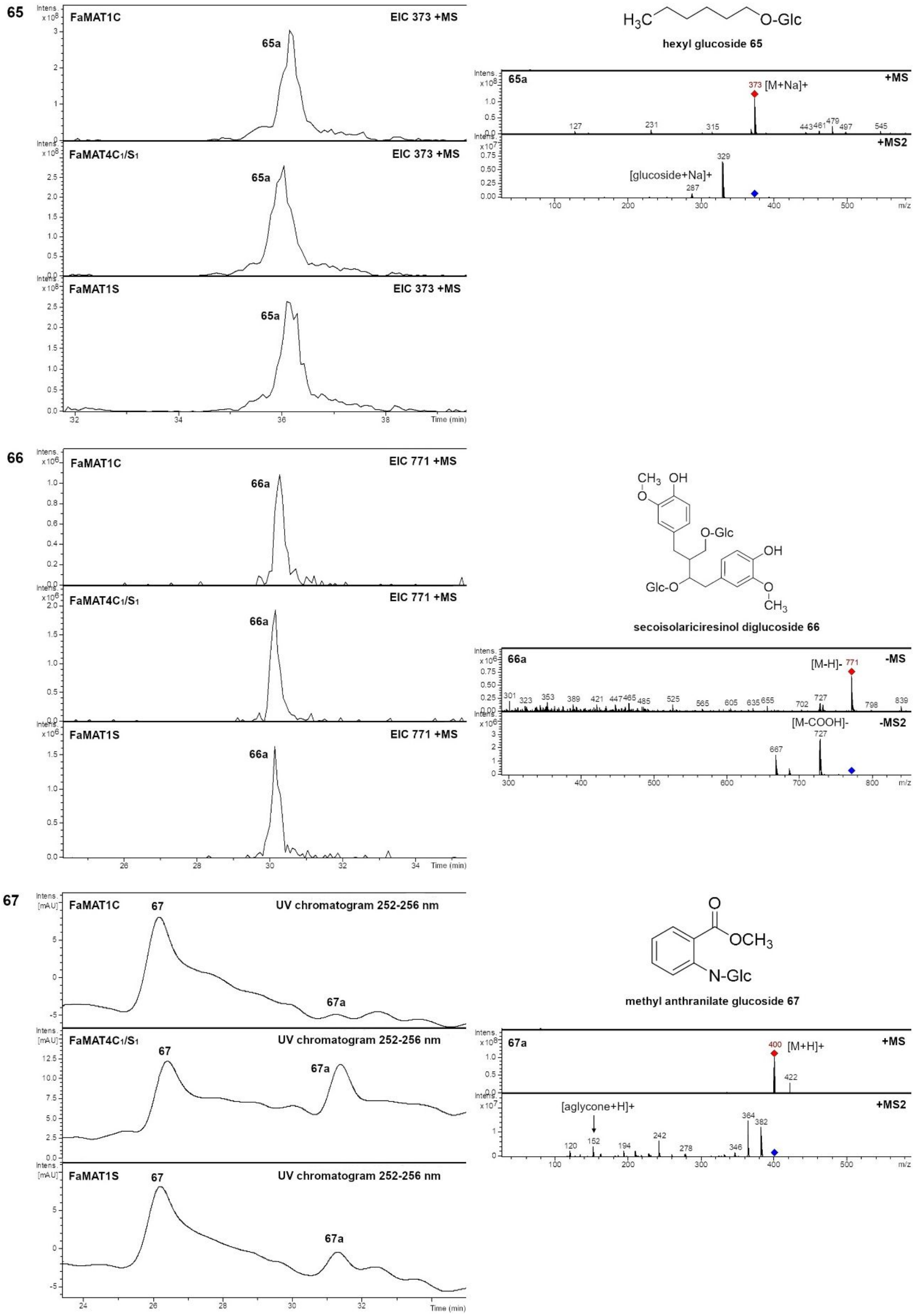

Figure S26. LC-MS analysis of the substrates and corresponding products. Extracted ion chromatogram (EIC) of hexyl malonyl glucoside **65a** and malonylated secoisolariciresinol diglucoside **66a** (mono malonyl). UV chromatogram of methyl anthranilate *N*-glucoside **67** and its malonylated product **67a**. Mass spectra (MS) and product ion spectra (MS2) of the malonylated products are shown on the right-hand side. + positive mode, - negative mode. Pseudo-molecular ions are marked in red and blue.

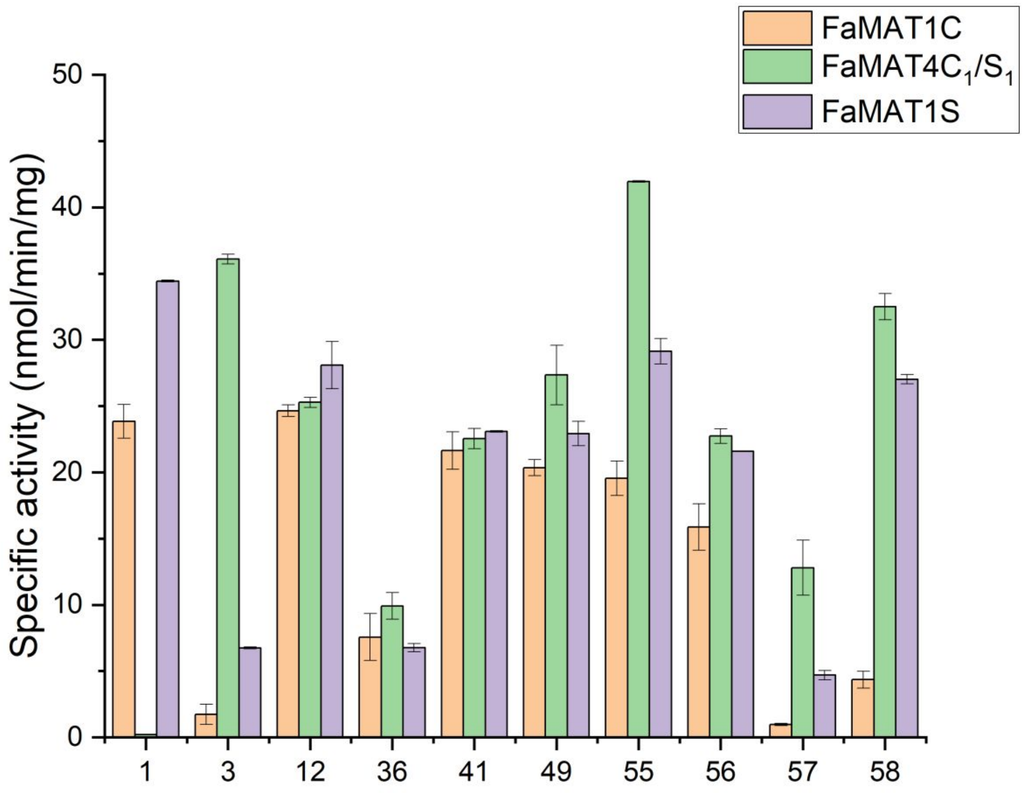

Figure S27. Specific activity of FaMATs towards apigenin-6-*C*-glucoside **1**, apigenin-7-*O*-glucoside **3**, quercetin-3-*O*-glucoside **12**, pelargonidin-3-*O*-glucoside **36**, aesculin **41**, thymol *O*-glucoside **49**, 5-EHMF *O*-glucoside **55**, HDMF *O*-glucoside **56**, sotolon *O*-glucoside **57**, and maple furanone *O*-glucoside **58**. The reactions were carried out with FaMATs (25 µg) at 30 °C and pH 7.0 for 30 minutes, measuring the initial rate of activity.

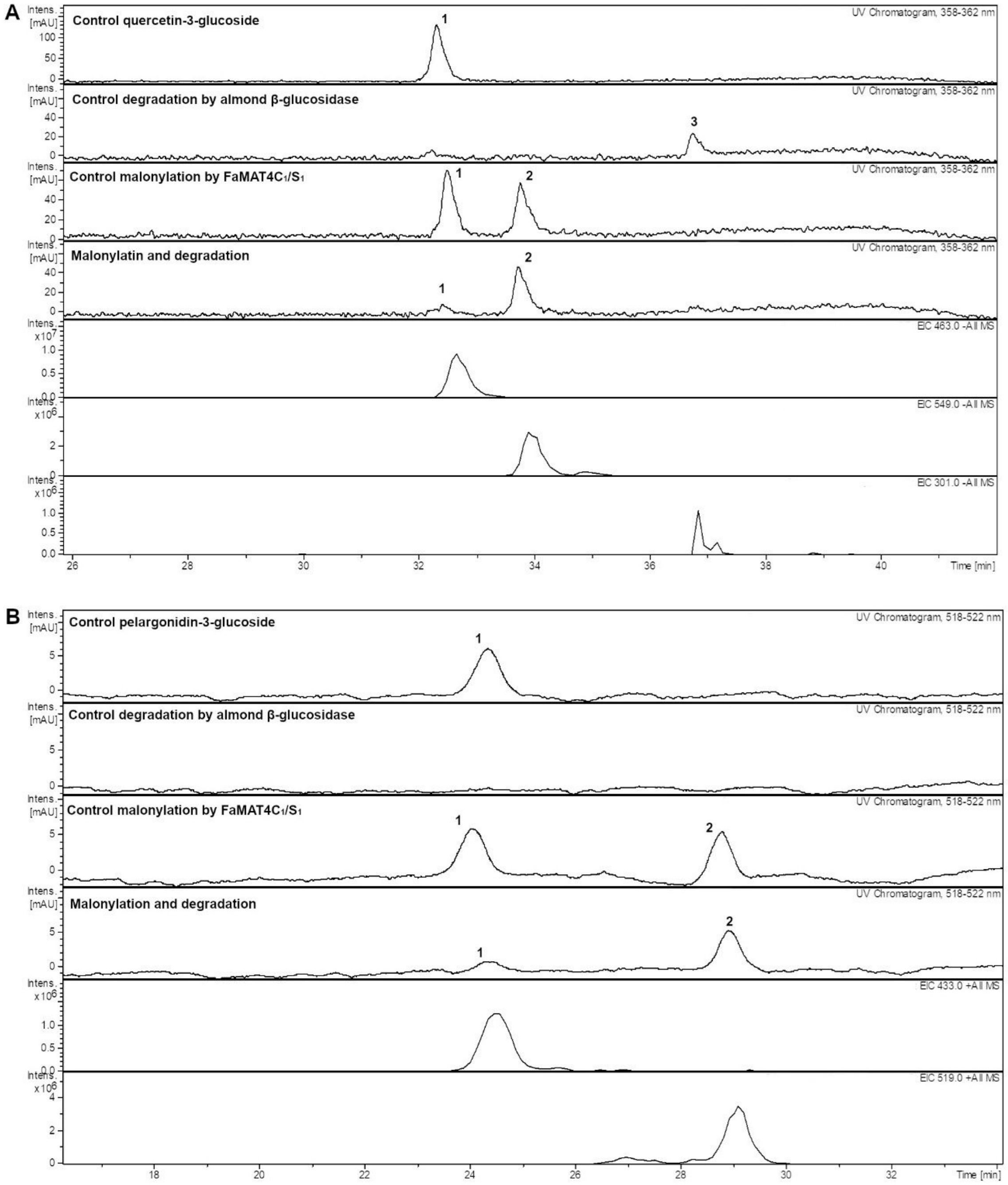

Figure S28. LC-MS analysis to investigate the stability of quercetin 3-*O*-(6''-*O*-malonyl)-glucoside (A) and pelargonidin 3-*O*-(6''-*O*-malonyl)-glucoside (B) against almond β-glucosidase. In A, compound 1: quercetin 3-*O*-glucoside *m/z* 463 $[M-H]^-$, compound 2: quercetin 3-*O*-(6''-*O*-malonyl)-glucoside *m/z* 549 $[M-H]^-$, and compound 3: quercetin *m/z* 301 $[M-H]^-$. In B, compound 1: pelargonidin 3-*O*-glucoside *m/z* 433 $[M]^+$, compound 2: pelargonidin 3-*O*-(6''-*O*-malonyl)-glucoside *m/z* 519 $[M]^+$. Pelargonidin could not be detected, probably due to instability.

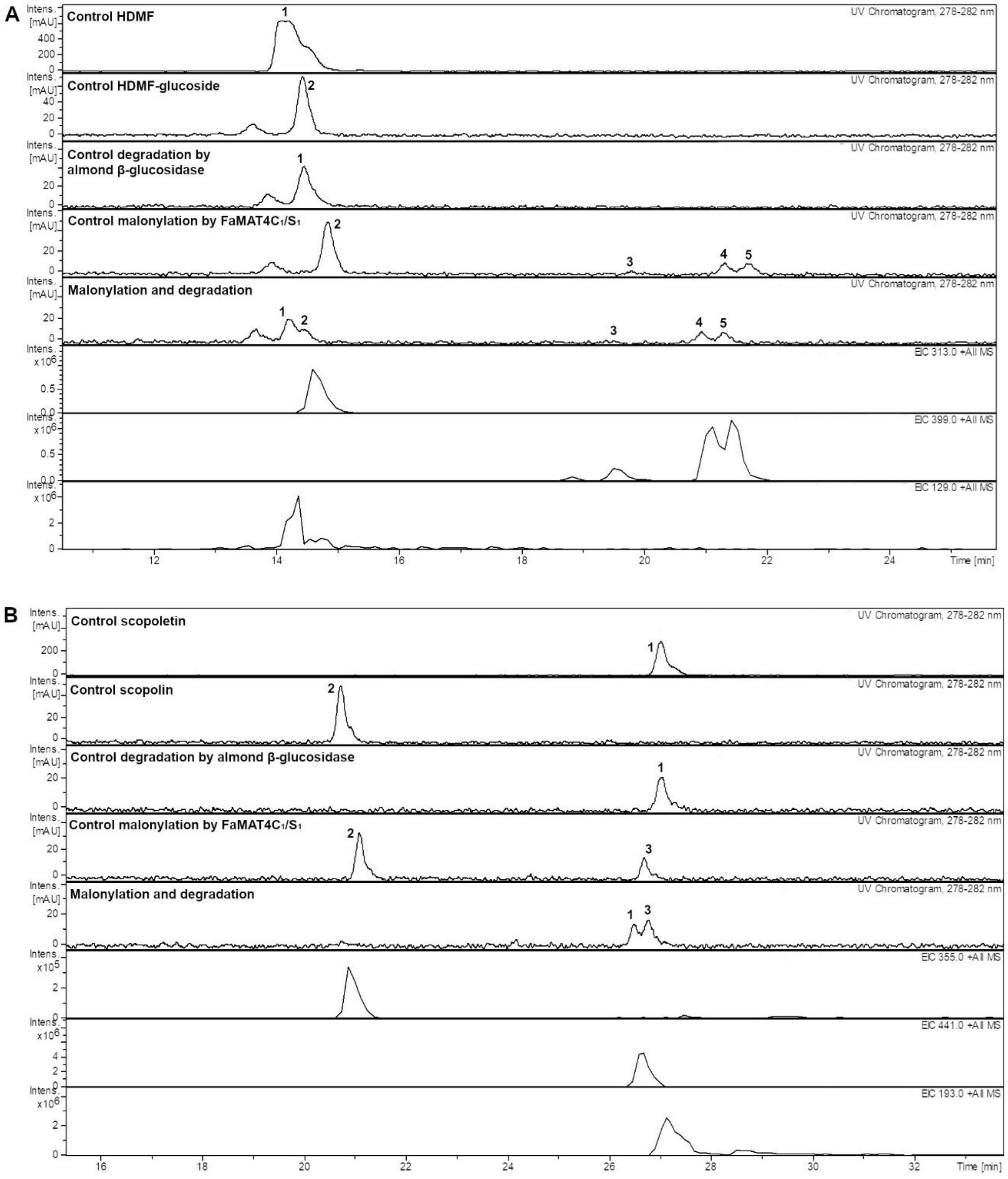

Figure S29. LC-MS analysis to investigate the stability of HDMF (6'-*O*-malonyl) glucoside (A) and malonylated scopolin (B) against almond β-glucosidase. In A, compound 1: HDMF *m/z* 129 $[M+H]^+$, compound 2: HDMF glucoside *m/z* 313 $[M+Na]^+$, compounds 3, 4, and 5: isomers of HDMF malonyl glucoside *m/z* 399 $[M+Na]^+$. In B, compound 1: scopoletin *m/z* 193 $[M+H]^+$, compound 2: scopolin *m/z* 355 $[M+H]^+$, compound 3: malonylated scopolin *m/z* 441 $[M+H]^+$.

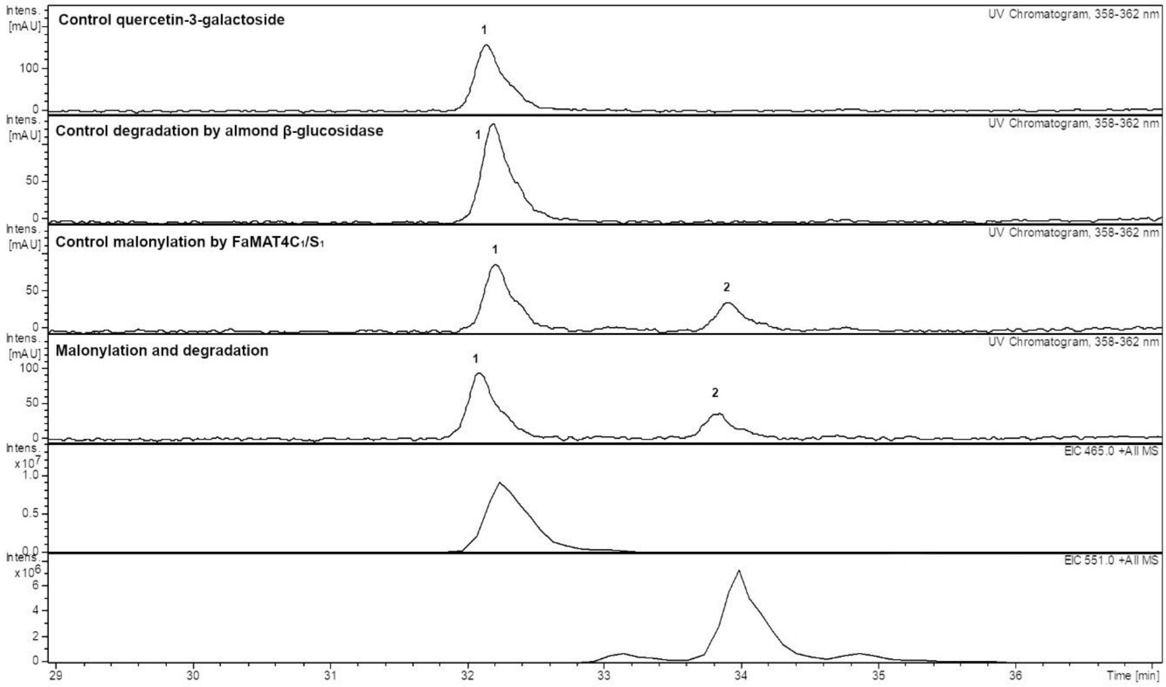

Figure S30. LC-MS analysis to investigate the stability of quercetin-3-*O*-(6'-*O*-malonyl)-galactoside against almond β-glucosidase. Compound 1: quercetin-3-*O*-galactoside *m/z* 465 $[M+H]^+$, compound 2: quercetin-3-*O*-(6'-*O*-malonyl)-galactoside *m/z* 551 $[M+H]^+$.

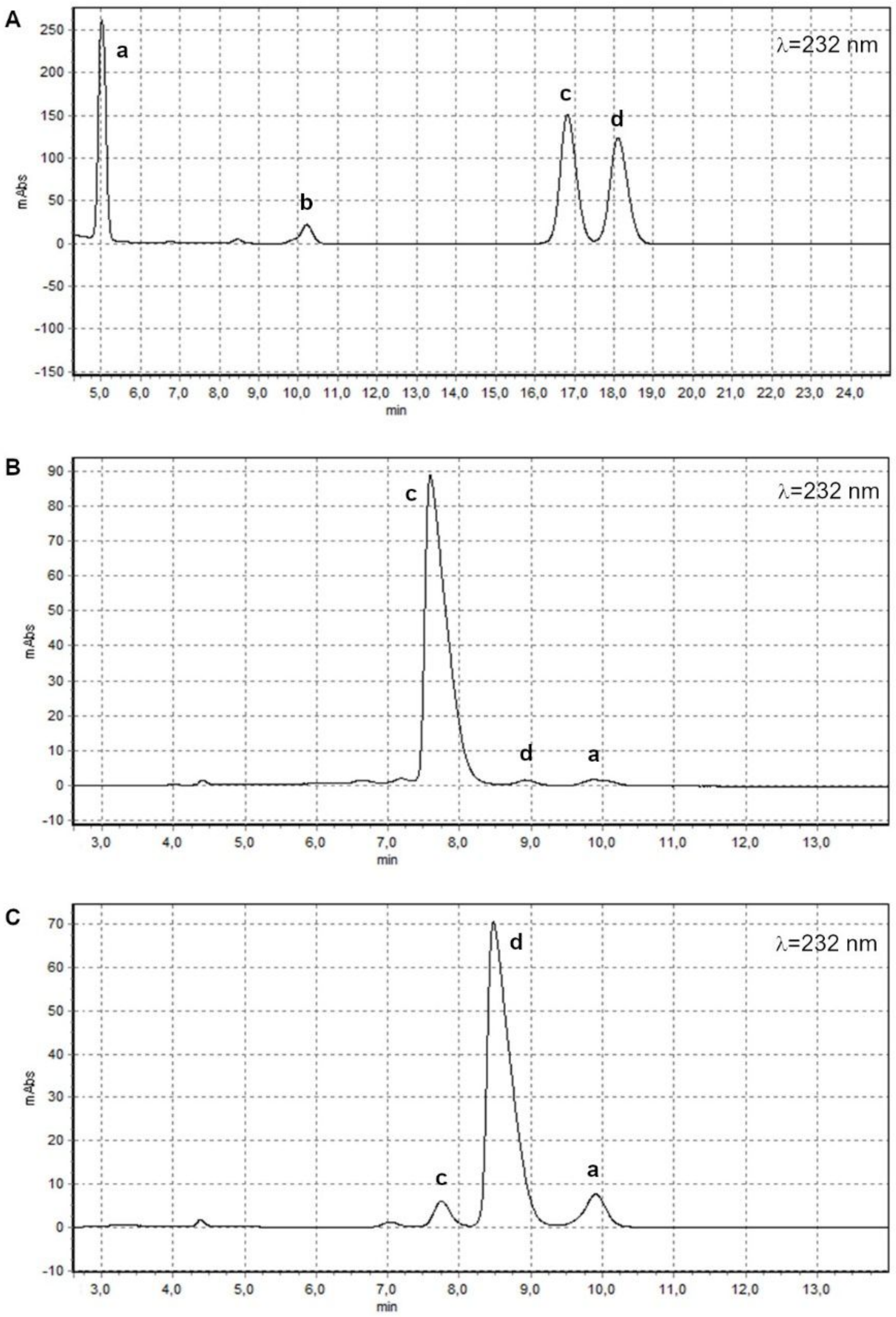

Figure S31. UV chromatogram of malonylated products formed from maple furanone glucoside. Compound **a**: maple furanone glucoside, **b**: small isomer maple furanone malonyl glucoside (not isolated), **c**: isomer 1 maple furanone malonyl glucoside, **d**: isomer 2 maple furanone glucoside. In A: first separation, conditions: column Zorbax Eclipse XDB-C8 (150 x 4.6 mm), isocratic mobile phase of 5% C_2H_5OH with 0.1% HCOOH pH 3.04, flow rate 1.5 ml min^{-1}, λ = 232 nm, and sample injection volume 10 µL. In B: second separation of isomer 1 maple furanone malonyl glucoside. In **C**: second separation of isomer 2 maple furanone malonyl glucoside. The conditions in B and C are as follows: column Zorbax Eclipse XDB-C8 (150 x 4.6 mm), isocratic mobile phase of 2% C_2H_5OH with 10 mM NH_4HCOO pH 6.45, flow rate 1.5 ml min^{-1}, λ = 232 nm, and sample injection volume 10 µL. In the second separation, malonylated products isomer 1 (**c**) and isomer 2 (**d**) elute earlier as they are ionized at high pH and more polar than the maple furanone glucoside (**a**).

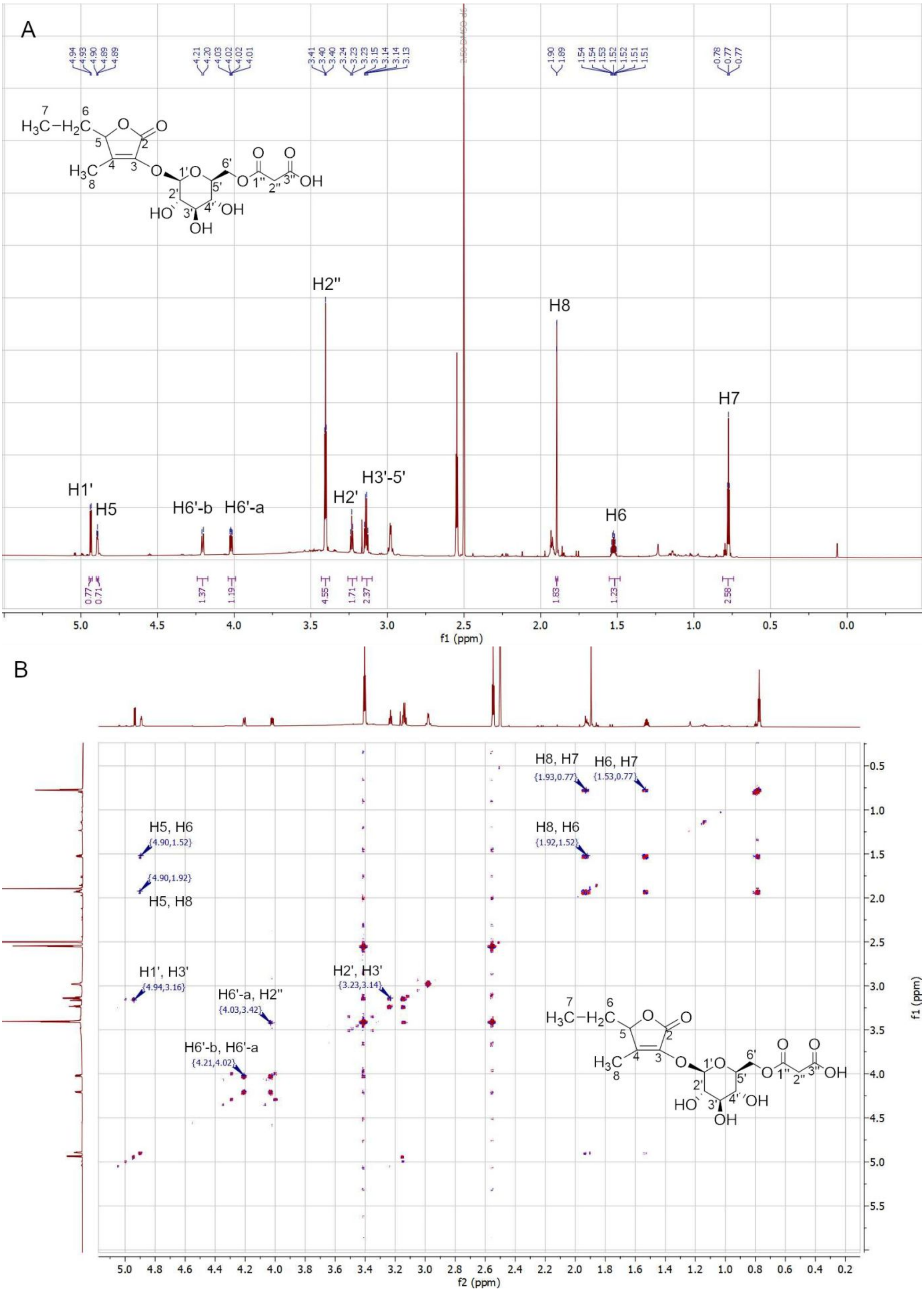

Figure S32. ^{1}H-NMR spectrum (A) and COSY (B) of maple furanone malonyl glucoside isomer 1.

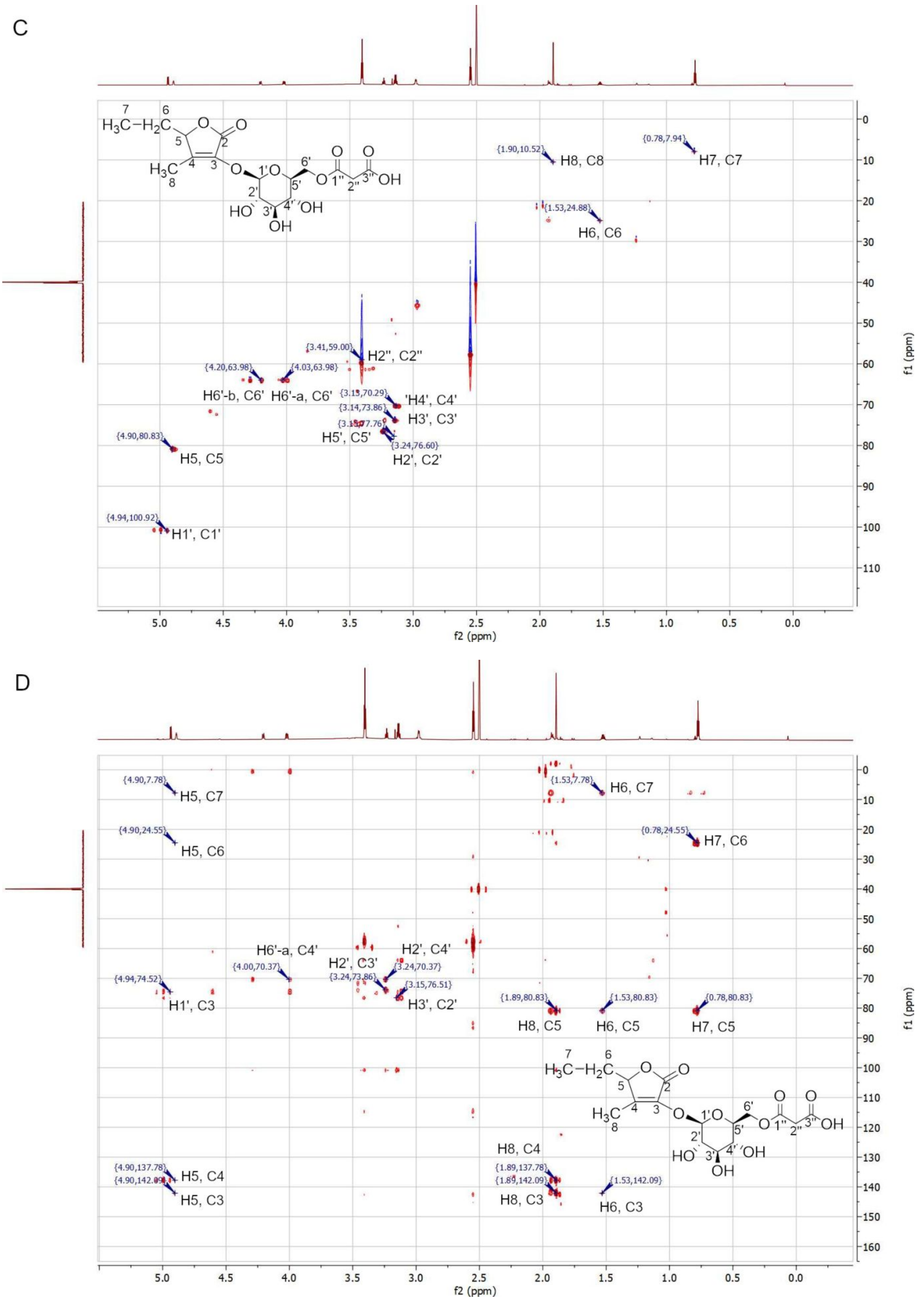

Figure S33. HSQC (C) and HMBC (D) of maple furanone malonyl glucoside isomer 1.

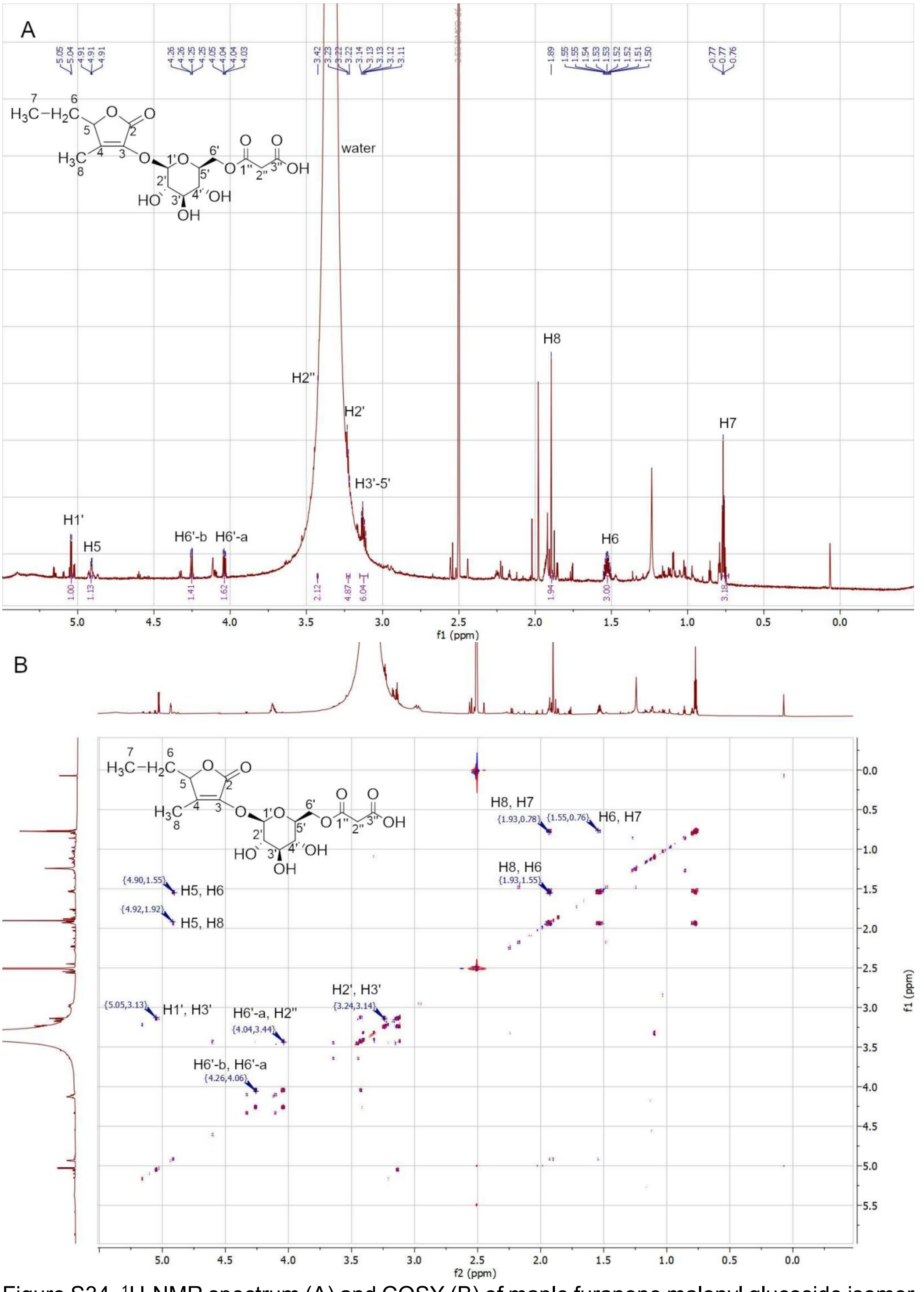

Figure S34. ^{1}H-NMR spectrum (A) and COSY (B) of maple furanone malonyl glucoside isomer 2.

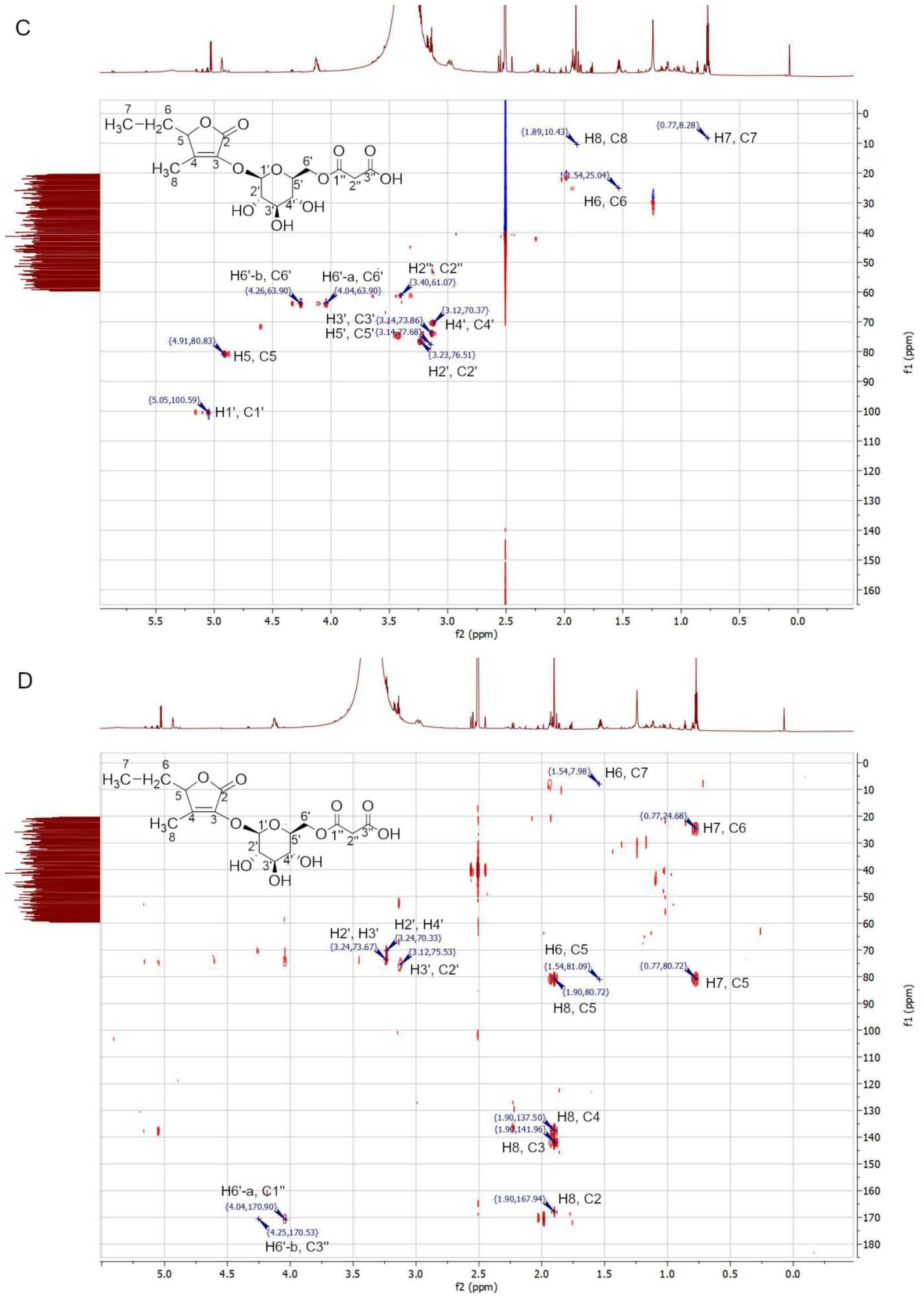

Figure S35. HSQC (C) and HMBC (D) of maple furanone malonyl glucoside isomer 2.

Supplementary Data 1.

Molecular docking predicted the binding sites of FaMATs and malonyl-CoA and HDMF glucoside

The structures of FaMATs protein were constructed using AlphaFold2.[3] The protein models were evaluated by Ramachandran plot using PROCHECK analysis from SAVES.[4] The secondary structures of protein were predicted by PDBsum.[5] AutoDock Tools 1.5.7 was utilized to add hydrogen and Kollman charges for protein and Gasteiger-Marsili charges for the ligands.[6,7] The binding pockets of malonyl-CoA and acyl acceptors for the docking grid box were determined by aligning the binding sites from Dm3MaT1.[8] Molecular docking of protein and ligands was conducted using AutoDock Vina.[9] Docking results were visualized using ChimeraX.

The predicted structures of FaMAT1C, FaMAT1S, and FaMAT4C_1/S_1, generated using AlphaFold2, exhibited high confidence based on their pLDDT (predicted Local Distance Difference Test) scores: 91.1, 91.6, and 92.6, respectively. A pLDDT score ≥ 90 indicates very high model confidence.[10] Further validation using Ramachandran plots revealed that 89.0%, 90.1%, and 92.1% of residues for FaMAT1C, FaMAT1S, and FaMAT4C_1/S_1, respectively, were located in the most favored regions, supporting the reliability of the predicted protein models (Figure S34).

Each FaMAT protein model contains 22 α-helices and 17 β-strands (Figure S35). Structural analysis revealed that the conserved motifs HXXXD and YFGNC are located near the solvent channel, whereas the DFGWG motif is positioned away from it (Figure S36). Molecular docking results indicated that the active ligand-binding pocket is connected to the solvent channel. Hydrogen bonding analysis showed that FaMAT1C, FaMAT1S, and FaMAT4C_1/S_1 form 10, 8, and 11 hydrogen bonds with malonyl-CoA, respectively. In FaMAT1C, malonyl-CoA interacts via hydrogen bonds with residues His164, Gly169, Pro315, Ser320, Arg321, Ser418, and Thr422. For FaMAT1S, key interacting residues include His164, Lys170, His283, Asp316, Ser319, and Arg397. In FaMAT4C_1/S_1, malonyl-CoA forms hydrogen bonds with Arg45, His169, Ser316, Asp318, Ser321, Arg322, Glu329, Ser400, and

Arg402. Notably, the NE2 atom of His164 (in FaMAT1C and FaMAT1S) and His169 (in FaMAT4C$_1$/S$_1$), part of the HXXXD motif, forms a hydrogen bond with the thioester carbonyl oxygen of the malonyl moiety. This interaction mirrors that observed in Dm3MaT3, where His170 from the HXXXD motif engages the same site on malonyl-CoA.[8]

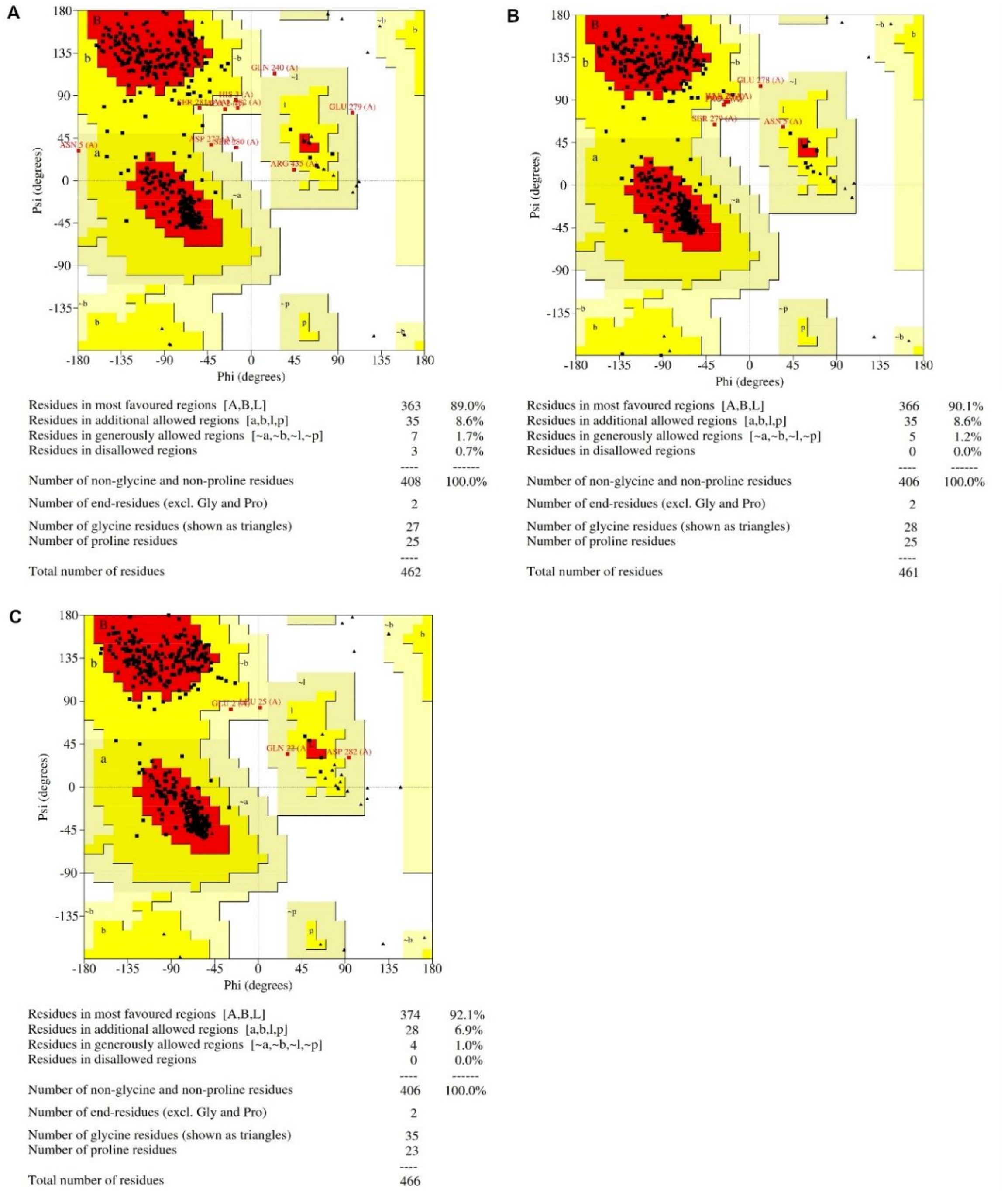

Figure S36. Ramachandran plot of FaMAT1C (A), FaMAT1S (B), FaMAT4C$_1$/S$_1$ (C) protein model.

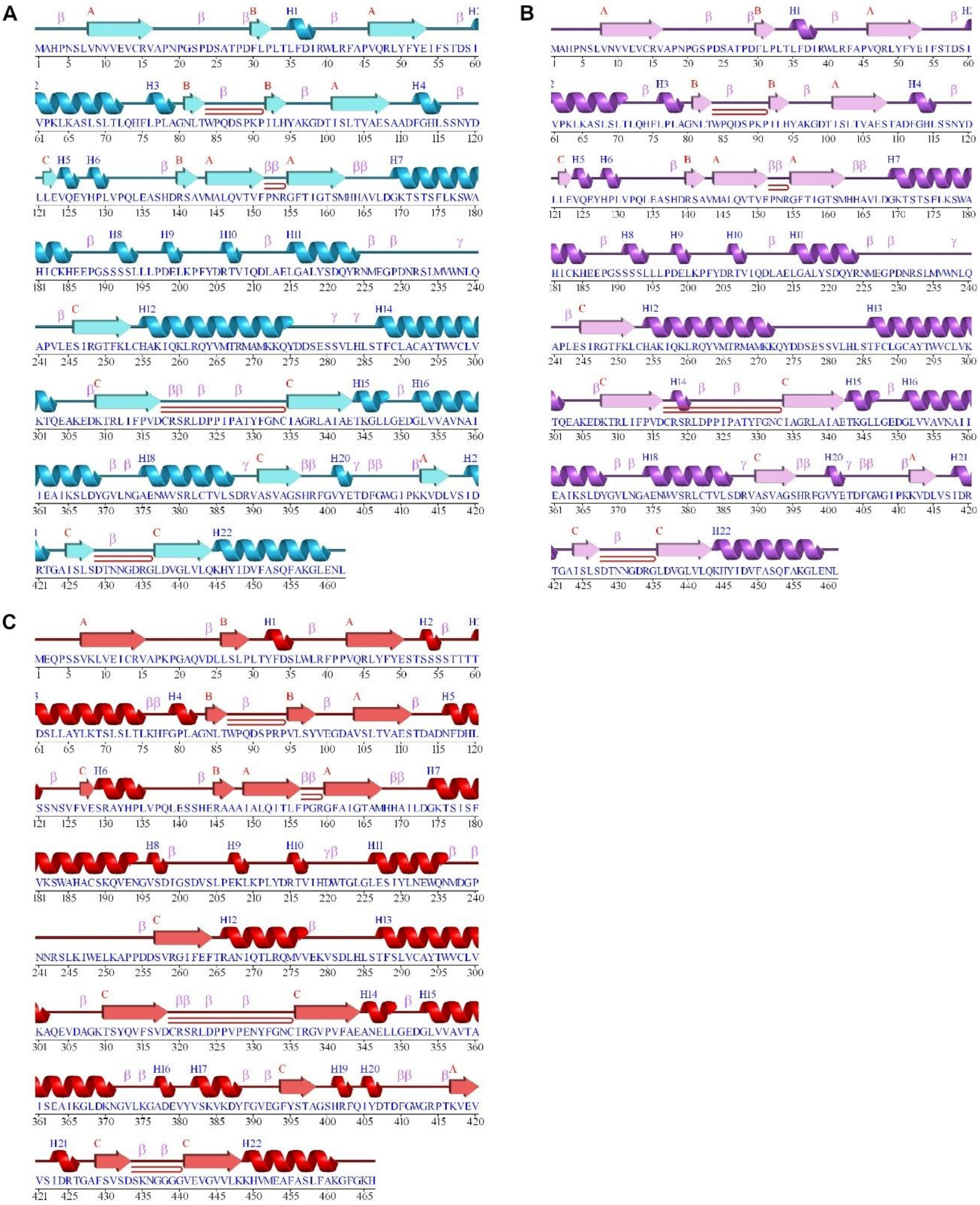

Figure S37. Prediction of secondary structures (α-helices and β-sheets) and motifs (β turn, γ turn, and hairpin) of FaMAT1C (A), FaMAT1S (B), FaMAT4C$_1$/S$_1$ (C). Helices are labeled as H1, H2, etc. Strands are labeled as A, B, etc. The prediction was carried out at www.ebi.ac.uk/thornton-srv/databases/pdbsum/.

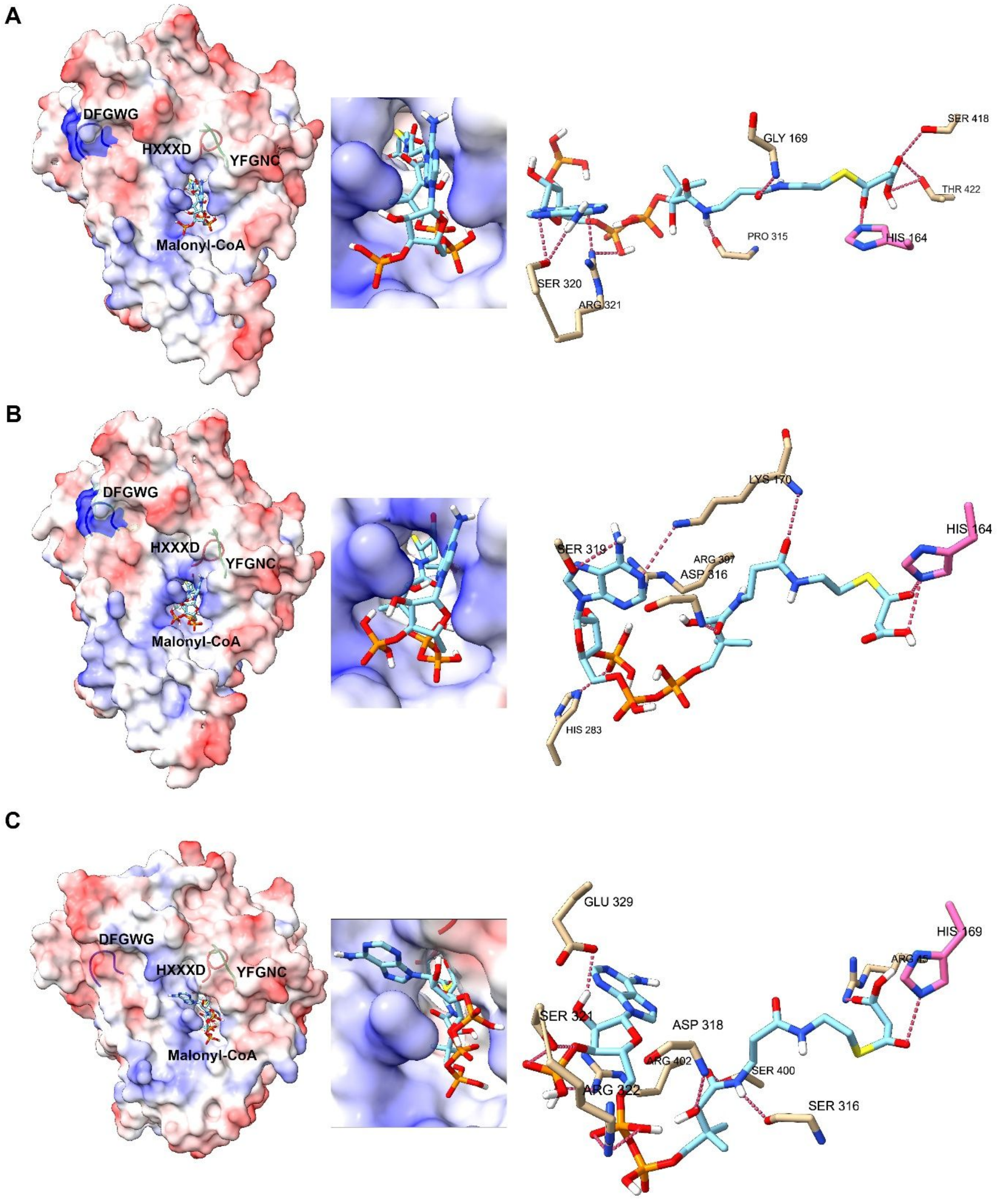

Figure S38. Docking experiments. FaMAT1C (A), FaMAT1S (B), and FaMAT4C$_1$/S$_1$ (C) docking with malonyl-CoA, surface presentation, and hydrogen bond interactions between protein and malonyl-CoA. Red, green, and blue sticks in the protein structures represent HXXXD, YFGNC, and DFGWG motifs, respectively.

Molecular docking was performed to evaluate the interaction of FaMAT proteins with HDMF glucoside as the acyl acceptor. The binding pocket for HDMF glucoside is located on the opposite face of the protein relative to the malonyl-CoA binding site (Figure S37).

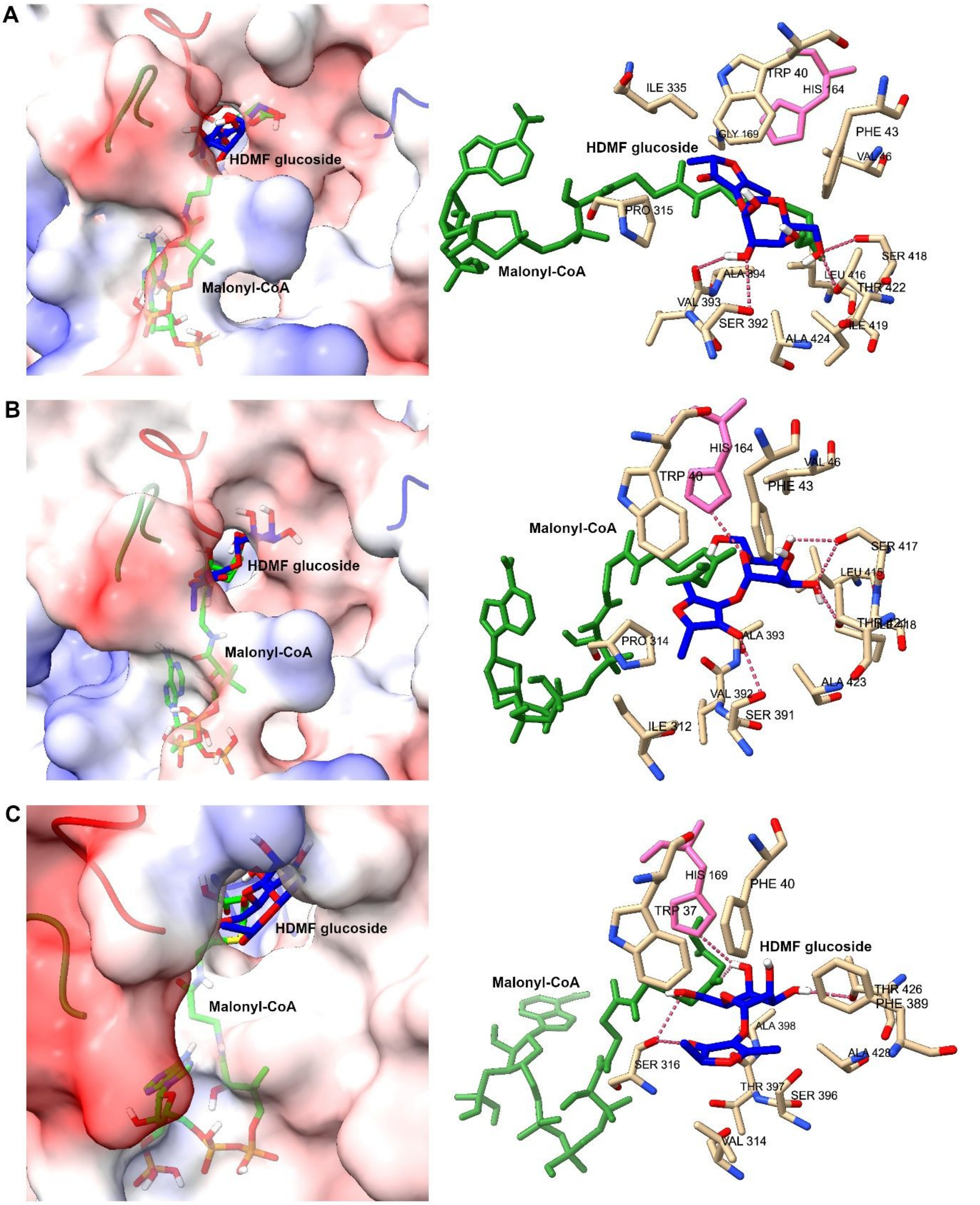

Figure S39. FaMAT1C (A), FaMAT1S (B), and FaMAT4C$_1$/S$_1$ (C) docking with malonyl-CoA and HDMF glucoside. Blue sticks are HDMF glucoside; green sticks are malonyl-CoA. Hydrogen bonds are shown by red dashed lines.

In **FaMAT1C**, the HDMF glucoside binding pocket includes residues: Trp40, Phe43, Val46, His164, Gly169, Pro315, Ile335, Ser392, Val393, Ala394, Leu416, Ser418, Ile419, Thr422, and Ala424. In **FaMAT1S**, the pocket comprises Trp40, Phe43, Trp46, His164, Ile312, Pro314, Ser391, Val392, Ala393, Leu415, Ser417, Ile418, Thr421, and Ala423. In **FaMAT4C1/S1**, the binding site includes Trp37, Phe40, His169, Val314, Ser316, Phe389, Ser396, Thr397, Ala398, Thr426, and Ala428. Notably, the conserved His residue from the HXXXD motif is present in the acyl acceptor binding pocket across all FaMATs. This histidine plays a critical catalytic role by deprotonating a hydroxyl group on the glucosyl moiety, generating a nucleophile that attacks the thioester carbonyl of malonyl-CoA, thereby facilitating malonyl group transfer.[11]

References

(1) Nakahashi, A.; Yaguchi, Y.; Miura, N.; Emura, M.; Monde, K. A Vibrational Circular Dichroism Approach to the Determination of the Absolute Configurations of Flavorous 5-Substituted-2(5 H)-Furanones. *J Nat Prod* **2011**, *74* (4), 707–711.

(2) Monde, K.; Nakahashi, A.; Miura, N.; Yaguchi, Y.; Sugimoto, D.; Emura, M. Stereochemical Study of a Novel Tautomeric Furanone, Homofuraneol. *Chirality* **2009**, *21* (1 E).

(3) Mirdita, M.; Schütze, K.; Moriwaki, Y.; Heo, L.; Ovchinnikov, S.; Steinegger, M. ColabFold: Making Protein Folding Accessible to All. *Nat Methods 2022 19:6* **2022**, *19* (6), 679–682.

(4) Laskowski, R. A.; MacArthur, M. W.; Moss, D. S.; Thornton, J. M. PROCHECK: A Program to Check the Stereochemical Quality of Protein Structures. *J Appl Crystallogr* **1993**, *26* (2), 283–291.

(5) Laskowski, R. A. PDBsum: Summaries and Analyses of PDB Structures. *Nucleic Acids Res* **2001**, *29* (1).

(6) Singh, U. C.; Kollman, P. A. An Approach to Computing Electrostatic Charges for Molecules. *J Comput Chem* **1984**, *5* (2), 129–145.

(7) Gasteiger, J.; Marsili, M. Iterative Partial Equalization of Orbital Electronegativity—a Rapid Access to Atomic Charges. *Tetrahedron* **1980**, *36* (22), 3219–3228.

(8) Unno, H.; Ichimaida, F.; Suzuki, H.; Takahashi, S.; Tanaka, Y.; Saito, A.; Nishino, T.; Kusunoki, M.; Nakayama, T. Structural and Mutational Studies of Anthocyanin Malonyltransferases Establish the Features of BAHD Enzyme Catalysis. *J Biol Chem* **2007**, *282* (21), 15812–15822.

(9) Eberhardt, J.; Santos-Martins, D.; Tillack, A. F.; Forli, S. AutoDock Vina 1.2.0: New Docking Methods, Expanded Force Field, and Python Bindings. *J Chem Inf Model* **2021**, *61* (8), 3891–3898.

(10) Varadi, M.; Anyango, S.; Deshpande, M.; Nair, S.; Natassia, C.; Yordanova, G.; Yuan, D.; Stroe, O.; Wood, G.; Laydon, A.; Zídek, A.; Green, T.; Tunyasuvunakool, K.; Petersen, S.; Jumper, J.; Clancy, E.; Green, R.; Vora, A.; Lutfi, M.; Figurnov, M.; Cowie, A.; Hobbs, N.; Kohli, P.; Kleywegt, G.; Birney, E.; Hassabis, D.; Velankar, S. AlphaFold Protein Structure Database: Massively Expanding the Structural Coverage of Protein-Sequence Space with High-Accuracy Models. *Nucleic Acids Res* **2022**, *50* (D1), D439–D444.

(11) Manjasetty, B. A.; Yu, X. H.; Panjikar, S.; Taguchi, G.; Chance, M. R.; Liu, C. J. Structural Basis for Modification of Flavonol and Naphthol Glucoconjugates by Nicotiana Tabacum Malonyltransferase (NtMaT1). *Planta* **2012**, *236* (3), 781–793.
